# A method for controlling the synthesis of stable twisted two-dimensional conjugated molecules

**DOI:** 10.1038/ncomms11637

**Published:** 2016-05-16

**Authors:** Yongjun Li, Zhiyu Jia, Shengqiang Xiao, Huibiao Liu, Yuliang Li

**Affiliations:** 1 CAS Key Laboratory of Organic Solids, Beijing National Laboratory for Molecular Science (BNLMS), Institute of Chemistry, Chinese Academy of Sciences, Beijing 100190, China; 2 State Key Laboratory of Advanced Technology for Materials Synthesis and Processing, Wuhan University of Technology, Wuhan 430070, China

## Abstract

Thermodynamic stabilization (π-electron delocalization through effective conjugation) and kinetic stabilization (blocking the most-reactive sites) are important considerations when designing stable polycyclic aromatic hydrocarbons displaying tunable optoelectronic properties. Here, we demonstrate an efficient method for preparing a series of stable two-dimensional (2D) twisted dibenzoterrylene-acenes. We investigated their electronic structures and geometries in the ground state through various experiments assisted by calculations using density functional theory. We find that the length of the acene has a clear effect on the photophysical, electrochemical, and magnetic properties. These molecules exhibit tunable ground-state structures, in which a stable open-shell quintet tetraradical can be transferred to triplet diradicals. Such compounds are promising candidates for use in nonlinear optics, field effect transistors and organic spintronics; furthermore, they may enable broader applications of 2D small organic molecules in high-performance electronic and optical devices.

## Abstract (web summary)

Polycyclic aromatic hydrocarbons with tunable optoelectronic properties promise applications in future high tech devices. Here, the authors demonstrate an efficient method for preparing a series of stable 2D twisted dibenzoterrylene-acenes, and investigate their electronic structures and geometries.

## Graphical abstract

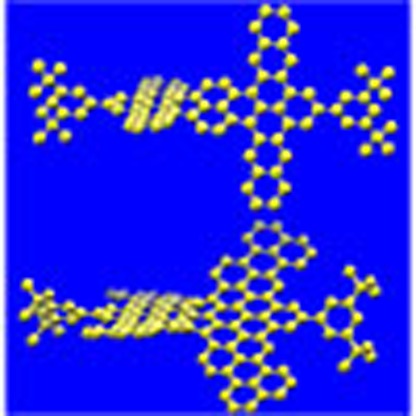

In the last two decades, methodologies for the controlled synthesis of large π-extended polycyclic aromatic hydrocarbons (PAHs) have been established using bottom-up principles^1^,^2^,^3^,^4^,^5^,^6^,^7^,^8^,^9^,^10^,^11^. The properties of PAHs depend heavily on their degrees of π-extension, shapes, widths and edge topologies^1^,^3^,^4^,^12^,^13^,^14^. Many linear acenes, including tetracene, pentacene and related derivatives, have been synthesized to produce highly desirable electronic properties, including remarkable charge-carrier mobilities^15^,^16^,^17^,^18^,^19^. Open-shell PAHs, which feature one or more π-electrons not tightly paired into the bonding molecular orbital in the ground state, are particularly interestingly structures that have properties very different from those of typical PAHs having closed-shell electronic structures^20^,^21^,^22^,^23^. The unique electronic structures of two-dimensional (2D) open-shell PAHs can impart attractive electronic, optical and magnetic properties for applications in materials science^24^,^25^,^26^.

Acenes become increasingly reactive as the number of rings increases, with the central ring being the most reactive^27^,^28^. As a result, the central rings in these molecules are susceptible to oxidation, photodegeneration and Diels–Alder reactions^29^,^30^. Open-shell molecules are particularly vulnerable to degradation reactions^22^,^23^; therefore, instability remains a key obstacle affecting their practical applications. In most cases, thermodynamic stabilization (π-electron delocalization through effective conjugation) and kinetic stabilization (blocking of the most-reactive sites) are both necessary to obtain stable materials^18^,^31^. One strategy is to annulate the aromatic rings onto two neighbouring rings, creating 2D acene analogues^15^. Changing from the linear character of a condensed array (peri-condensed) to an angular geometry (cata-condensed) results in at least two sextets of π-electrons, increasing the stability relative to that of a linear analogue having only one sextet^32^. Tuning a planar structure to a nonplanar structure through twisting or saddling can also enhance the kinetic stability as a result of steric blocking^21^,^33^.

2D acene analogues that represent various fragments of graphene have received significant attention because of their electronic properties^6^,^9^,^10^,^15^. Molecules of this class have large, planar π-surfaces allowing high-intermolecular surface overlap^34^,^35^,^36^. The packing of such molecules is dictated by multiple interactions that can effectively increase the dimensionality of the electronic structure, leading to enhanced transport properties^37^,^38^. Many 2D planar PAHs^11^,^32^,^34^,^36^,^39^,^40^,^41^ and contorted polycyclic aromatics^5^,^42^,^43^,^44^ have been synthesized and characterized recently.

In this paper, we report the transformation of a one-dimensional (1D) single conjugated dibenzoterrylene through the attachment of conjugated acene ‘arms' to generate a 2D molecular structure. High dimensionality within the single molecule was assured by hybridizing dibenzoterrylene with acenes of different conjugation lengths; stability was provided by benzoannulating the active rings of acenes and inducing contortions from the cove or fjord regions. This protocol is applicable to construct various 2D twisted nanographene-like structures in a rapid manner.

## Results

### Design of target compounds

**Figure 1:**
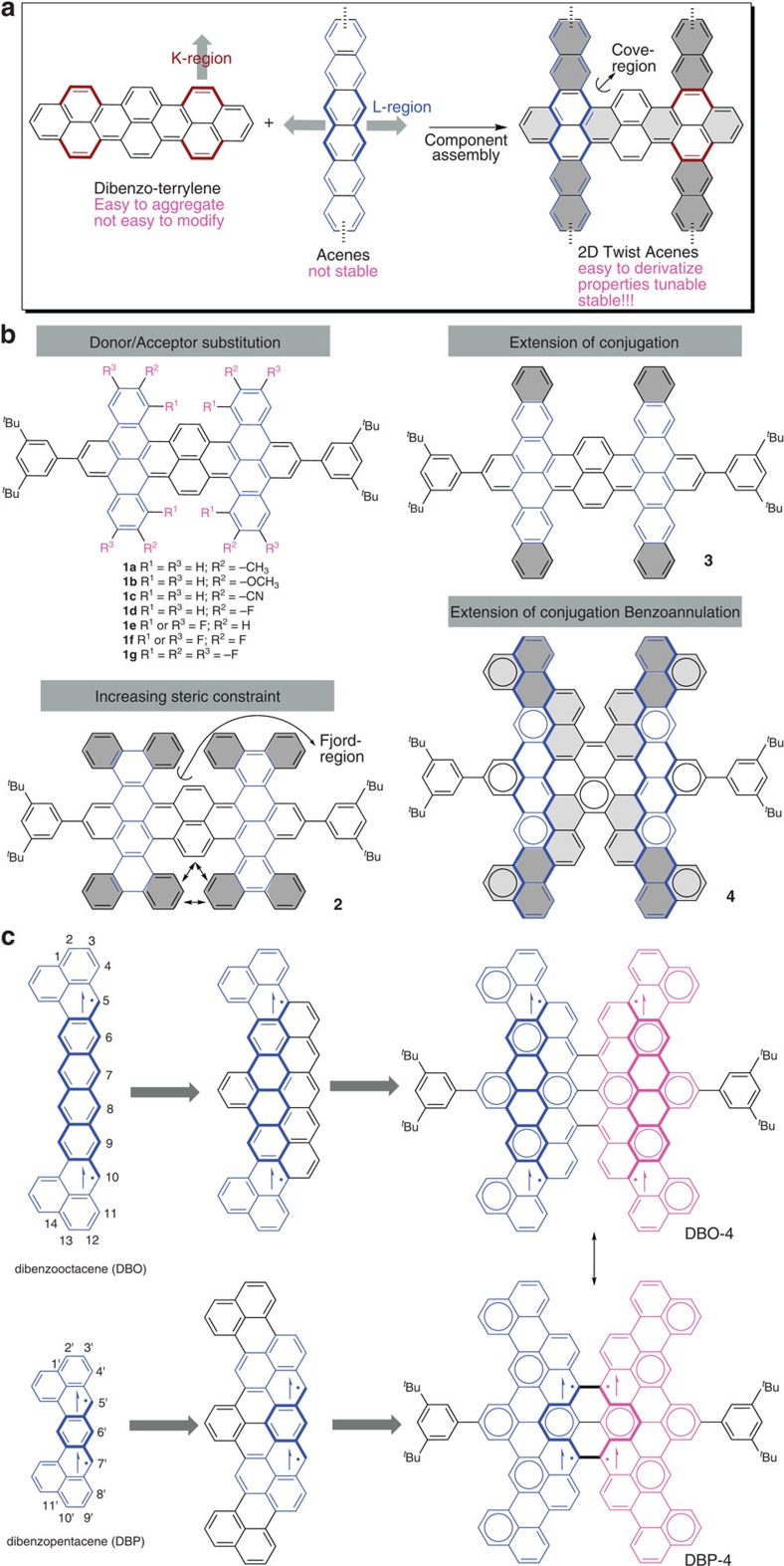
Conceptual approaches toward twisted 2D acenes. (**a**) π-Components of 2D acenes. (**b**) Molecular structures of 2D acenes with increasing steric constraint (**2**), extension of conjugation (**3**) and with both extension of conjugation and benzoannulation at the L-region (zigzag edge; **4**). (**c**) Chemical structural evolution of **4**: (top) Benzoannulation of octacene to generate phenalenyl radicals annulated with a central tetracene and further pentacene-annulation on the other side to provide more stable diradicals, two of which connect in the central area; (bottom) evolution from a dibenzopentacene with benzo- and naphtheno-annulation, with the reactive 5',7'-positions of the pentacene connected and protected together.

Figure 1a showed the conceptual approaches toward twisted 2D acenes. We positioned electron-donating/withdrawing groups (for example, CH_3_, OMe, CN, F) to alter the optical absorbance and the energy levels of the highest occupied molecular orbital (HOMO) and lowest unoccupied molecular orbital (LUMO) of the tetracene-dibenzoterrylene hybrid, in addition to tuning the molecular packing in the solid state (Fig. 1b). We introduced phenanthrenyl groups to further increase the steric constraint, and naphthyl and perylenyl groups to elongate the acenes and allow annulation of benzene rings. Using this approach, 2D twisted acenes varying from closed-shell to open-shell structures were available through extension of the acenes vertical to the benzoterrylene and benzoannulation at the zigzag edge. Compound **4** can be considered (Fig. 1c) as a hybrid of two pentacene-annulated-tribenzo-octacenes, in which two phenalenyl radicals (5,10 positions of octacene) are annulated with a central tetracene unit, or having evolved from a dibenzopentacene with the reactive 5′,7′-positions of the pentacene connected and protected together. In both cases, one more Clar sextet was formed on resonance to tetraradical state, which also predicted the stability of the tetraradical.

### Synthesis of target compounds

**Figure 2:**
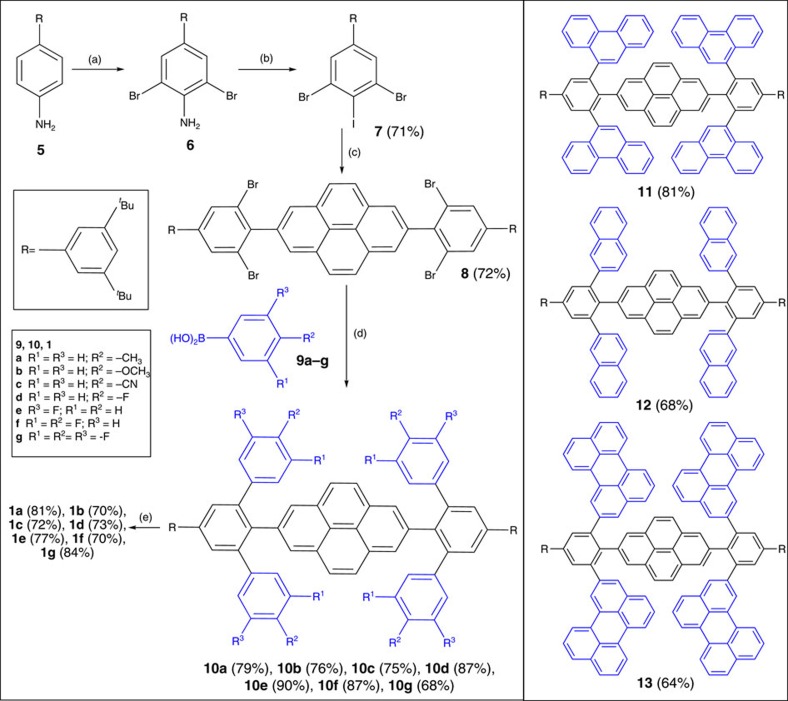
Synthesis of the tetracene-dibenzoterrylene hybrids **1a**–**f** and the precursors 11–13 for generation of **2**–**4**. (a) Br_2_, DCM/MeOH; (b) NaNO_2_, AcOH/H_2_SO_4_, KI/I_2_; (c) 2,7-bis(4,4,5,5-tetramethyl-1,3,2-dioxaborolan-2-yl)pyrene, Pd(PPh_3_)_4_, Na_2_CO_3_, THF/H_2_O; (d) Pd(PPh_3_)_4_, Na_2_CO_3_, toluene/EtOH/H_2_O; (e) FeCl_3_, CH_3_NO_2_, CH_2_Cl_2_, 4 h.

Figure 2 displays the synthesis of **1**, starting from the bromination of the amine **5** (ref. 45) with Br_2_ followed by conversion of the amino group to the iodide through diazotization. Compound **7** was coupled with 2,7-bis(4,4,5,5-tetramethyl-1,3,2-dioxaborolan-2-yl)pyrene through Suzuki–Miyaura cross-coupling to give the key intermediate **8**. This compounds was coupled with a series of RB(OH)_2_ derivatives **9** to provide the precursors **10a**–**g** for Scholl-type oxidative cyclodehydrogenation, in which FeCl_3_ was used as both a Lewis acid and an oxidant. *tert*-Butyl groups were introduced to increase the solubility of the final product. A phenanthrenyl group was used to further increase the steric constraint, leading to precursor **11**; naphthyl and perylenyl groups were introduced to elongate the acenes and introduce the annulation benzene rings, providing the precursors **12** and **13**, respectively.

For **10e**, C–C bonds were formed *ortho* and *para* to the *meta* fluorine atom, leading to a mixture where the fluorine atoms were located at the R_1_ and R_3_ positions, as supported in the X-ray structure (see below). For **11**, the Scholl reaction at the K-region (convex armchair edge) of the phenanthrenyl group led to **2** in 57%, which is too crowded at the fjord regions to allow two benzene rings to get sufficiently close to form a further C–C bond^46^. π-Conjugation extended compound **3** was obtained from **12** in 78% yield. The oxidative cyclohydrogenation of **13** is time-dependent. When the reaction time was <2 h, we obtained some incompletely cyclized products, which were difficult to separate from the target **4**; when the reaction time was longer than 12 h we obtained some byproducts with similar molecular weight as **4** but with different absorption spectra. The black compound **4** is well-soluble in C_2_H_2_Cl_4_ and *o*-dichlorobenzene (dark-green solution), partially soluble in CH_2_Cl_2_ and CHCl_3_, we could purify it (in 51% yield) through column chromatography on deactivated silica gel with CH_2_Cl_2_/MeOH (5/1). We characterized all of the intermediates and final products, except for **4**, using ^1^H and ^13^C nuclear magnetic resonance (NMR) spectroscopy and high-resolution mass spectrometry (MS); we characterized compound **4** only through high-performance liquid chromatography, high-resolution MS and elemental analysis.

### Conformations characterization of compounds 1–3

**Figure 3:**
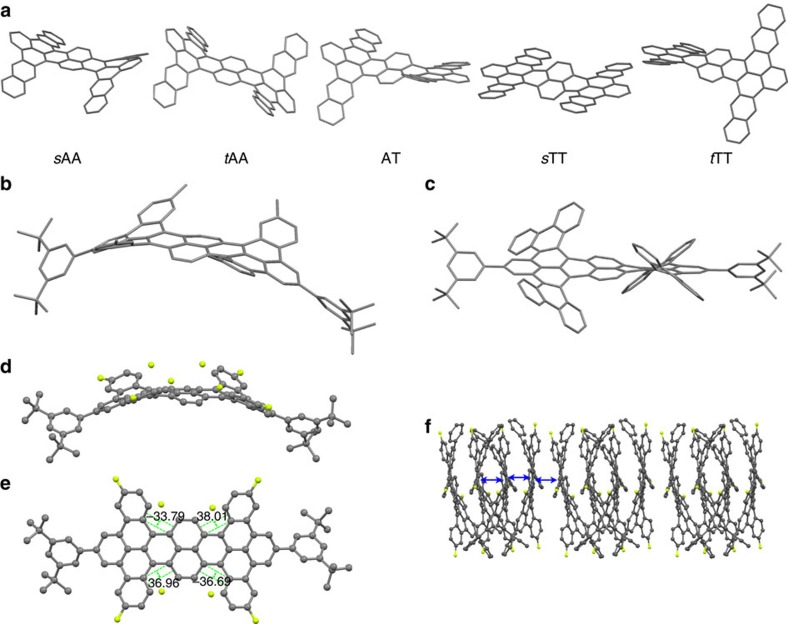
Conformational flexibility of the 2D acenes. (**a**) Possible molecular conformations of **3** and (**b**,**c**) the most stable conformations of (**b**) **1a** and (**c**) **2**, calculated at the B3LYP/6-31G (**d**) level of theory. *c*AA-: anti-folded and anti-folded in the *cis* direction; *t*AA-: anti-folded and anti-folded in the *trans* direction; AT-: anti-folded and twisted; *c*TT-: twisted and twisted in the *cis* direction; *t*TT-: twisted and twisted in the *trans* direction. (**d**) Side and (**e**) top views of the π-backbone of **1e** from crystal structure analysis; (**f**) molecular packing of **1e**, viewed along the *c*-axis of the unit cell; the hydrogen atoms and *tert*-butyl groups have been omitted for clarity; carbon and fluorine atoms are coloured grey and green, respectively.

At first, we performed density functional theory (DFT) optimization to gain insight into the molecular conformations of these 2D acenes. The nonplanar conformations of these compounds arose from severe steric strain between the two C–H bonds at the cove or fjord regions, as can be seen clearly in the model of **3** in Fig. 3a. DFT calculations revealed that **3** has five possible conformations (Fig. 3a); the hexacene units can take on conformations that are twisted or anti-folded with the central naphthalene unit, while the outer naphthalene rings in the hexacenes can be contorted out of the plane defined by the dibenzoterrylene core in the *cis* or *trans* direction: *c*AA-**3**, *t*AA-**3**, AT-**3**, *c*TT-**3** and *t*TT-**3**. Compound **1** existed in conformations analogous to those of **3** (Supplementary Fig. 69; Fig. 3b displays the most stable conformation of **1a**). In all of these cases, the C_2_-symmetric *c*AA structures were calculated to be more stable than the other conformers by 3.03–24.65 kJ mol^−1^ (Supplementary Table 1).

We grew single crystals through diffusion of acetone into a solution of **1e** in CHCl_3_ (Supplementary Table 2). Figure 3d displays the crystal structure of **1e**. The fluorinated outer aromatic rings are contorted, in the same direction, out of the plane defined by the dibenzoterrylene core, resulting in a saddle-like conformation, consistent with the calculated gas-phase structure (*c*AA-**1e**). The degree of twisting (*θ*), measured as the dihedral angle between the central naphthalene and the fluorinated outer benzene/terminal benzo group, were in the range of 32.2–37.3° (Fig. 3e). Molecules of **1e** were stacked in columns along the *c*-axis (Fig. 3f). Within a columnar stack, two saddle molecules with curvature in the same direction formed a π-dimer with effective π–π surface overlap; this π-dimer then stacked with another π-dimer of opposite molecular orientation.

For compound **2**, we predicted a similar set of five possible conformations (Supplementary Fig. 70), with *c*TT-**2** calculated as the most stable because of additional steric strain between the phenanthrenyl moieties (Fig. 3c). Fortunately, this zero-dipole-moment conformer *c*TT-**2** could be separated through column chromatography. Through slow diffusion of acetone into a solution of *c*TT-**2** in CHCl_3_, were obtained crystals for X-ray analysis, although they diffracted only weakly (Supplementary Fig. 71). A partial solution revealed that the phenanthrenyl units were twisted with respect to the central naphthalene unit in the *cis* direction. Although frozen in a single conformation as a result of crystal packing constraints in the solid state, these 2D acenes are highly flexible in solution and change their shape constantly on heating (Supplementary Fig. 72).

### Optical and electrochemical properties of compounds 1–3

**Figure 4:**
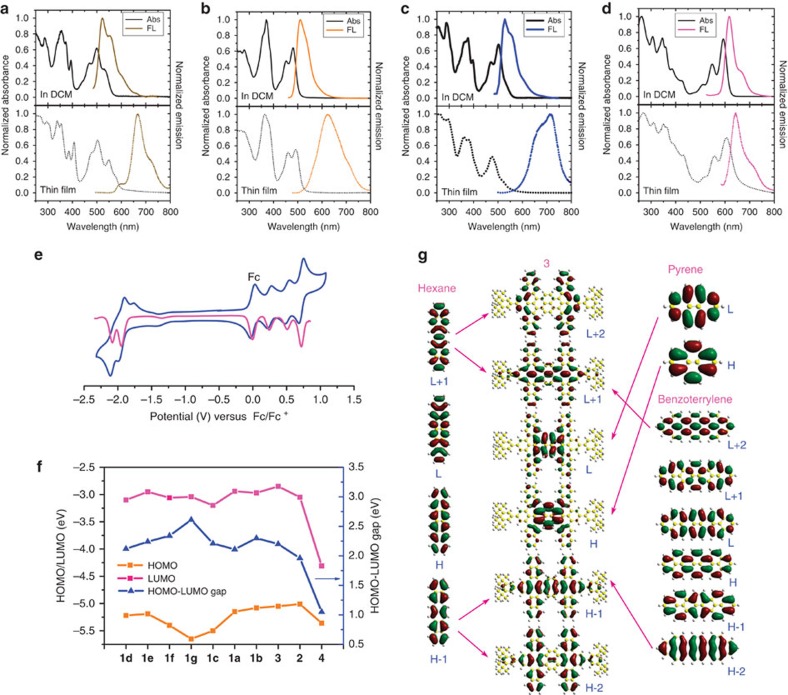
Optical, electrochemical behaviour and orbital analysis of compounds **1**–**3**. Absorbance (black) and emission (coloured) spectra of (**a**) **1a**, (**b**) **1g**, (**c**) **1c** and (**d**) **2** recorded in CH_2_Cl_2_ solution (solid lines) and in the solid state (spin-cast from CH_2_Cl_2_ solutions; dotted lines). (**e**) CV (blue) and DPV(red) traces of **3** in CH_2_Cl_2_ (0.1 mM) at room temperature. Scan rate: 100 mV s^−1^; working electrode: glassy carbon; reference electrode: Ag wire; electrolyte: TBAPF_6_. Fc/Fc^+^: ferrocene/ferrocenium. (**f**) HOMO and LUMO energies and band gaps of **1**–**4**. (**g**) Frontier orbitals analysis of **3** calculated at the B3LYP/6-31G (**d**) level.

We measured the ultraviolet–visible absorption spectra of compounds **1**–**3** in CH_2_Cl_2_ solution (Fig. 4a–d, Supplementary Fig. 73, Supplementary Table 3). Absorption bands appeared in the ranges 300–400 nm and 400–600 nm with molar absorptivities of >50,000 M^−1^. The substituents and lengths of the acenes appeared to influence the π-stacking of these 2D acenes. For **1**, three peaks in the range 400–600 nm, attributed to 0→0, 0→1 and 0→2 vibrational absorptions, were good handles for observation of its aggregation. With the increasing of concentration, the intensity of the 0→0 absorption decreased; in particular, the 0→0 absorption became a shoulder for the CN-substituted **1c**, even disappearing for the trifluorine-substituted **1g**. These results suggested the permanent formation of H-aggregated π-dimers, as in the crystal structure of **1e** (Fig. 3d) and the molecular modelling of **1g** (Fig. 3g), in solution. On increasing the concentration, the intensity of the 0→0 vibrational absorption decreased further (Supplementary Fig. 74), with the 0→2 vibrational absorptions becoming the main absorption bands in the spectra of spin-coated thin film of **1c–f**, consistent with further H-aggregation of their π-dimers. In the thin films of **1a** and **1b**, in addition to H-aggregation we also observed some J-aggregates, as indicated by the red-shifting of new bands. Such J-aggregates were also found for compounds **2** and **3** with bulky acenes, with red-shifting of the absorption maxima (*λ*_max_).

The photoluminescence features of these compounds in dilute solution (10^−3 ^mmol l^−1^) were complementary of their absorption spectra; quantum yields were in the range 0.52–0.89 (Supplementary Table 3). We observed large bathochromic emission features in the emission spectra of these compounds on concentrating or going from solution to the solid state, consistent with strong aggregation. For the tetracene-dibenzoterrylene **1**, we observed a bathochromic emission shift from 108 to 190 nm; the more bulky compounds **2** and **3** featured shifts of only 24 and 71 nm, respectively.

We used cyclic voltammetry (CV) and differential pulse voltammetry to examine the electrochemical behaviour of **1**–**3** in CH_2_Cl_2_ containing 0.1 M TBAPF_6_ as the supporting electrolyte (Supplementary Fig. 75, Supplementary Table 3). The CV traces of **1a**–**c**, **1e**, **1f** and **2** each featured two well-defined, reversible oxidation potentials. The cyclic voltammogram of **3** was more remarkable, displaying three oxidation potentials for four electrons at +0.25 V (**1e**), +0.51 V (**1e**) and +0.72 V (**2e**), all as fully reversible waves (Fig. 4e). Compound **1d** exhibited a similar oxidation behaviour, with signals at +0.42 V (**1e**), +0.63 V (**1e**) and +0.87 V (**2e**) (versus Fc/Fc^+^), implying multi-charge storage ability^47^ for both **1d** and **3**. Compared with **1a**, the electron-donating MeO groups decreased the oxidation potentials by 70 mV, while the electron-withdrawing groups increase the oxidation potentials more evidently; for example, CN groups shifted the oxidation potentials by ∼350 mV. The oxidation peaks shifted to higher potentials on increasing the number of fluorine atoms (50–70, 250 and 500 mV for one, two and three fluorine substituents, respectively). Extension of the conjugation of the acene units also decreased the oxidation potentials; for example, to 0.25 V for **3** with its six conjugated benzene rings and to 0.21 V for **2** with its eight conjugated benzene rings. Consistent with these findings, **2** and **3** appear to be susceptible to slow oxidative degradation in solution; no such degradation appeared in the solutions of the other compounds. All of these compounds exhibited two reduction potentials, with the effects of the substituents on the reduction potentials occurring in the same direction as those on the oxidation potentials, but to a lesser extent. Accordingly, the band gaps of these species were influenced by the substituents and the conjugation length, with electron acceptors decreasing the HOMO energy level and, thereby, increasing the band gap (see Fig. 4f, especially for the trifluorinated derivative).

We performed the DFT calculations at the B3LYP/6-31G(d) level to examine the electronic structures of the dibenzoterrylene-acene hybrids. The calculated frontier orbitals indicated that they maintained the characteristics of their individual components. The HOMO and LUMO orbitals of **1a** are mainly localized in the central pyrene unit (Supplementary Table 4). We observed nodal planes perpendicular to the molecular skeleton in the HOMOs and LUMOs of these compounds, with the electron densities distributed symmetrically on the two sides of the nodal plane. The HOMO-1 and LUMO+1 orbitals of **1a** are comparable with the HOMO-2 and LUMO+2 orbitals, respectively, of dibenzoterrylene. The other compounds had similarly appearing orbitals, except for **1f**, where the strongly electron-withdrawing CN group affected the polarization of its orbitals (Supplementary Table 4). Interestingly, extension of the acenes vertical to the benzoterrylene, as in **3**, led to more independent characteristics for the pyrene, benzoterrylene and acene subcomponents (Fig. 4g). Increasing the steric constraint of these acenes led to compounds exhibiting the properties of the central benzoterrylene (for example, for **2** in Supplementary Fig. 76). Time-dependent DFT calculations revealed that the absorption behaviour of these compounds was the result of a mixture of pyrene-, dibenzoterrylne- and acene-like orbitals (Supplementary Fig. 77, Supplementary Tables 5–11). For **1**, the main band in the range 400–600 nm, corresponding to the HOMO–LUMO transition, according to the calculation, was caused by a transition from a pyrene-like HOMO to a pyrene-like LUMO. The second band, near 350 nm, due to the HOMO−1→LUMO+1 transition, was related to the transition between the dibenzoterrylene-like HOMO-2 and LUMO+2. The small sharp peak near 400 nm arose from the transition from a pyrene-like HOMO to a dibenzoterrylene-like LUMO+2. We observed similar transition behaviour for **3**, although the band in the 300–400 nm region was red-shifted by ∼50 nm. Consistent with the molecular orbital profiles, **2** displayed a dibenzoterrylene-like HOMO→LUMO transition for the main band near 600 nm, with a transition between the dibenzoterrylene-like HOMO-2 and LUMO+2 for the band near 350 nm. We also observed dibenzoterrylene-like HOMO-2→LUMO and HOMO→LUMO+2 transitions for the weak band at 344 nm.

### Optical and magnetic properties of open-shell compound 4

**Figure 5:**
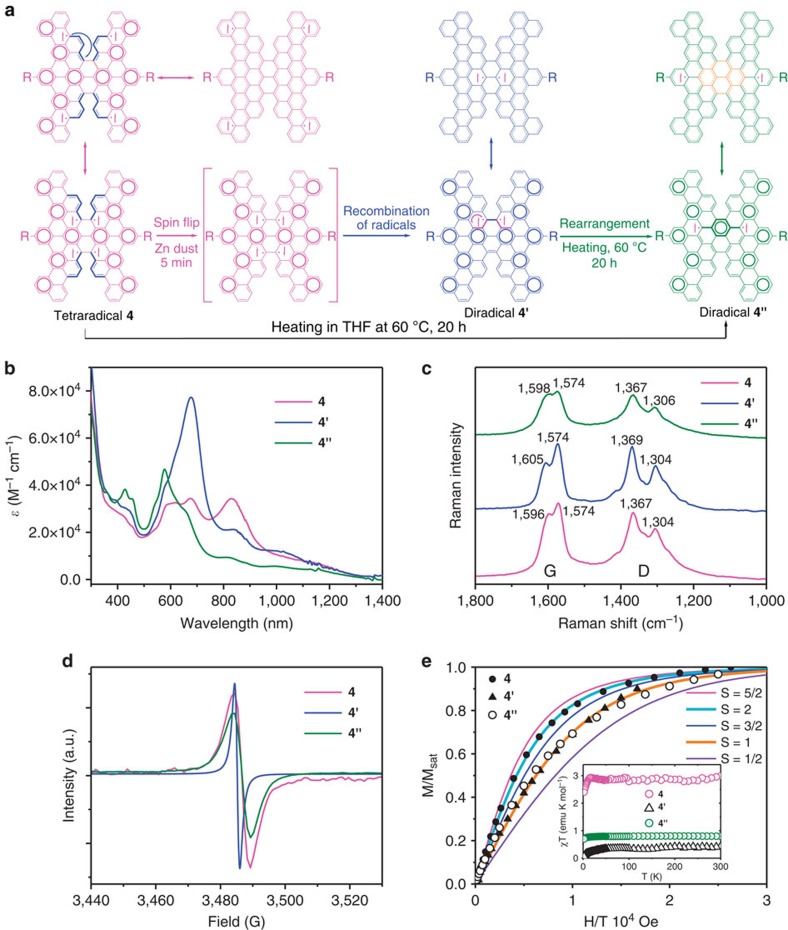
Spectroscopic characterization of compound **4**. (**a**) Transformation of tetraradical **4** to diradicals **4**' or **4**'' mediated with spin catalysis by Zinc or trace radicals in THF, and the resonance structures of **4**, **4**' and **4**''. (**b**) UV–vis-near-infrared spectra of **4**, **4**' and **4**'' in CH_2_Cl_2_. (**c**) Raman spectra of solid **4**, **4**' and **4**''. (**d**) ESR spectra of chloroform solutions of **4**, **4**' and **4**'' recorded at room temperature. (**e**) The magnetization of **4**, **4**' and **4**'' are plotted as M/M_sa_t versus H/T, with Brillouin functions for S=5/2, 2, 3/2,1 and 1/2. The fitting parameters for **4**, **4**' and **4**'' are *S*=2.08 (1.9 K), 1.02 (3 K), 1.14 (1.9 K) and the corresponding *M*_sat_=0.8, 0.37, 0.88 μB, respectively. Inset plots: the magnetic susceptibility of **4** (2,000 Oe), **4**' (10,000 Oe) and **4**'' (2,000 Oe) are plotted as *χ*T versus T.

Dark-green compound **4** provides an absorption spectrum (Fig. 5b) displaying bands with maxima at 834, 675 and 585 nm with a tail into the near-infrared region (ca. 1,200 nm). We calculated an optically measured band gap of ∼1.03 eV. The CV trace of **4** (Supplementary Fig. 75) in 1,2-dichlorobenzene (0.1 M tetrabutylammonium hexafluorophosphate) displayed a one-step irreversible oxidation and a one-step reduction. The HOMO and LUMO energies, calculated through CV, were −5.31 and −4.26 eV, respectively, corresponding to a band gap of 1.05 eV, very close to that determined *in silico* (1.1 eV). In addition, we observed almost no fluorescence from **4** in solution and in the solid state.

Raman spectroscopy is a very useful tool for characterizing benzene-type aromatic radicals^48^, because characteristic benzene vibrational Raman bands exist near 1,600 cm^−1^ that are very sensitive to the electronic configuration within the six-membered benzene ring, either ‘benzoquinoidal' or ‘benzoaromatic.' The Raman spectrum of **4** featured two sets of peaks, much like the D and G bands of graphene (Fig. 5c). The peak near 1,367 cm^−1^ corresponds to the breathing vibrations of sp^2^-hybridized carbon domains in aromatic rings (that is, the D band); we assign the peak near 1,596 cm^−1^ to the first-order scattering of the E_2g_ mode observed for in-phase stretching vibration of sp^2^-hybridized carbon domains in aromatic rings (that is, the G band)^49^. The downfield-shifting of the D and G bands (from 1,367 to 1,304 cm^−1^ and from 1,596 to 1,574 cm^−1^, respectively) indicated the coexistence of ‘benzoquinoidal' and ‘benzoaromatic' benzene rings.

The NMR spectrum of **4** in C_2_D_2_Cl_4_ did not feature any signals at room temperature, nor after cooling to −50 °C, suggesting the presence of a considerable paramagnetic species. In addition, the solution of **4** provided a featureless broad ESR signal (Fig. 5d), resulting from the long-distance spin−spin dipole interaction within the molecules and the extended spin-delocalization^48^,^50^,^51^. These data indicate that **4** exists as an open-shell multi-radical in the ground state.

We performed SQUID measurements for **4** in the powder form as a function of magnetic field (*H*=0−5 × 10^4^ Oe and *T*=1.9 K) and temperature (*T*=2−300 K at *H*=2,000 Oe). The value of S=2.08, determined from the curvature of the Brillouin plots, indicate the quintet (S=2) ground state for tetraradical **4** (Fig. 5d). The inset of Fig. 5d presents a plot of χ_M_T(T) for **4**. The constant value of χT (2.84 emu K mol^−1^), as evidenced by the flatness of the χT versus T plots in the T=30−300 K range, indicates that there is no significant change in the thermal population of spin states up to 300 K. After removing the temperature independent contribution, the numerical fit of the data by the Currie-Weiss model give Currie constant of C=2.84 emu K mol^−1^ and Weiss constant *θ*_W_ of −0.38 K (Supplementary Fig. 78), which indicated weak intermolecular antiferromagnetic interaction below 20 K.

Interestingly, solid **4** displayed high stability, and there was no obvious decomposition when the solid was stored under ambient air and light conditions for months. However, mixing the tetraradical **4** with Zinc dust can generate another radical **4**′, and heating the solution of **4** or **4**′ in THF at 60 °C for about 20 h will transform both of them to another species **4**′′ completely**. 4**′ and **4**′′ showed no molecular weight change, while the strongest absorption bands of **4**′ and **4**′′ were shifted to 678 and 575 nm, respectively, with the absorption band at 834 nm became weak (Fig. 5b and Supplementary Fig. 81). Similar Raman spectra (Fig. 5c) and magnetic field dependent magnetic signal changes (Fig. 5e) indicate unequivocally that both of **4**′ and **4**′′ are triplet biradicals at ground state (Supplementary Figs 79 and 80). The values of χT≈1 emu K mol^−1^ were found for **4**′ and **4**′′, after values of χT (0.77, 0.36 emu K mol^−1^) are corrected by spin concentration (*M*_sat_=0.88, 0.37 μB). The transformation from tetraradical to diradicals is probably due to the fact that one pair of electrons in the tetraradical formed one covalent bond by Zinc surface catalysed spin flip^52^,^53^ (Fig. 5a), the left two electrons delocalized on the two pentacene-annulated-tribenzo-octacene units, respectively, with parallel spin electron patterns at ground state. The obtained diradical **4**′ can be rearranged to a more stable diradical **4**′′ on heating. Transformation directly from **4** to **4**′′ in hot THF processed through spin catalysis^52^,^53^ by the trace radicals in THF, followed by the thermal rearrangement, without the observation of the intermediates. Sharpening ESR signal for **4**′ (Fig. 5d), indicates that the spin−spin dipole interaction distance within the molecules of **4**′ is shorter than that of **4** and **4**′′. The transformation processes and the possible resonance structures of **4**, **4**′ and **4**′′ are summarized in Fig. 5a, with the supports from the following DFT optimization of the tetraradical and diradicals. Tetraradical **4** can be considered as four phenalenyl radicals annulated by twisted H-shaped acene; intermediate diradical **4**′ featured two close-contacted radical pairs like two over-lapped phenalenyl radicals; **4**′′ can be viewed as two phenalenyl radicals fused by central pyrene, with benzene rings surrounded. The detailed spin catalysis mediated transformation mechanism needs further investigation.

**Figure 6:**
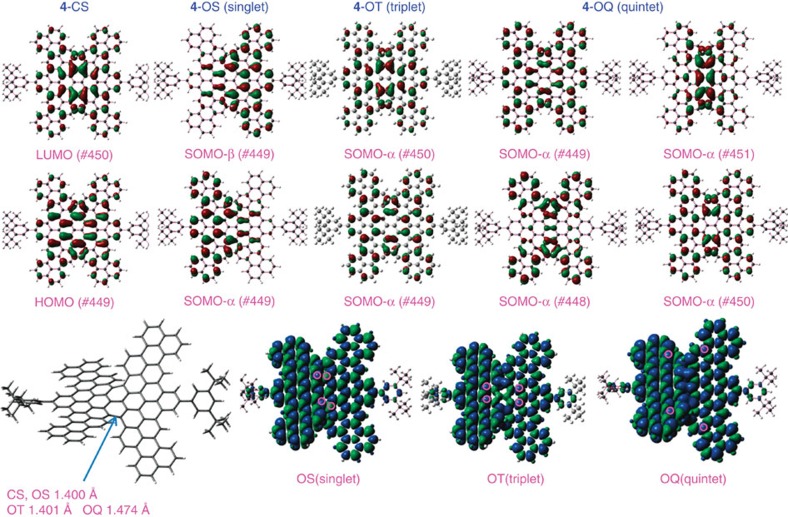
Orbitals and spin-density distributions for closed-shell and open-shell compound **4**. Calculated (UCAM-B3LYP) HOMOs and LUMOs for **4**-CS, SOMOs and spin-density distributions for **4** in the singlet (**4**-OS, S=0), triplet (**4**-OT, S=1) and quintet state (**4**-OQ, S=2). Blue and green surfaces represent α- and β-spin densities, respectively. The red marks indicate the atoms exhibiting highest spin-density. The central bond connecting the two pentacene-annulated-tribenzo-octacene units in quintet-state pocesses the single-bond charateristic, which indicates the less conjugation between the two units than other states.

Although 1D PAHs (n-acenes) featured the antiferromagnetic ground state due to the zigzag-shaped boundaries, which cause π-electrons to localize and form spin orders at the edges, the longer acene ground states are polyradical in nature^54^,^55^. 2D PAHs (periacenes and circumacenes) develops a strong multiradical character with increasing zigzag chain length^56^,^57^. Our experimental results indicated tetraradical character for the twisted 2D molecule **4**. We performed DFT calculations at the UCAM-B3LYP level of theory to investigate the ground state of the tetraradical **4** (Fig. 6). Because of steric effects, the molecule adopted a twisted planar structure. The structure **4**-CS features extended π-electron delocalization from the pyrene core to the four annulated perylene units, with the central pyrene unit possessing the largest HOMO and LUMO coefficients (Fig. 6), suggesting that it would be the most reactive site. For the broken-symmetry singlet state **4**-OS, we observed disjointed singly occupied molecular orbitals (SOMOs), SOMO-α and SOMO-β, with orbital coefficients mainly localized at the terminal pentacene-annulated-tribenzo-octacene units. The spin-density distribution reveals delocalization with the central pyrene units having the largest density, especially the K-region carbon atoms (also the 5′, 7′ positions of dibenzopentacene) (Supplementary Fig. 82, Supplementary Tables 12–14); this situation is different from that found in bisphenalenyls, where the spin-density is delocalized mainly at the terminal phenalenyl units^25^, with antiparallel spin electron patterns typical for this broken-symmetry singlet state. This S=0 state can be considered as the **DBP-4** form in Fig. 1c, which also corresponds to an S=1 state when the spin electrons parallel to each other (diradical **4′** in Fig. 5a). For the high-spin quintet state (**4**-OQ, S=2), we observed a large spin-density at the 5,10 positions of the octacene core—positions that are blocked by the annulated pentacene, which is the **DBO-4** form (Fig. 1c, also the tetraradical **4**). However, for the S=1 state (**4**-OT, also the diradical **4′′** in Fig. 5a), the spins are delocalized on the pentacene-annulated-tribenzo-octacene units, with more distribution on the 7, 8 positions of the octacene core, well-protected by surrounding benzene rings. From the SOMO-α and SOMO-β profiles and the spin-distributions of the singlet **4**-OS and high-spin triplet **4**-OT and quintet **4**-OQ, we ascribe the good chemical stability of the tetraradical toward oxidation and dimerization/oligomerization to its thermodynamic stabilization^33^, resulting from both delocalization and kinetic blocking through benzoannulation.

## Discussion

We have developed an efficient method for the preparation of a series of stable 2D twisted dibenzoterrylene-acenes. We have investigated their electronic structures and geometries in the ground state using various experimental techniques, assisted by DFT calculations. The photophysical, electrochemical, and magnetic properties of these compounds were dependent on the length of the acene. The ground-state structures in this series of molecules were tunable, with **1**–**3** being closed-shell hydrocarbons and **4** being an open-shell singlet tetraradical. Compound **4** is the first stable twisted 2D hydrocarbon tetraradical obtained by hybridizing dibenzoterrylene with acene, which can be transferred to more thermodynamic stable triplet diradical. The high stability of the tetraradical and diradical arose from thermodynamic stabilization, due to delocalization and kinetic blocking through benzoanullation. We believe that 2D twisted conjugated molecules with various width and edge structures could also be synthesized in a similar approach. With the installation of different functional groups such as heterocycles on the periphery of such 2D twisted conjugated molecules, specific functionalities on them could be obtained. Combination this approach with polymerization could give great promise for the synthesis of chemically precise, multiple-dimensional polymers. Moreover, the unique optical, electronic, and magnetic properties of these extended 2D acenes suggest that they might be promising candidates for use in nonlinear optics, field effect transistors, and organic spintronics.

## Methods

### Materials

Most of the chemical reagents were purchased from Alfa Aesar or Aldrich Chemicals and were utilized as received unless indicated otherwise. All solvents were purified using standard procedures. Column chromatography was performed on silica gel (size 200–300 mesh).

### Synthesis procedures of compounds 1–4

For details of the synthetic procedures, see Supplementary Methods. For NMR and high-resolution mass spectra of compounds in this manuscript, see Supplementary Figs 1–68.

### Sample characterization

^1^H and ^13^C NMR spectra were recorded on a Bruker AVANCE 400 or Bruker AVANCE III 500WB instrument, at a constant temperature of 25 °C. Chemical shifts are reported in parts per million from low-to-high field and referenced to TMS. Matrix-assisted laser desorption/ionization Fourier transform ion cyclotron resonance (MALDI-FT-ICR-MS) MS were performed on a Bruker Solarix 9.4T FT-ICR-MS mass spectrometer. EI mass spectrometric measurements were performed on a SHIMADZU GCMS-QP2010 puls Spectrometer. Elemental analyses were recorded on a Carlo-Erba-1106 instrument. Electronic absorption spectra were measured on a JASCO V-579 spectrophotometer. Raman spectra were taken on a NT-MDT NTEGRA spectra Raman spectroscopy SPM system. CV experiments were performed using an electrochemical analyser with a conventional three-electrode configuration, glassy carbon electrode as a working electrode and Ag/AgCl as a reference electrode. All experiments were performed in CH_2_Cl_2_ with 0.1 M of *n*Bu_4_NPF_6_ as a supporting electrolyte, the ferrocene/ferrocenium ion (Fc/Fc^+^) as inter-reference. The E_1/2_ values were determined as 1/2(E_pa_+E_pc_), where E_pa_ and E_pc_ are the anodic and cathodic peak potentials, respectively.

### X-ray diffraction data analysis

Crystals of **1e** and **2** were grown by slow diffusing acetone to the solutions in CHCl_3_ solution. Single crystal X-ray diffraction data were collected on a Agilent SuperNova (Dual, Cu at zero, Atlas) diffractometer with Cu Kα radiation (*λ*=1.54184 Å) from micro-focus sealed X-ray tube. Intensities were corrected for absorption effects using the multi-scan technique^58^. The structures were solved by direct methods and refined by a full matrix least squares technique based on F^2^ using SHELXT program^59^. The refinement details: Refinement of F^2^ against ALL reflections. The weighted R-factor wR and goodness of fit S are based on F^2^, conventional R-factors R are based on F, with F set to zero for negative F^2^. The threshold expression of F^2^>2sigma (F^2^) is used only for calculating R-factors (gt) and so on, and is not relevant to the choice of reflections for refinement. R-factors based on F^2^ are statistically about twice as large as those based on F, and R- factors based on ALL data will be even larger. For X-ray data see Supplementary Data 1 and 2.

Attempts to refine peaks of residual electron density as solvents led to 12 CHCl_3_ and 6 acetone and some additional peaks of residual electron density which can't be refined successfully. The data were corrected for disordered electron density through use of the SQUEEZE procedure^60^ as implemented in PLATON^61^. A total solvent-accessible void volume of 2,179.1 A^3^ with a total electron count of 584.3 was found in the unit cell in four voids.

The structure solution of crystal **2** (Cell: 19.2306(4) Å, 19.4633(5) Å, 58.6850(14) Å, 90°, orthorhombic P c c n, *Z*=8) shows two independent half molecules, one of which is disordered with its symmetry equivalent. It is possible that the cell chosen for the current refinement in represents a subcell and that reflections of a larger supercell were too weak to be observed. The current result represents a preliminary connectivity only. We have grown the crystals of this structure several times, the crystals are thin plate, and we tried to collect better data, however, the data reported here is the best we can obtain. The six-membered rings in the phenanthrenyl groups and terminal benzenes with *t*-butyl groups were flattened with AFIX 66 and DFIX. As agreed by the reviewer, although the data is poor, it is perfectly fine for the assumptions that there is a twist associated with the molecule as predicted by the DFT calculation. We present with caution some geometrical parameters: phenanthrenyl units twisted with the central naphthene unit in the *cis* direction.

ESR spectra were obtained with a Bruker E500-10/12 spectrometer. No additional hyperfine coupling was detected at 0.1 G, thus experiments were performed at 1.0 or 2 G for improved signal intensities. The sweep width was 83.89 s, time constant was 40.96 ms and the microwave power was 10.1 mW (corresponding to attenuation=13 dB, which was sufficiently high to avoid power saturation).

Magnetic measurements were performed on a Quantum Design MPMS-XL5 SQUID magnetometer. Small amounts of sample material (between 30 and 40 mg) were put into KLF containers and brought into measuring position using a straw. The variation of the magnetization as a function of temperature at fixed external magnetic field (*T*=2–300 K at *H*=2,000 Oe) and as a function of the external field at fixed temperature (*H* =0–5 × 10^4^ Oe and *T*=1.9 K) was studied. The powder of **4** was sealed in a plastic tube. The signal of sample holder and plastic tube was deducted by measuring the sample holder and plastic tube under same conditions. A correction for the diamagnetism of the sample and the sample container was applied before calculating the susceptibilities from the magnetization data. For all other samples of solid di- and tetraradicals, the correction for diamagnetism was based on high-temperature extrapolation of the *χ* versus 1/*T* plots, that is, a suitable numerical factor (*M*_dia_) was added to the magnetization (*M*), until the *χT* versus *T* plot becomes flat in the high-temperature range.

### Theoretical calculations of target compounds **1**–**4**

For compound **1**, **2** and **3**, DFT calculations were performed using the Gaussian 03 program^62^, Geometries were optimized in the gas-phase using the B3LYP functional and 6–31 g (d) basis set on all atoms. Theoretical calculations of compound **4** were carried out by using the Gaussian 09 suite of programs^63^. The initial geometry optimization of **4** was performed with the UCAM-B3LYP level of theory on the singlet state and the Handy and co-workers'^66^ long-range corrected version of B3LYP 6–31G*, and all electron basis sets were used for all atoms^64^,^65^,^66^. The resulting DFT solution (singlet ‘closed-shell': zero-spin-density on all atoms) was further tested for its stability with the STABLE=OPT keyword^67^. A spin symmetry broken DFT solution was found with lower energy. Then the Guess=Read keyword was used to perform the optimization at the UCAM-B3LYP level (singlet open-shell). For DFT data see Supplementary Data 3–6.

### Data availability

Data referenced in this study are available in ‘Cambridge Crystallographic Data Centre (CCDC)' with the accession codes ‘1457979' and ‘1457980', see Supplementary Data 1 and 2. All other data supporting the findings of this study are available within the article and its Supplementary Information files.

## Additional information

**How to cite this article:** Li, Y. *et al*. A method for controlling the synthesis of stable twisted two-dimensional conjugated molecules. *Nat. Commun.* 7:11637 doi: 10.1038/ncomms11637 (2016).

## Supplementary Material

Supplementary Information Supplementary Figures 1-82, Supplementary Tables 1-14, Supplementary Discussion and Supplementary Methods

Supplementary Data 1 Data of X-ray crystallographic structures for compound **1e**.

Supplementary Data 2 Data of X-ray crystallographic structures for compound **2**.

Supplementary Data 3 Cartesian coordinates of DFT computed geometries of close shell compound **4**.

Supplementary Data 4 Cartesian coordinates of DFT computed geometries of open shell singlet compound **4**.

Supplementary Data 5 Cartesian coordinates of DFT computed geometries of open shell triplet compound **4**.

Supplementary Data 6 Cartesian coordinates of DFT computed geometries of open shell quintet compound **4**.
