# Supplementary material for: A method for controlling the synthesis of stable twisted two-dimensional conjugated molecules
Source: Nat Commun. 2016 May 16;7:11637. doi: 10.1038/ncomms11637 (PMC4873669; doi:10.1038/ncomms11637)
Supplement: Supplementary Information — Supplementary Figures 1-82, Supplementary Tables 1-14, Supplementary Discussion and Supplementary Methods [file ncomms11637-s1.pdf]

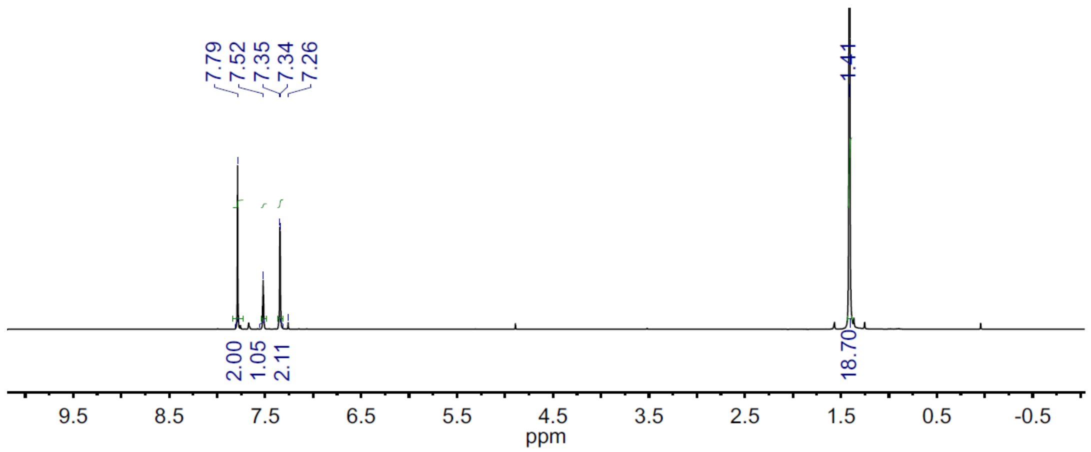

Supplementary Figure 1 | ^{1}H NMR spectrum of 7.

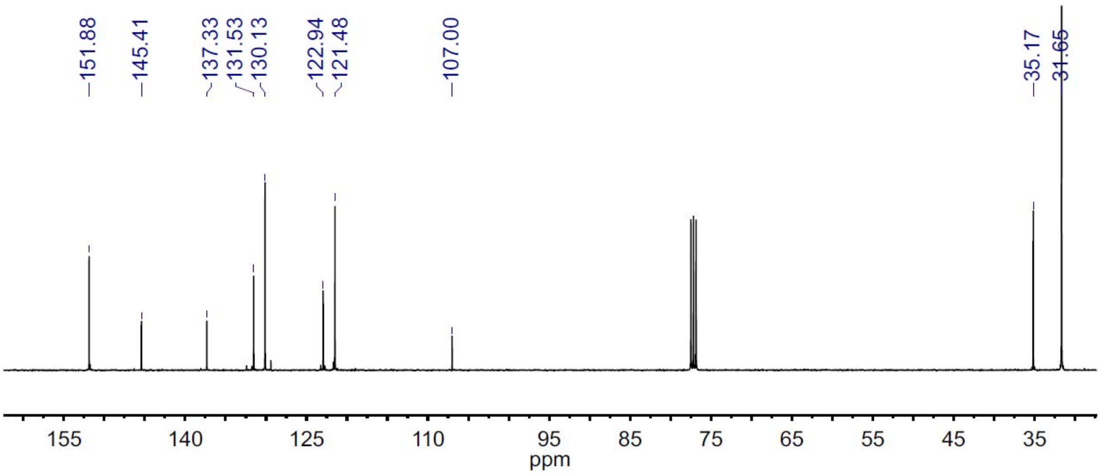

Supplementary Figure 2 | ^{13}C NMR spectrum of 7.

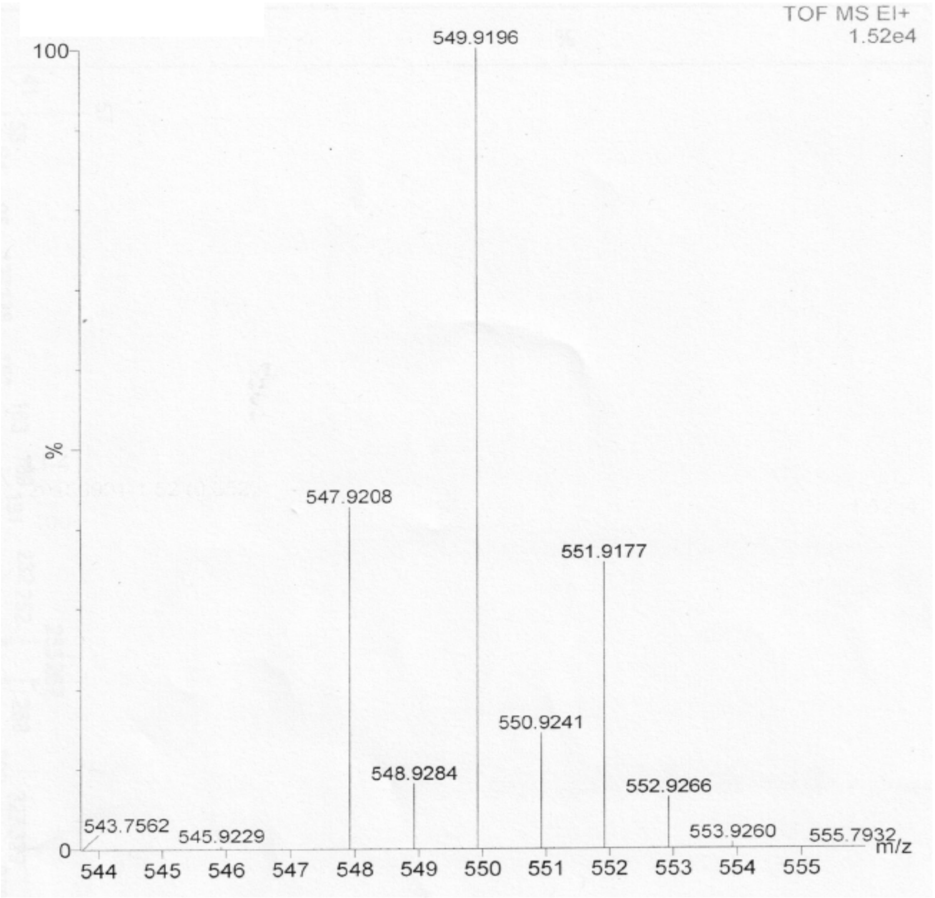

Supplementary Figure 3 | Positive mode EI TOF mass spectrum of 7.

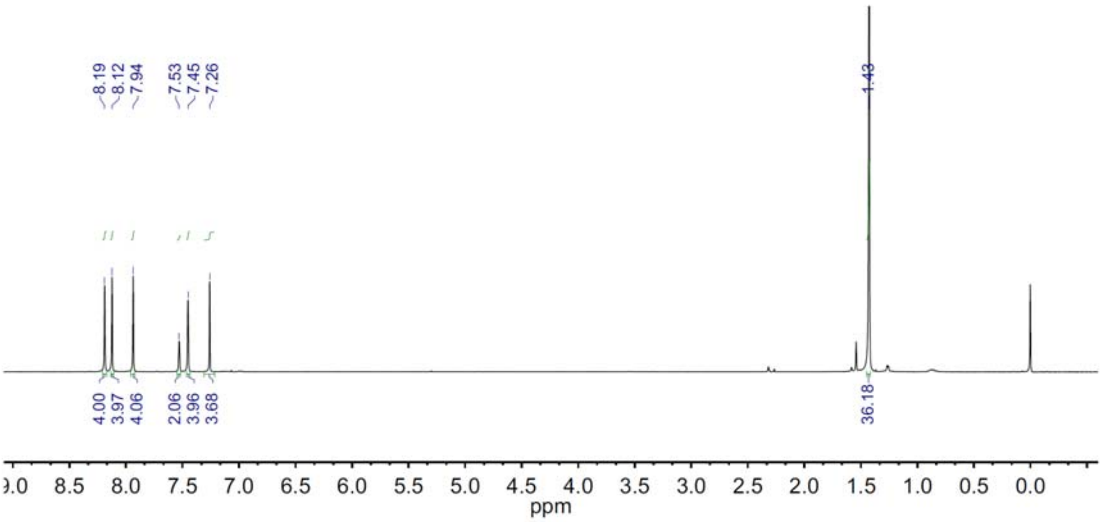

Supplementary Figure 4 | ^{1}H NMR spectrum of 8.

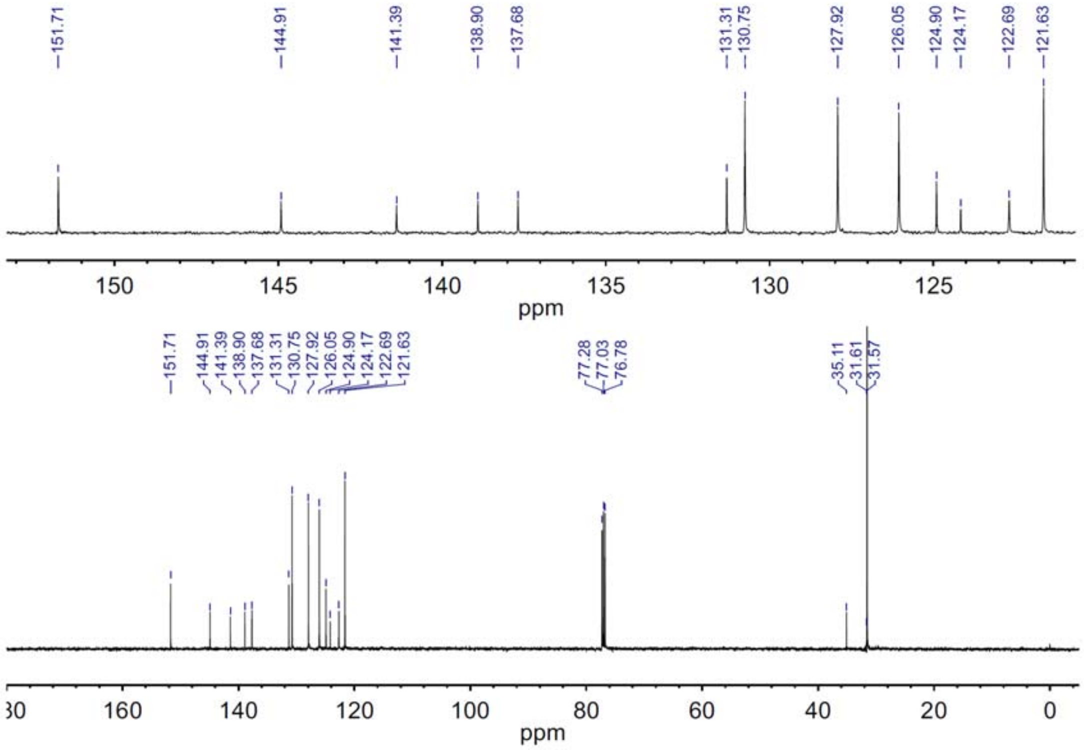

Supplementary Figure 5 | ^{13}C NMR spectrum of 8.

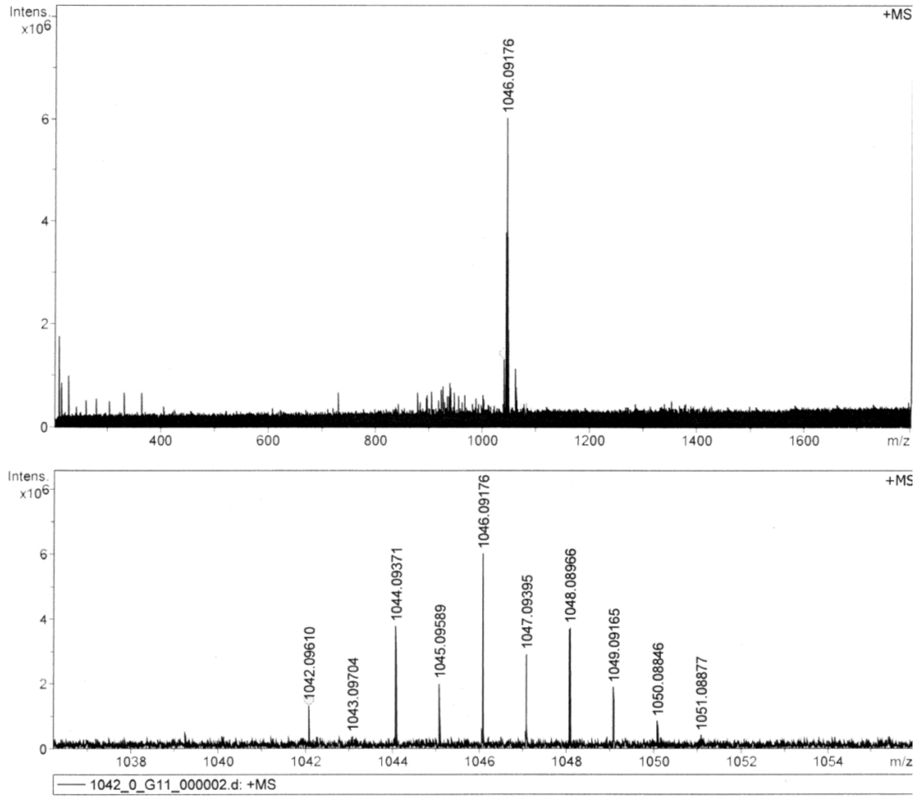

Meas. m/z	#	Ion Formula	Score	m/z	err [ppm]	Mean err [ppm]	mSigma	rdb	e⁻ Conf	N-Rule
1042.096104	1	C56H54Br4	100.00	1042.095352	0.7	0.8	68.4	28.0	odd	ok

Supplementary Figure 6 | Positive mode MALDI TOF mass spectrum of 8.

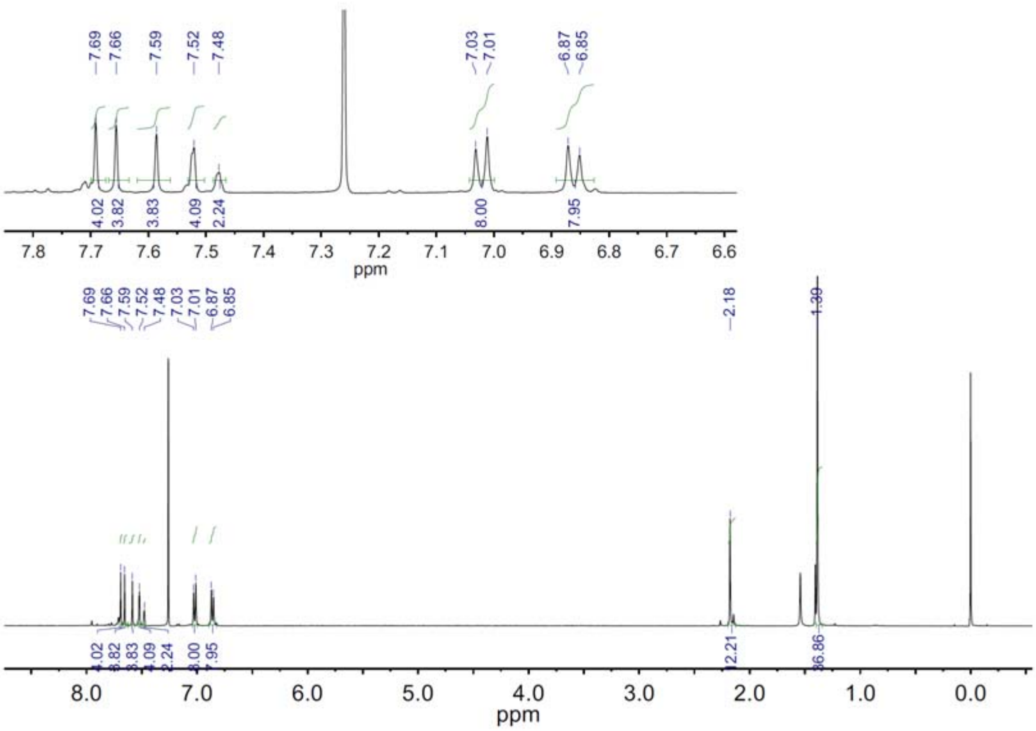

Supplementary Figure 7 | ^{1}H NMR spectrum of 10a

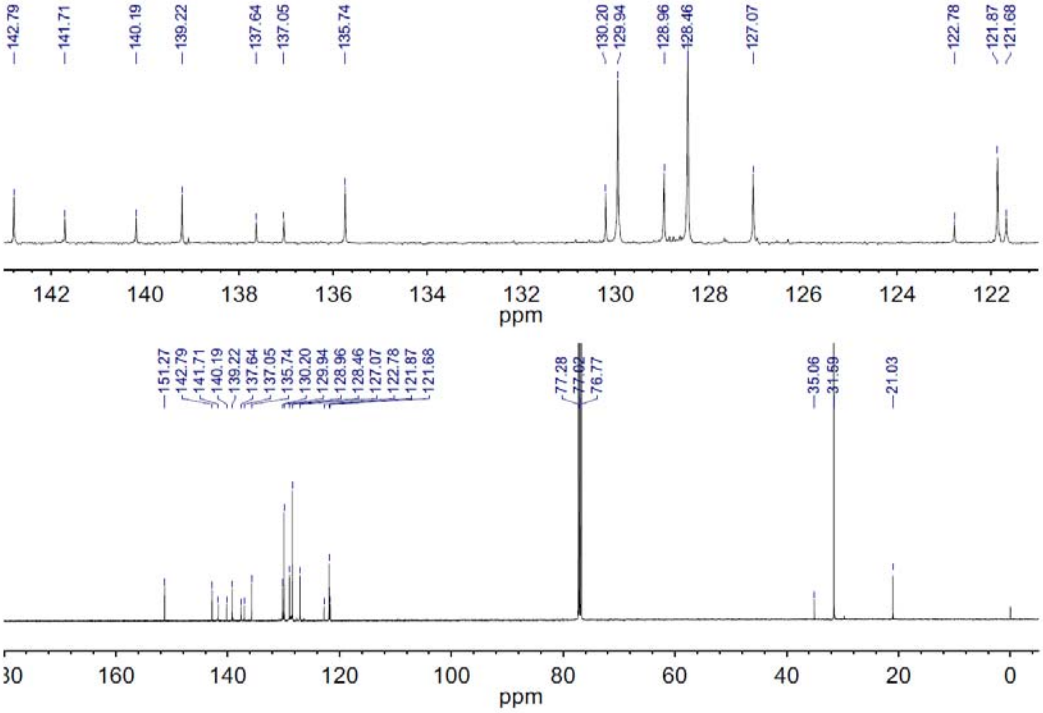

Supplementary Figure 8 | ^{13}C NMR spectrum of 10a

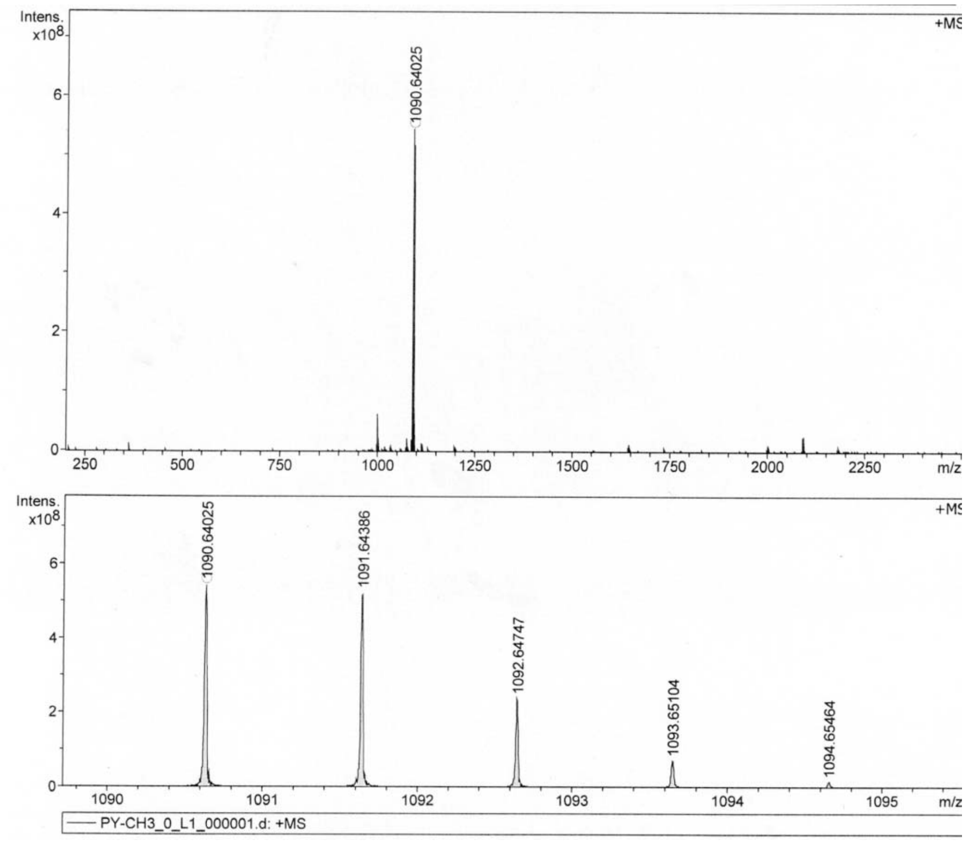

Supplementary Figure 9 | Positive mode MALDI TOF mass spectrum of 10a.

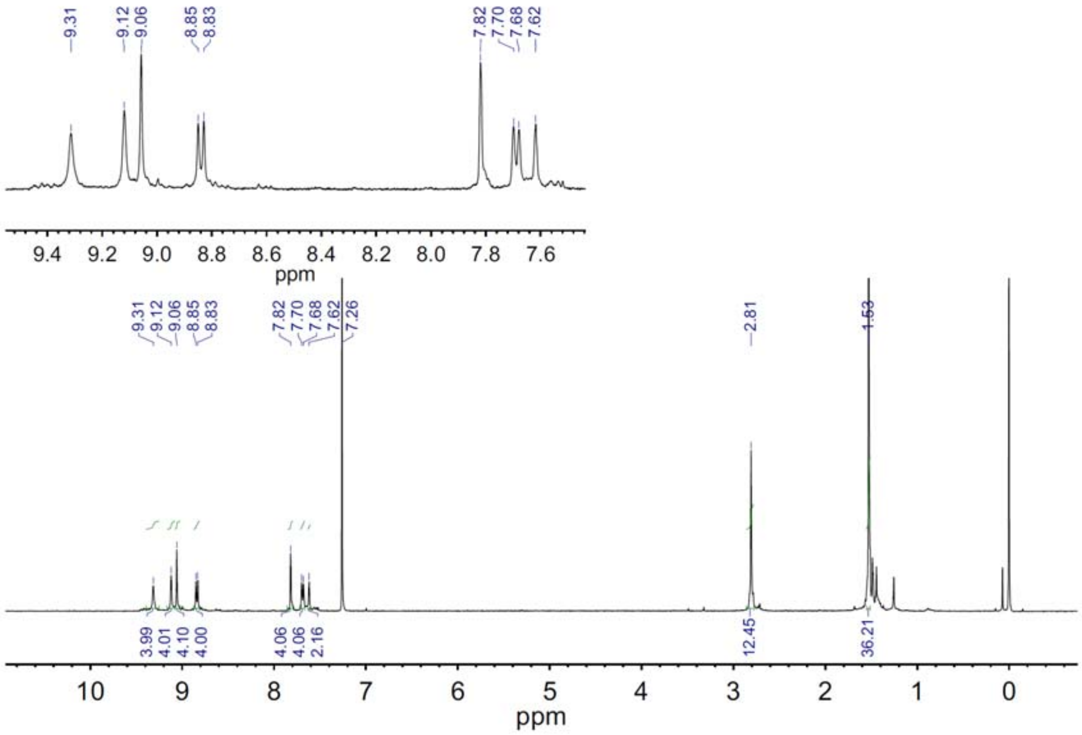

Supplementary Figure 10 | ^{1}H NMR spectrum of 1a.

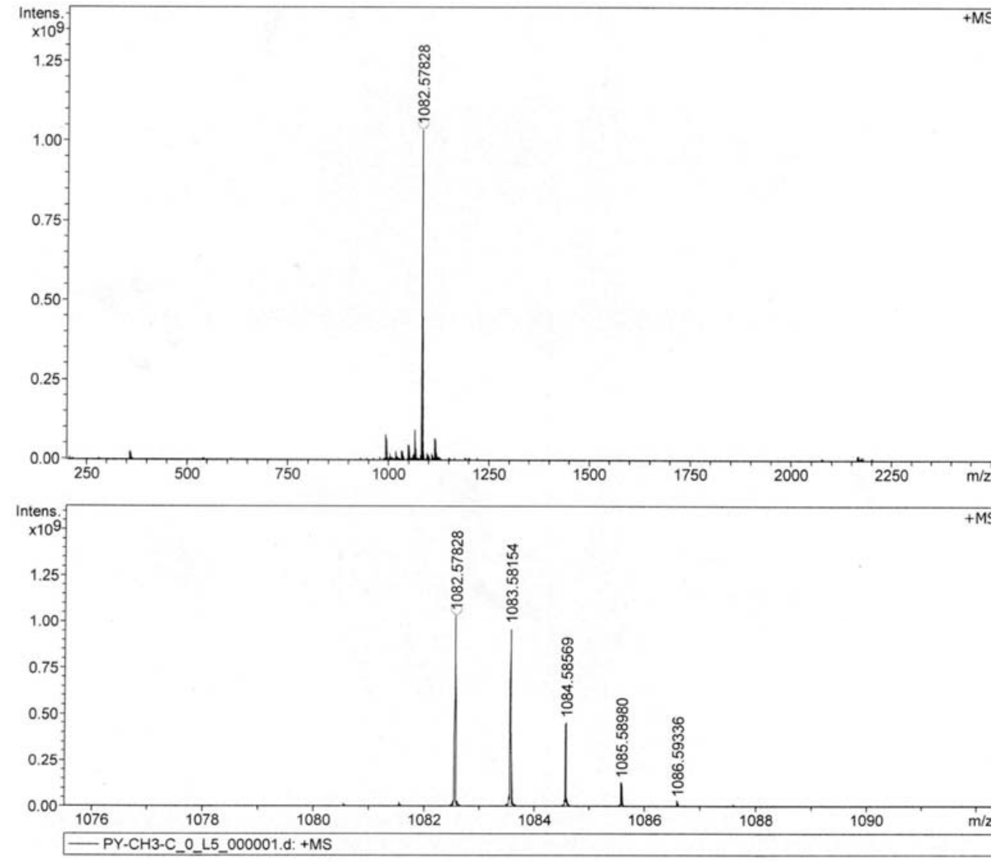

Meas. m/z	#	Ion Formula	Score	m/z	err [ppm]	Mean err [ppm]	mSigma	rdb	e⁻ Conf	N-Rule
1082.578279	1	C84H74	100.00	1082.578504	0.2	0.1	12.4	48.0	odd	ok

Supplementary Figure 11 | Positive mode MALDI TOF mass spectrum of 1a.

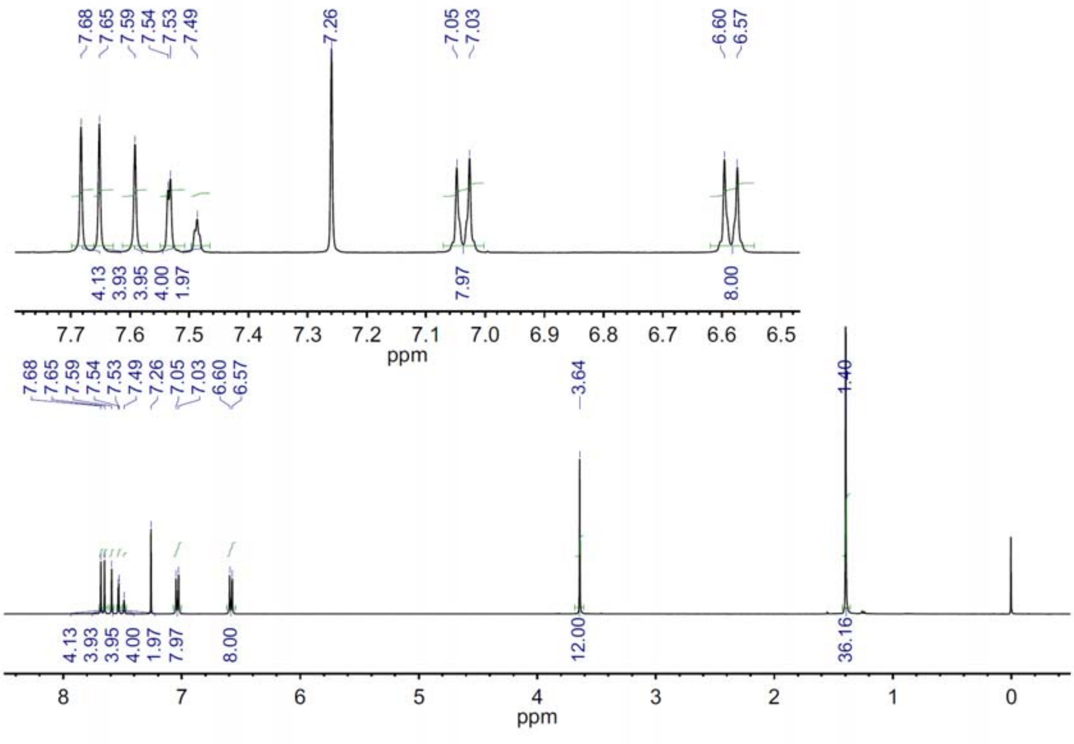

Supplementary Figure 12 | ^{1}H NMR spectrum of 10b.

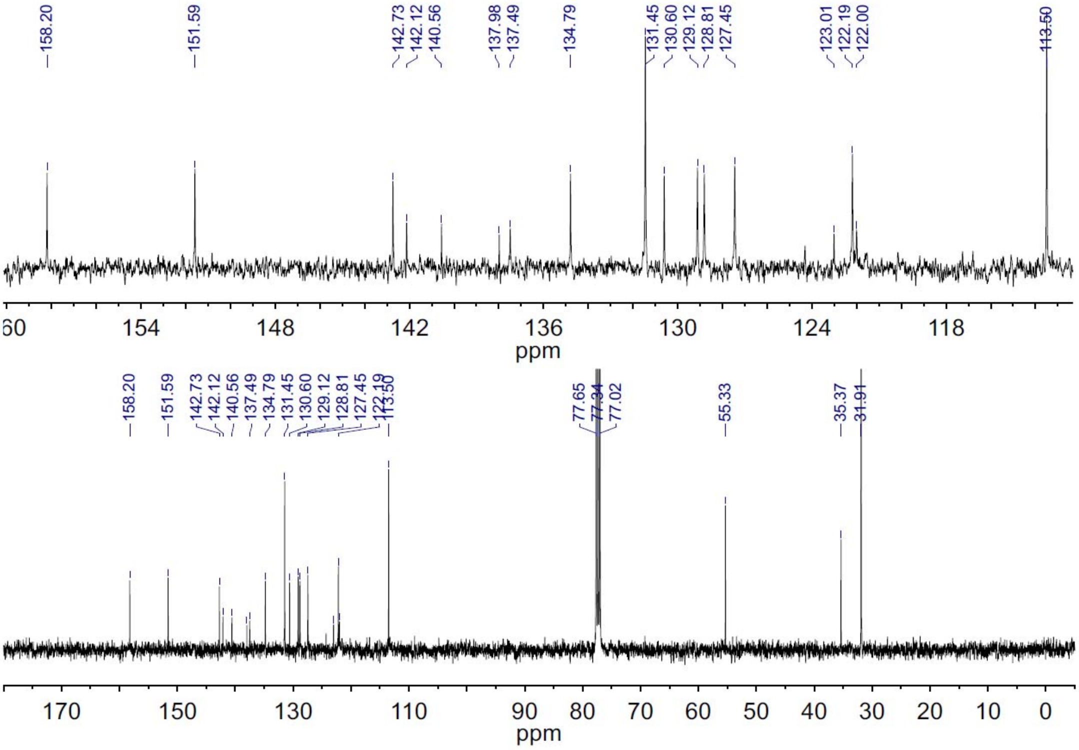

Supplementary Figure13 | ^{13}C NMR spectrum of 10b.

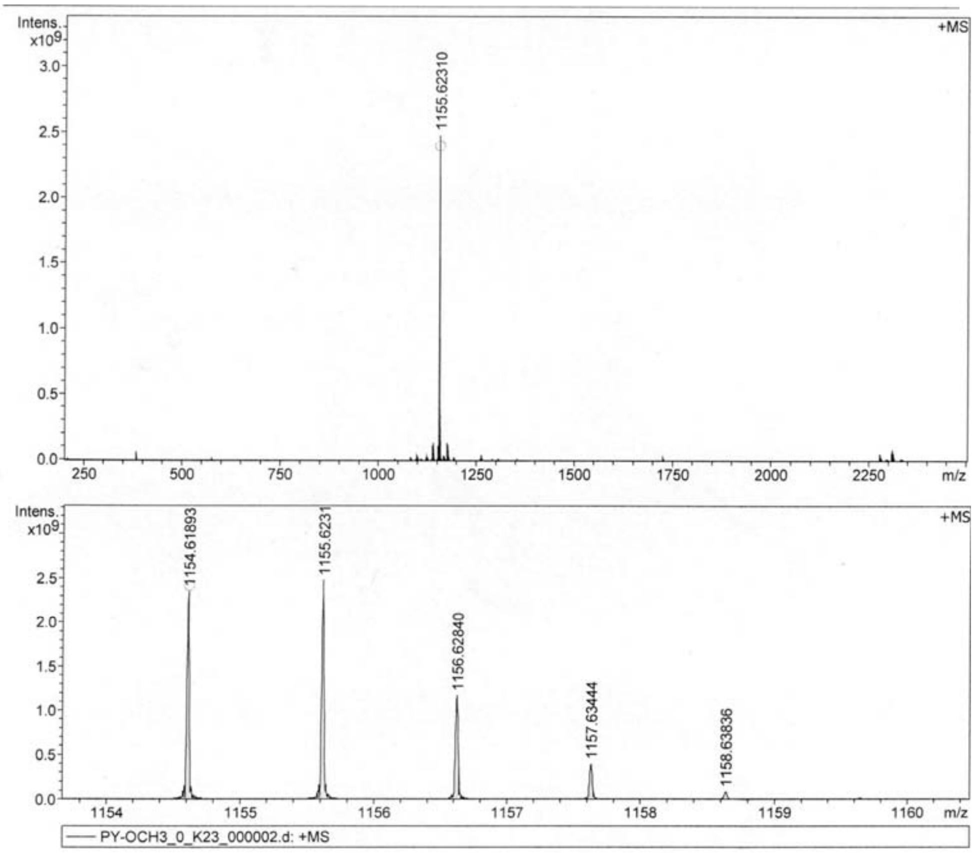

Meas. m/z	#	Ion Formula	Score	m/z	err [ppm]	Mean err [ppm]	mSigma	rdb	e⁻ Conf	N-Rule
1154.618928	1	C84H82O4	100.00	1154.620763	-1.6	0.5	44.4	44.0	odd	ok

Supplementary Figure 14 | Positive mode MALDI TOF mass spectrum of 10b.

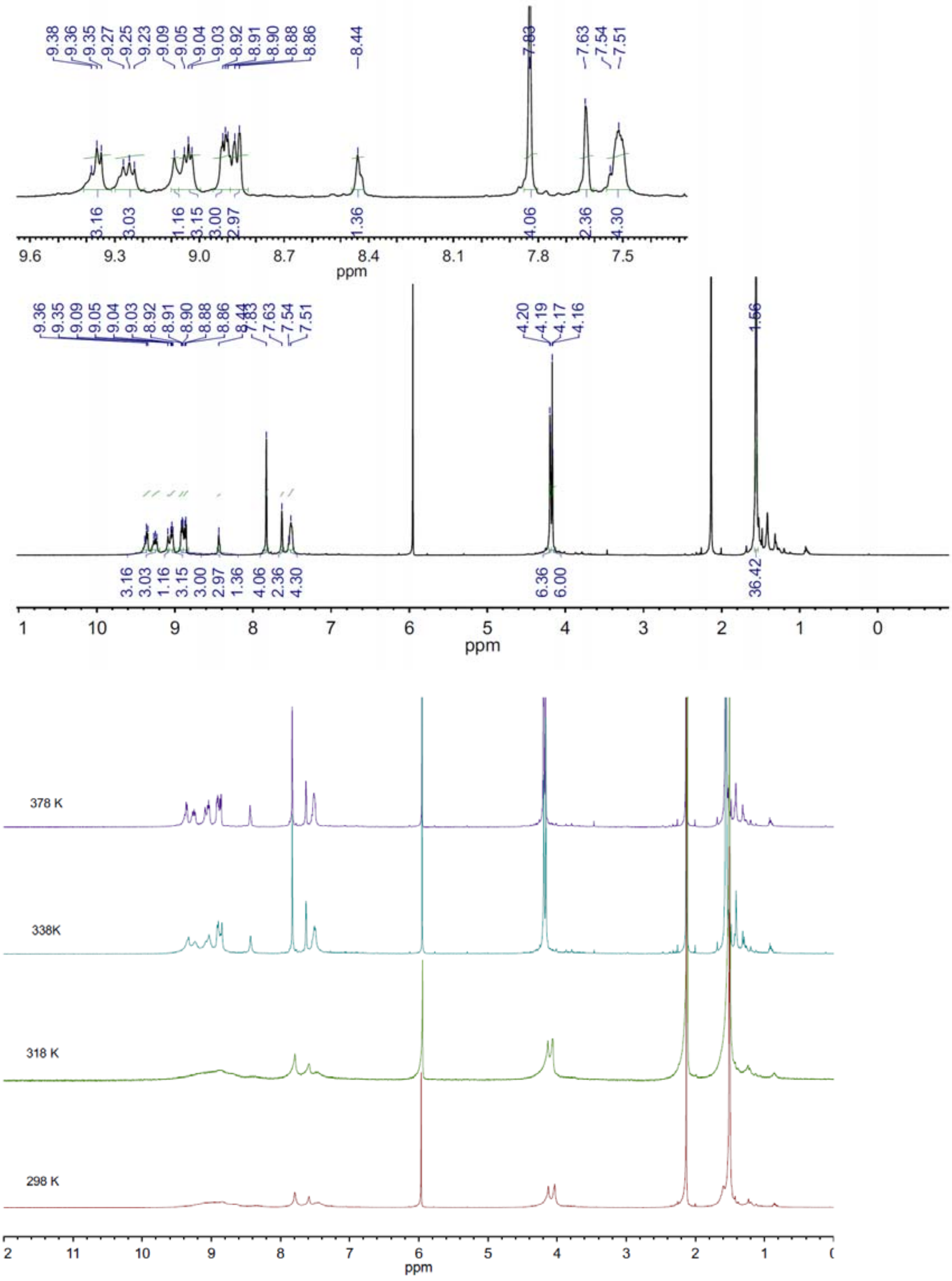

Supplementary Figure 15 | ^{1}H NMR and variable temperature ^{1}H NMR spectra of 1b.

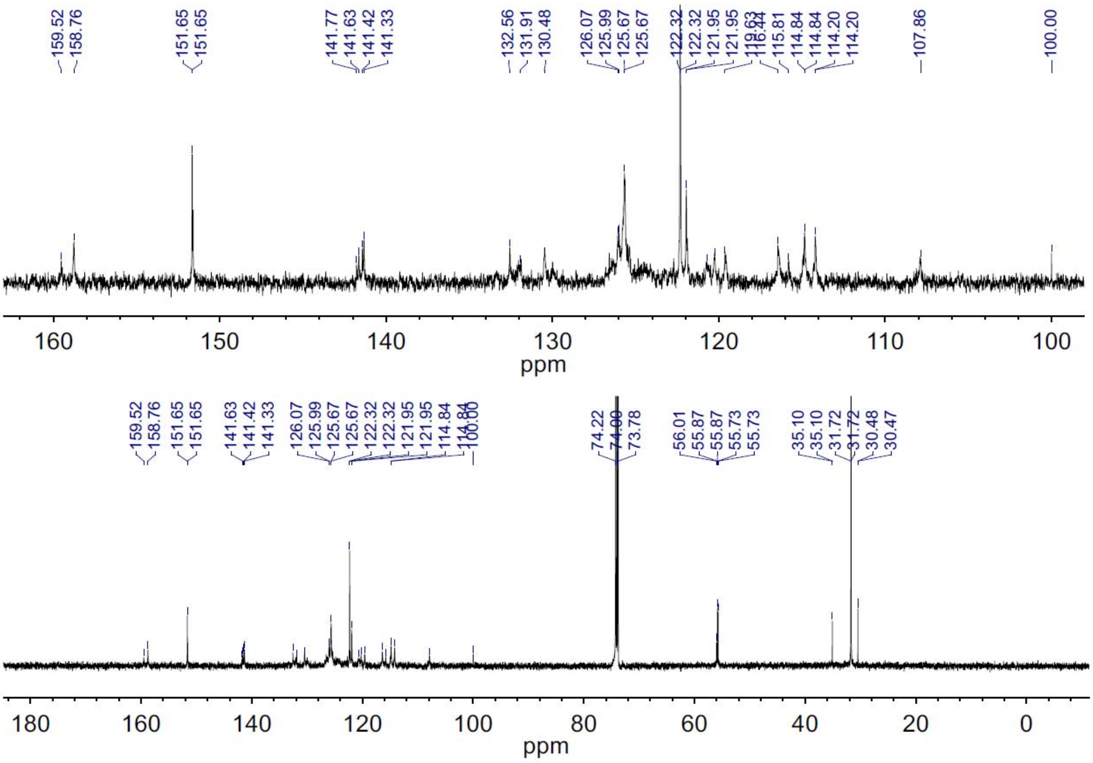

Supplementary Figure 16 | ^{13}C NMR spectrum of 1b.

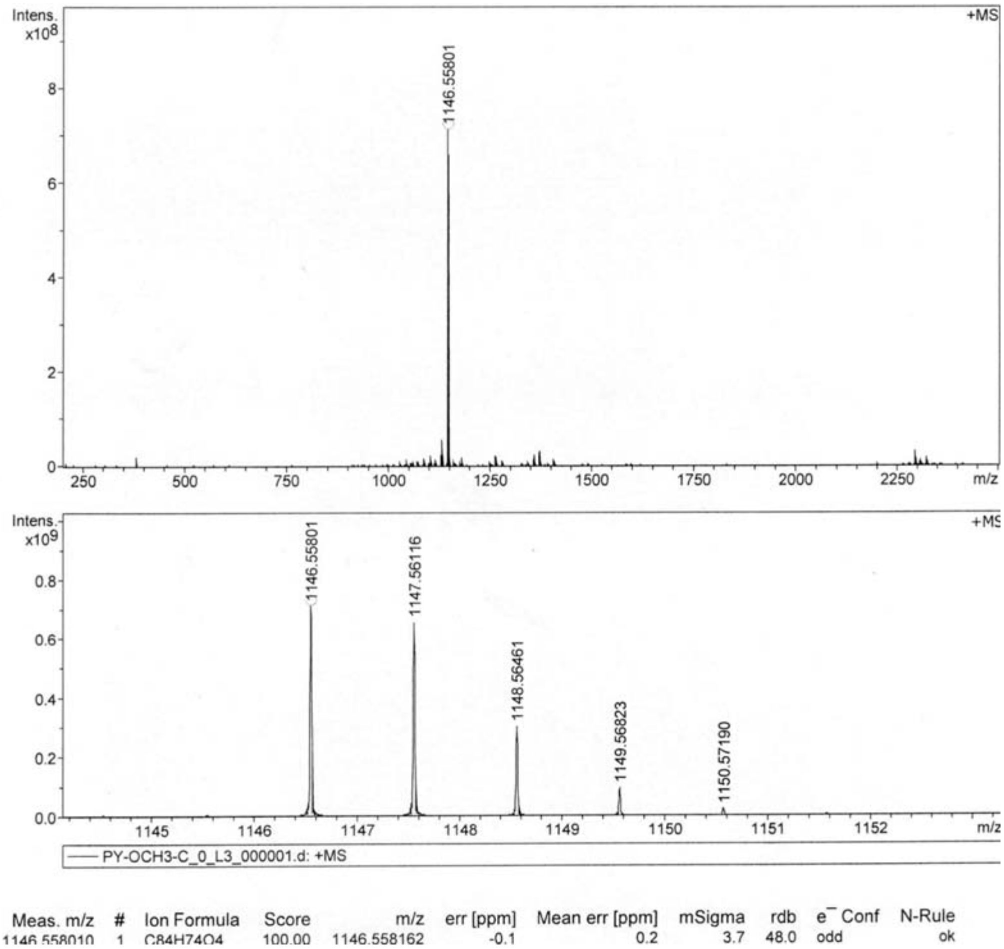

Supplementary Figure 17 | Positive mode MALDI TOF mass spectrum of 1b.

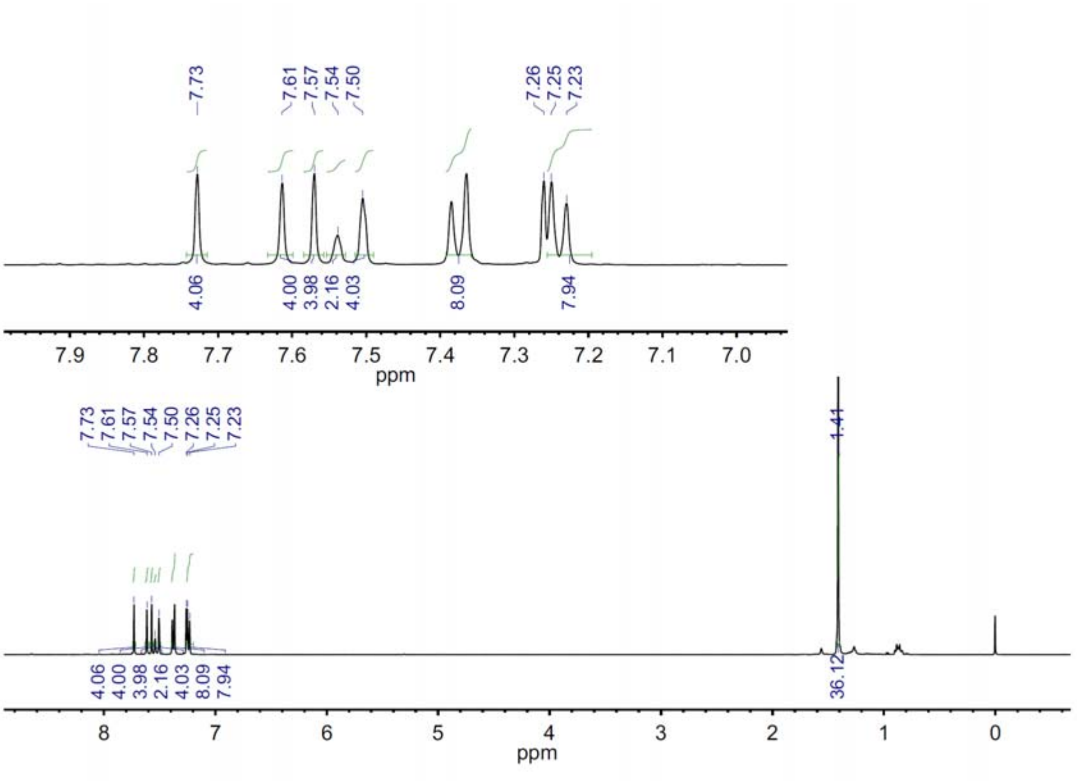

Supplementary Figure 18 | ^{1}H NMR spectra of 10c.

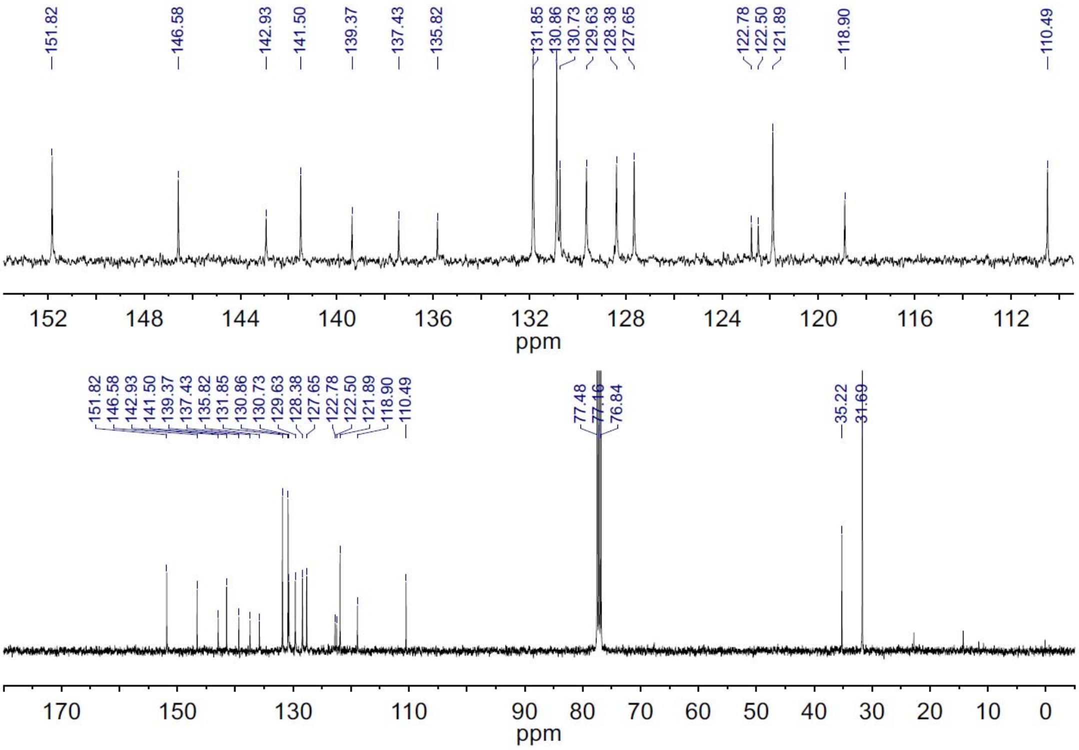

Supplementary Figure 19 | ^{13}C NMR spectra of 10c.

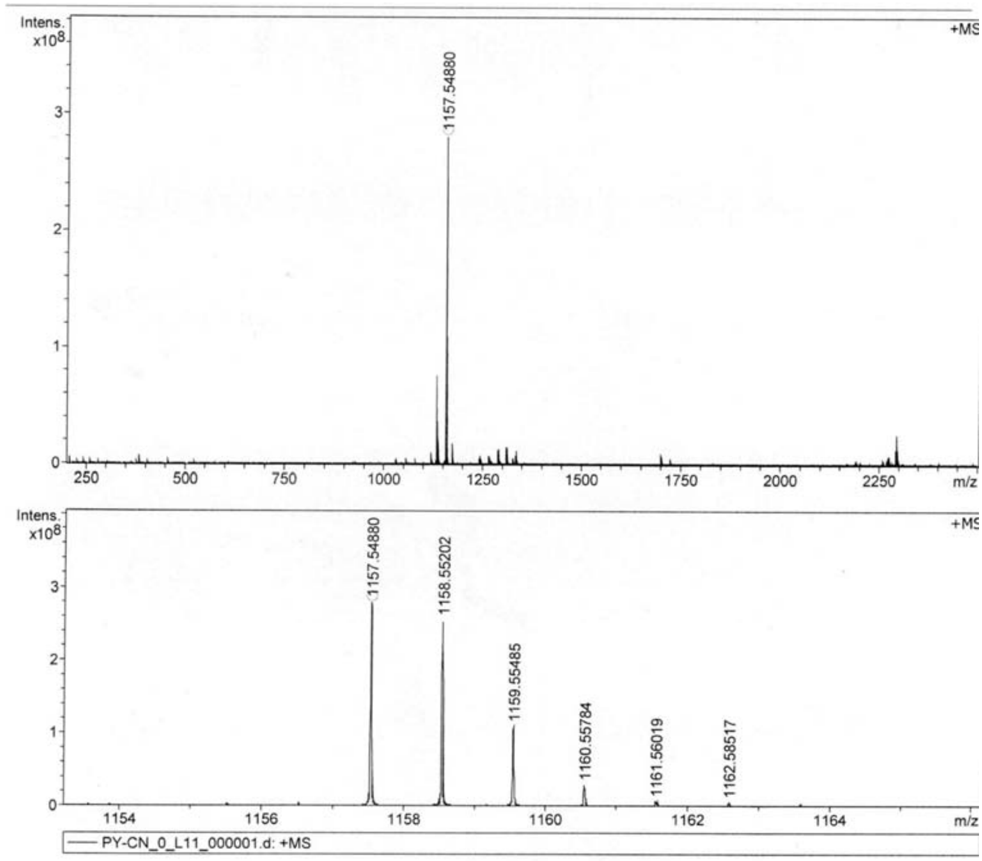

Meas. m/z	#	Ion Formula	Score	m/z	err [ppm]	Mean err [ppm]	mSigma	rdb	e⁻ Conf	N-Rule
1157.548805	1	C84H70N4Na	100.00	1157.549269	0.4	0.5	21.5	51.5	even	ok

Supplementary Figure 20 | Positive mode MALDI TOF mass spectrum of 10c.

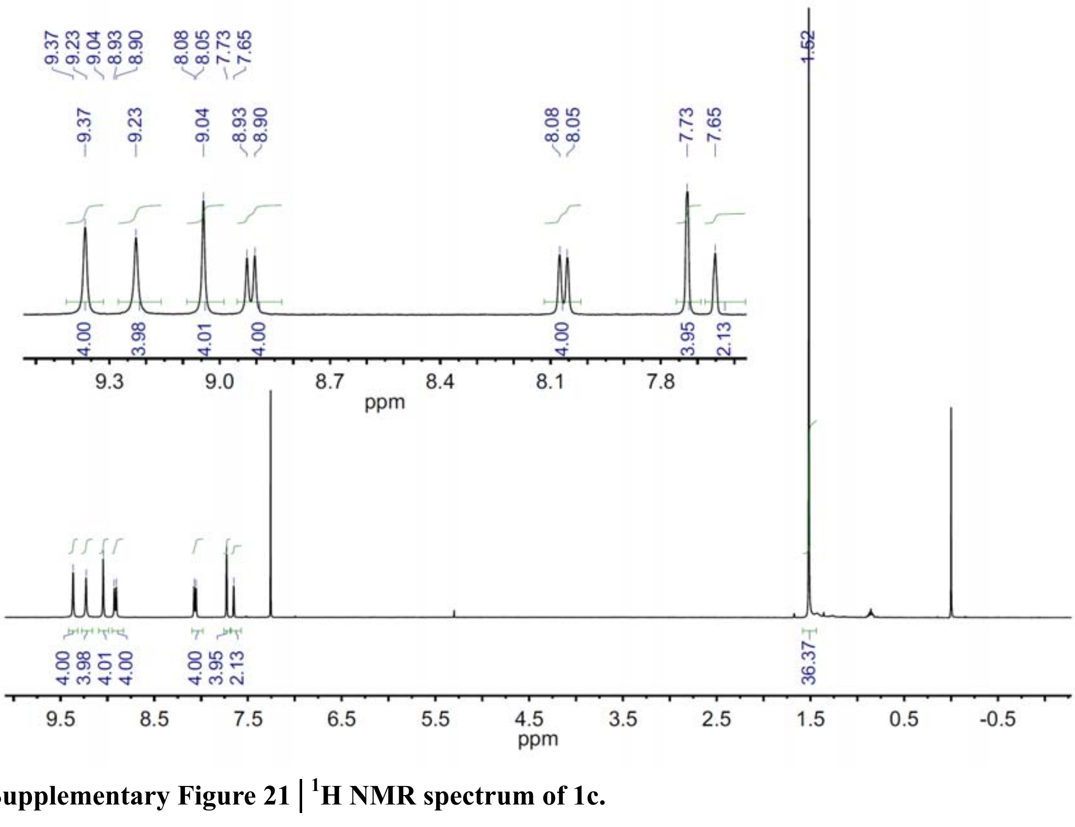

Supplementary Figure 21 | ^{1}H NMR spectrum of 1c.

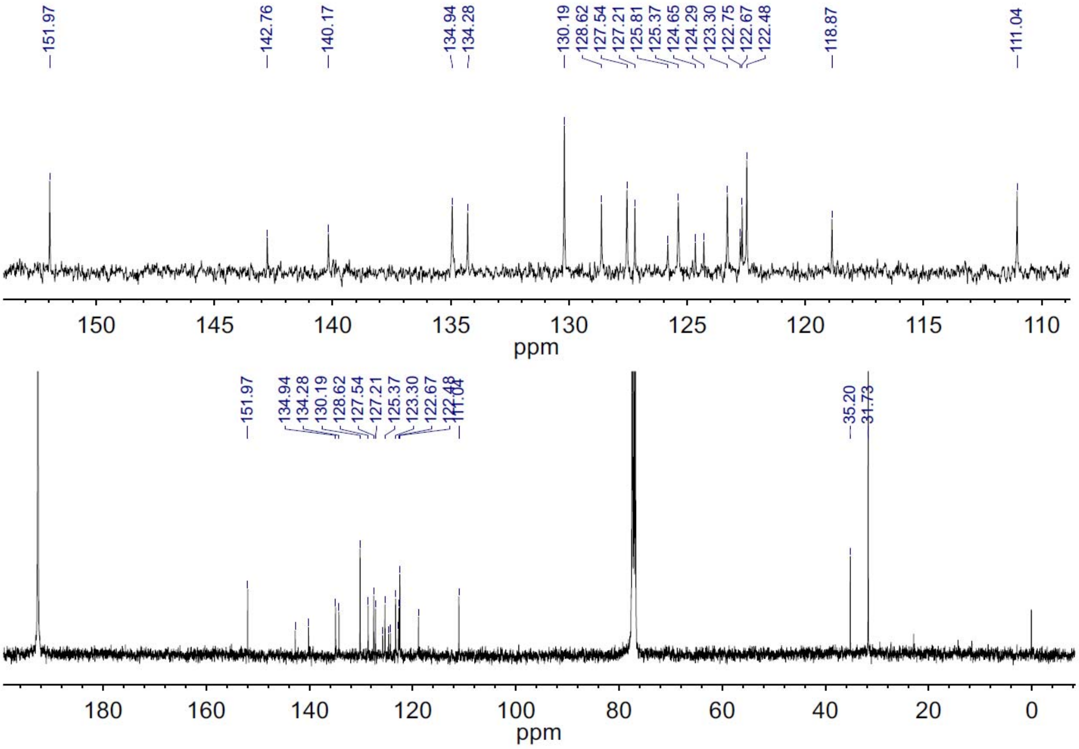

Supplementary Figure 22 | ^{13}C NMR spectra of 1c.

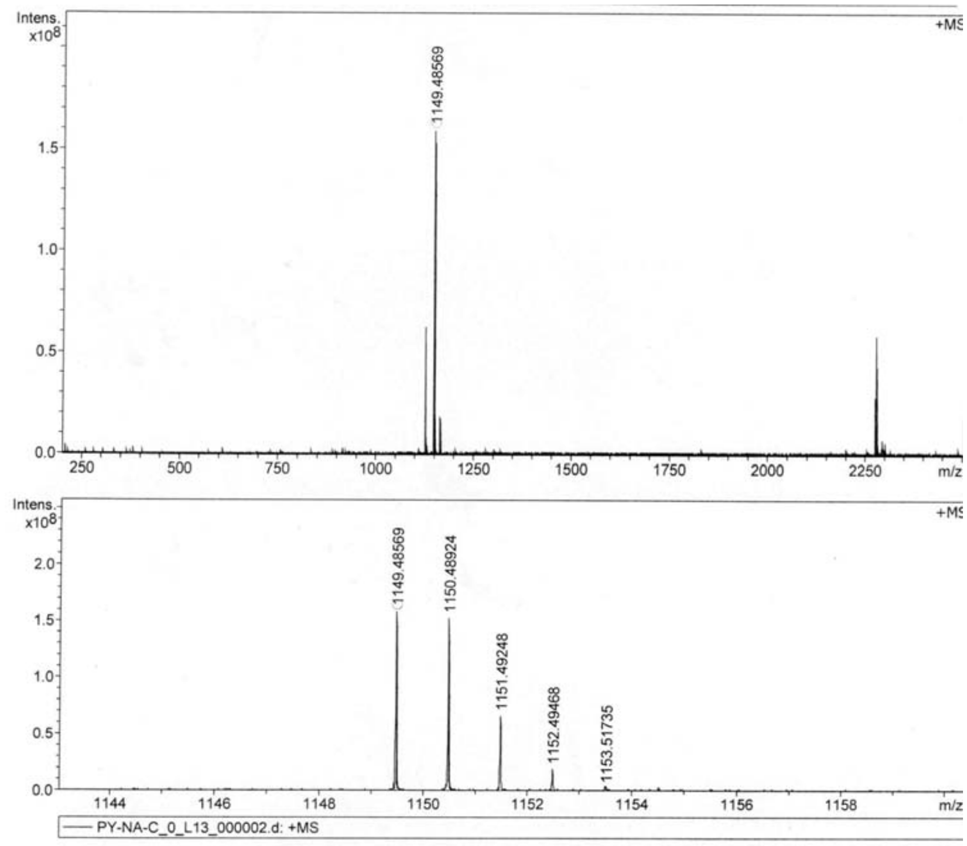

Meas. m/z	#	Ion Formula	Score	m/z	err [ppm]	Mean err [ppm]	mSigma	rdb	e⁻ Conf	N-Rule
1149.485693	1	C84H62N4Na	100.00	1149.486669	-0.8	0.8	17.9	55.5	even	ok

Supplementary Figure 23 | Positive mode MALDI TOF mass spectrum of 1c.

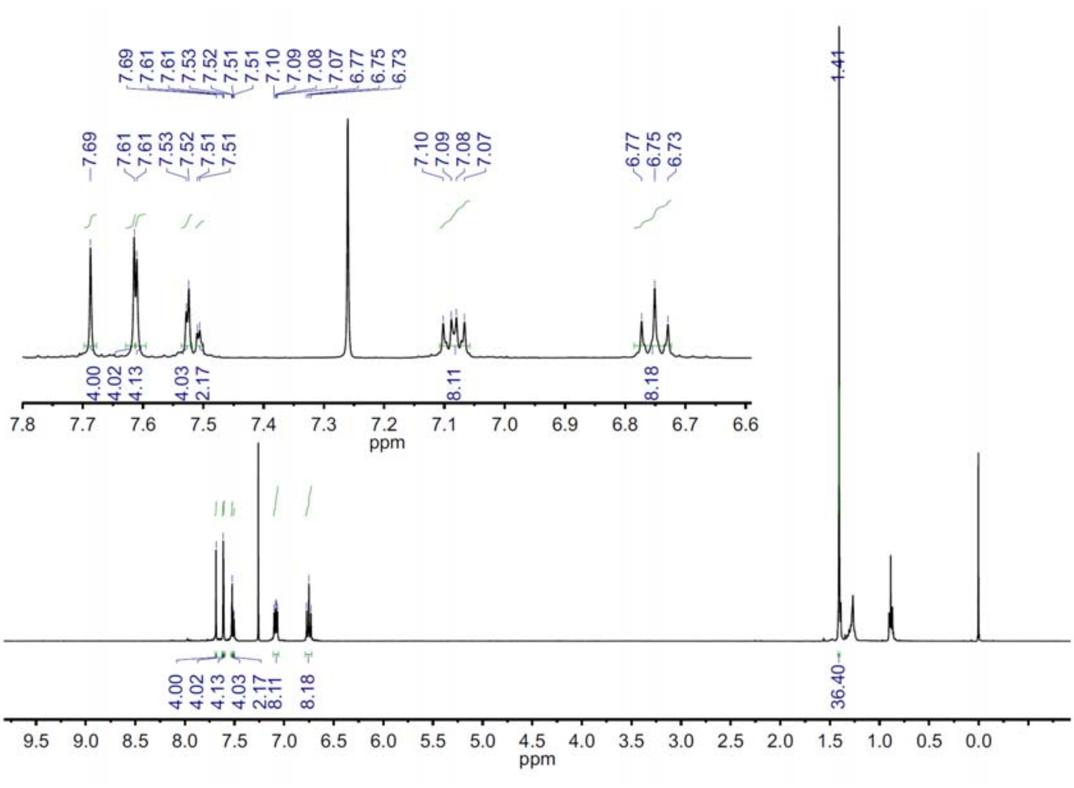

Supplementary Figure 24 | ^{1}H NMR spectrum of 10d.

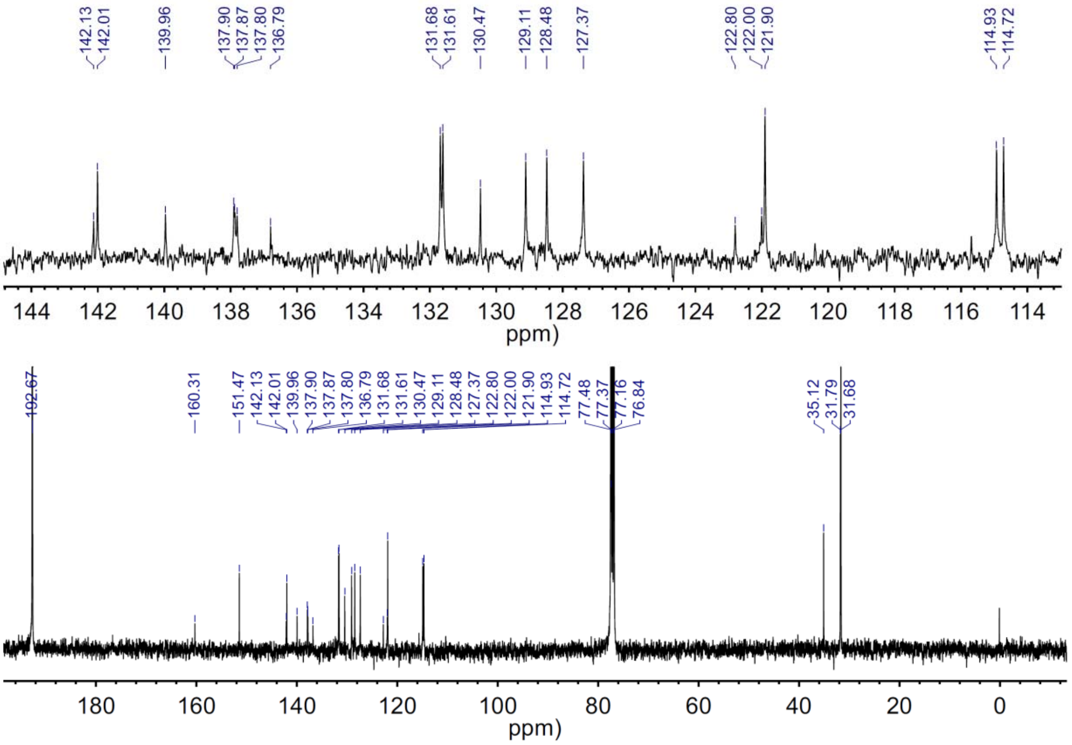

Supplementary Figure 25 | ^{13}C NMR spectrum of 10d.

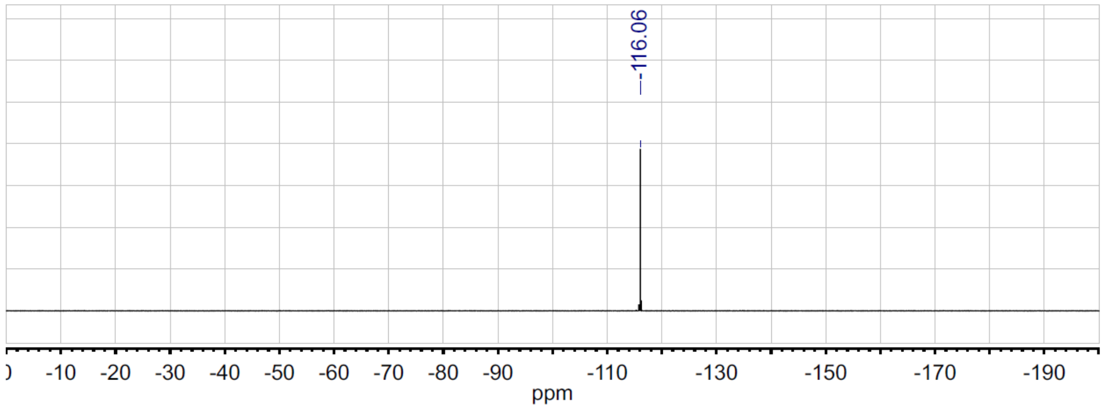

Supplementary Figure 26 | ^{19}F NMR spectrum of 10d.

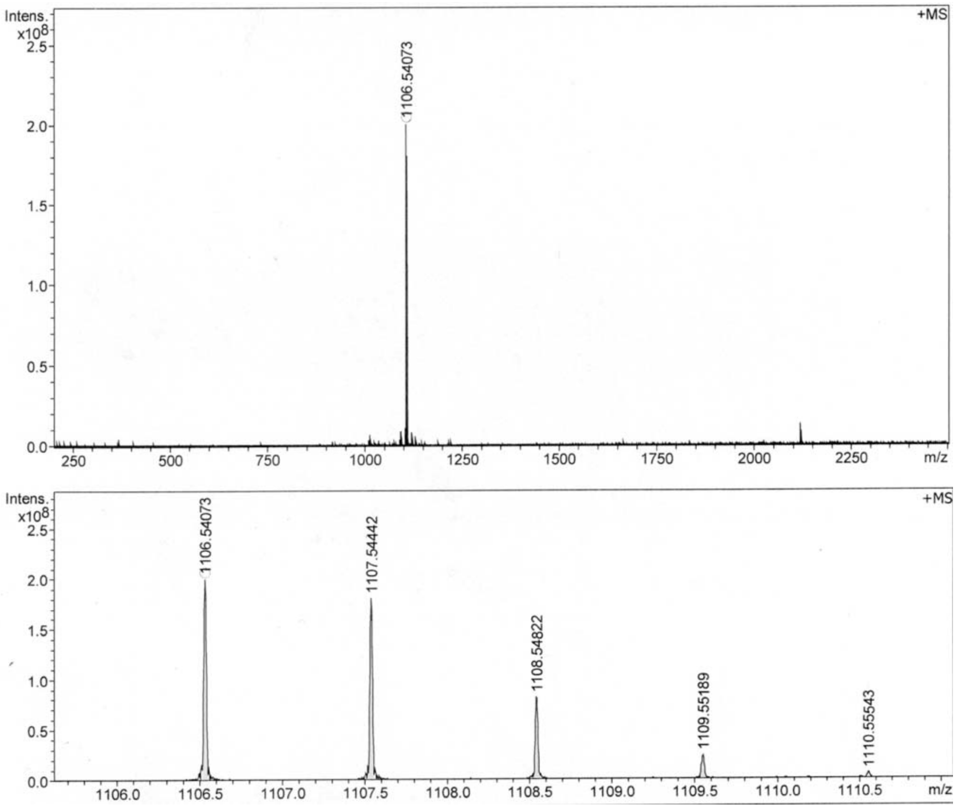

Supplementary Figure 27 | Positive mode MALDI TOF mass spectrum of 10d.

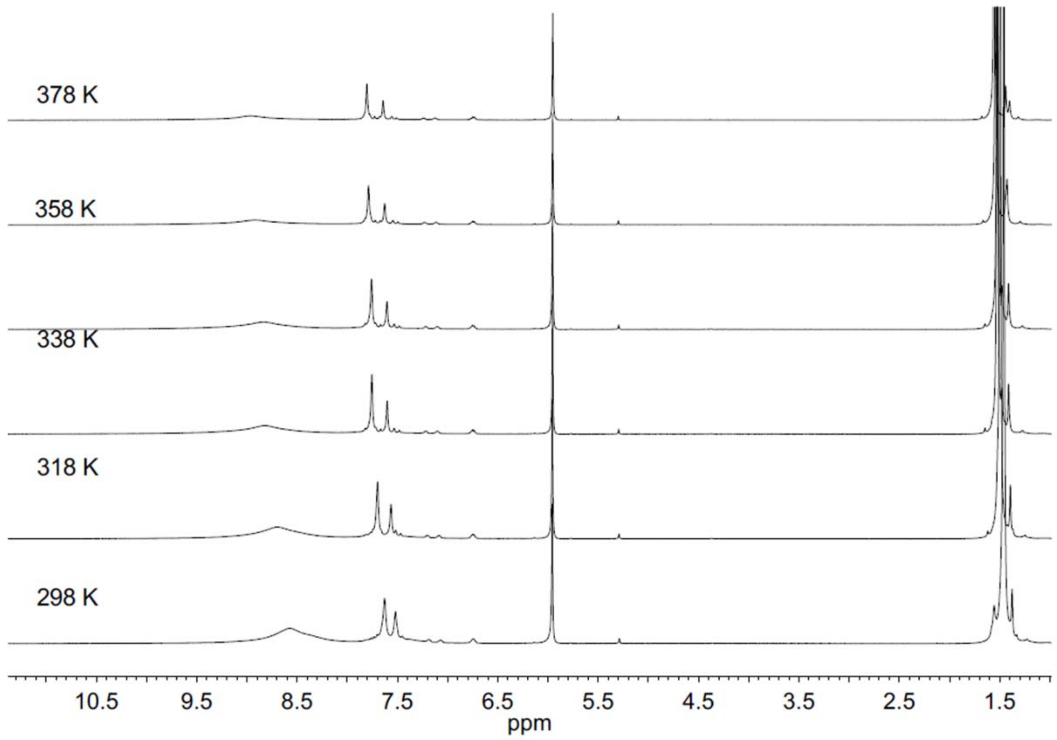

Supplementary Figure 28 | Variable temperature ^{1}H NMR spectra of 1d.

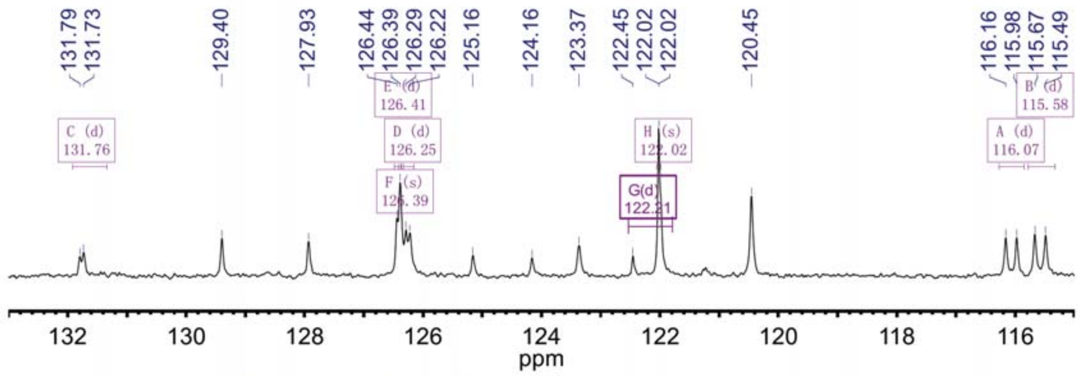

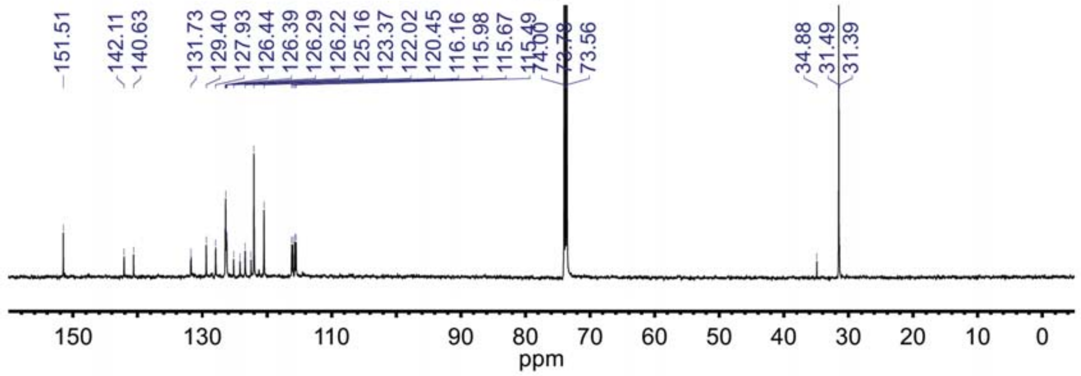

Supplementary Figure 29 | ^{13}C NMR spectrum of 1d.

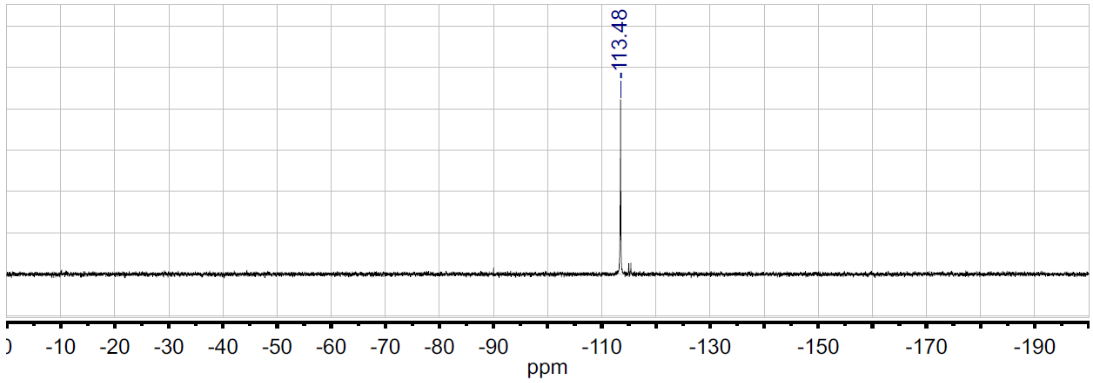

Supplementary Figure 30 | ^{19}F NMR spectrum of 1d.

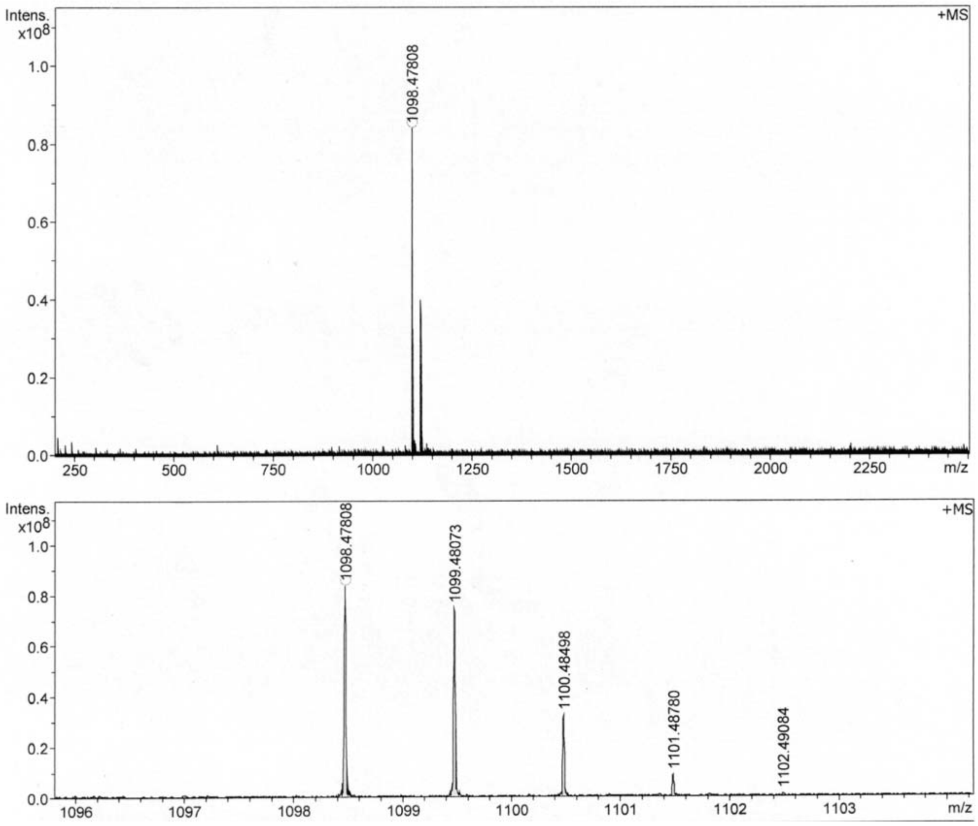

Supplementary Figure 31 | Positive mode MALDI TOF mass spectrum of 1d.

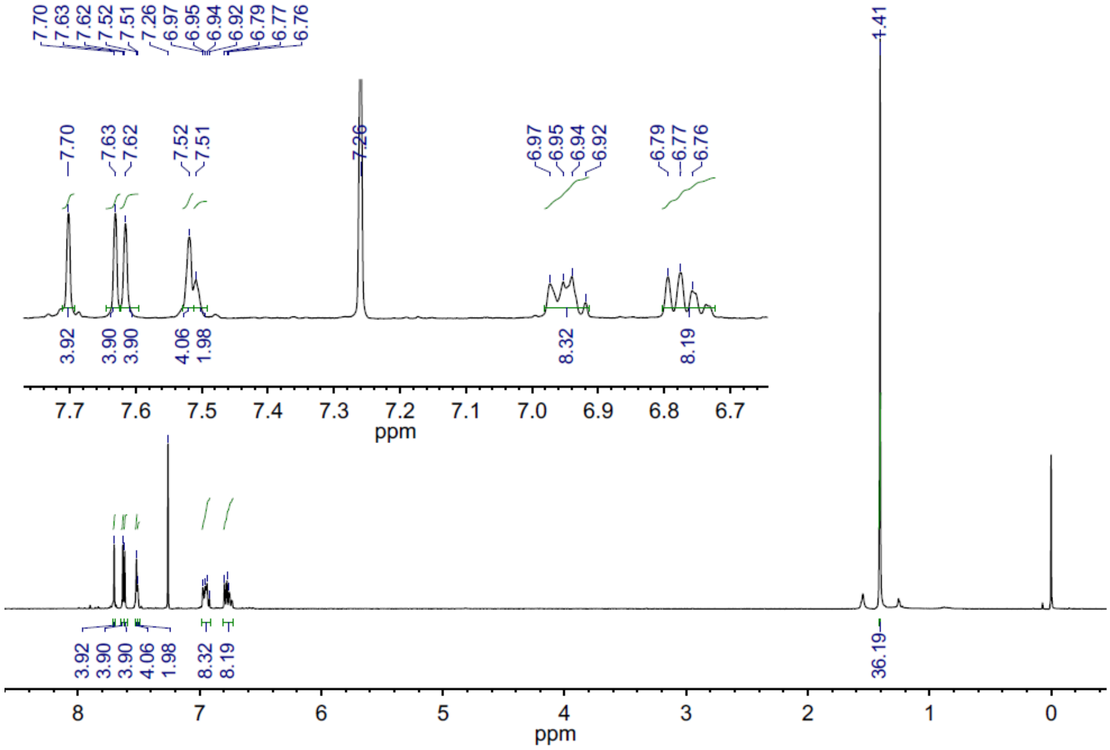

Supplementary Figure 32 | ^{1}H NMR spectrum of 10e.

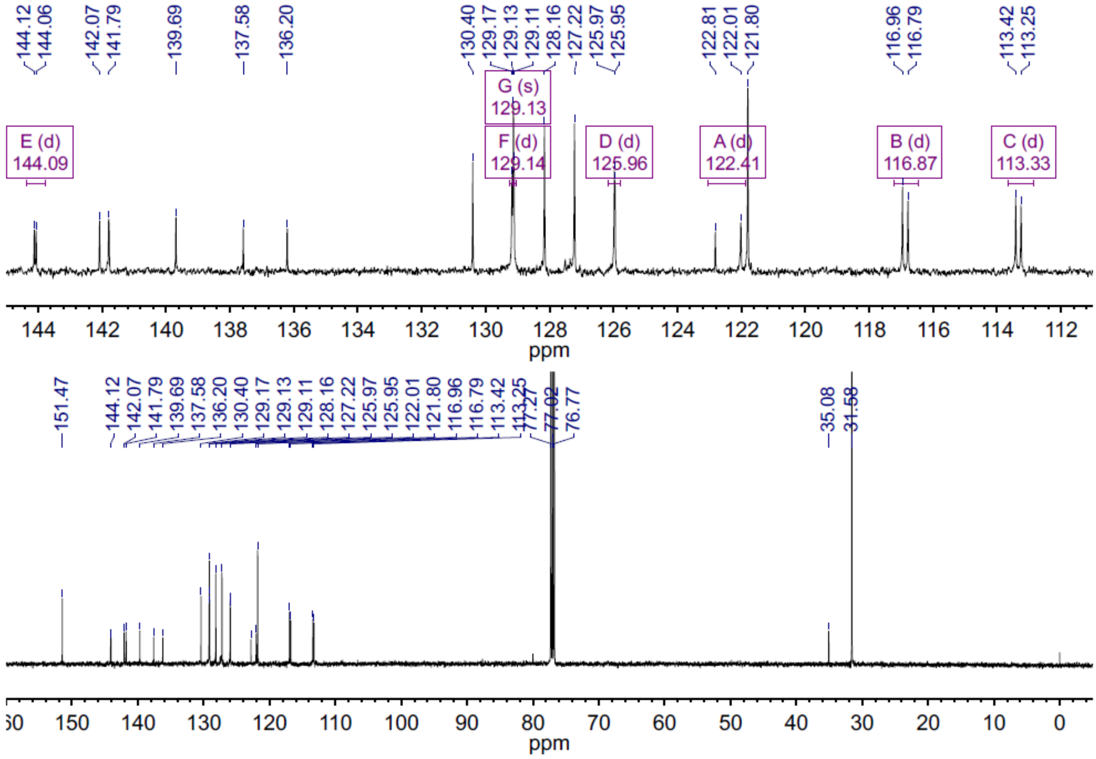

Supplementary Figure 33 | ^{13}C NMR spectrum of 10e.

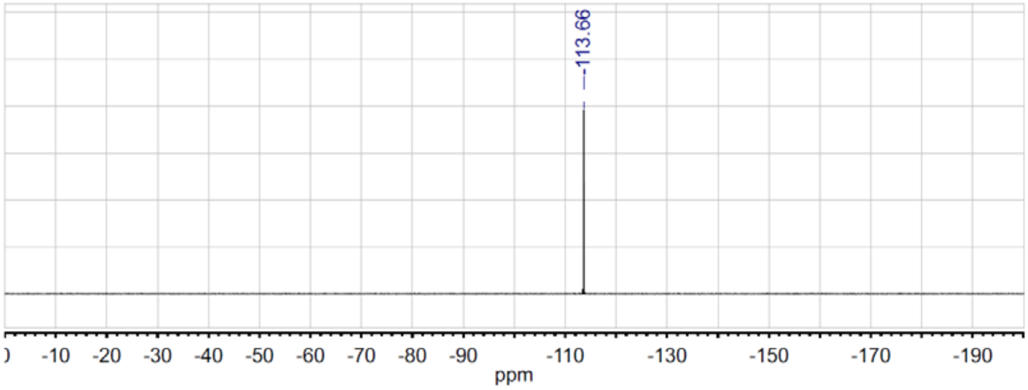

Supplementary Figure 34 | ^{19}F NMR spectrum of 10e.

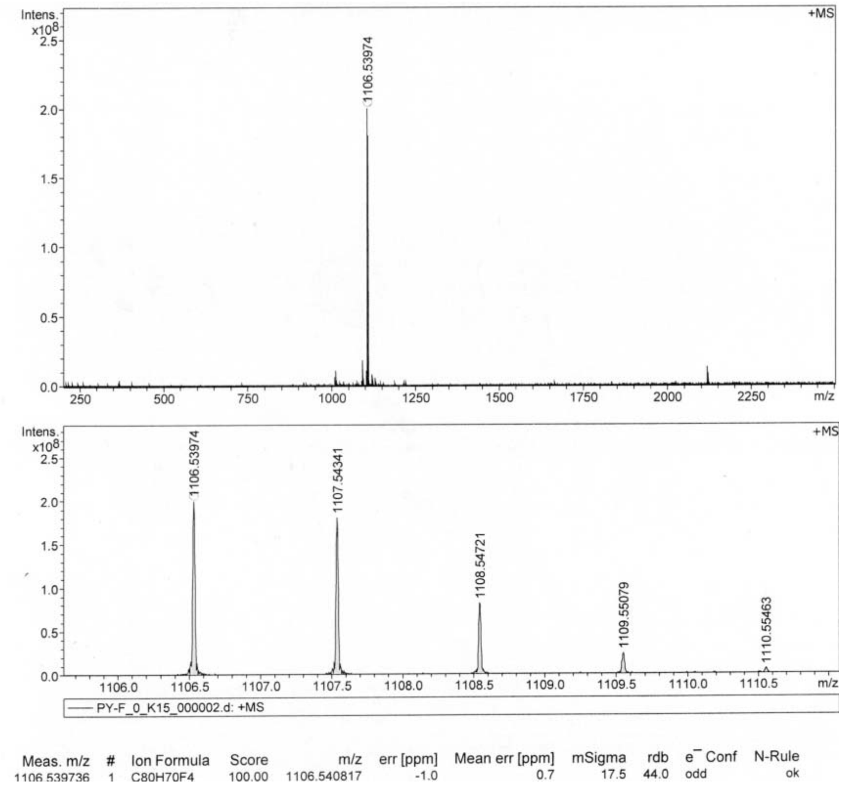

Supplementary Figure 35 | Positive mode MALDI TOF mass spectrum of 10e.

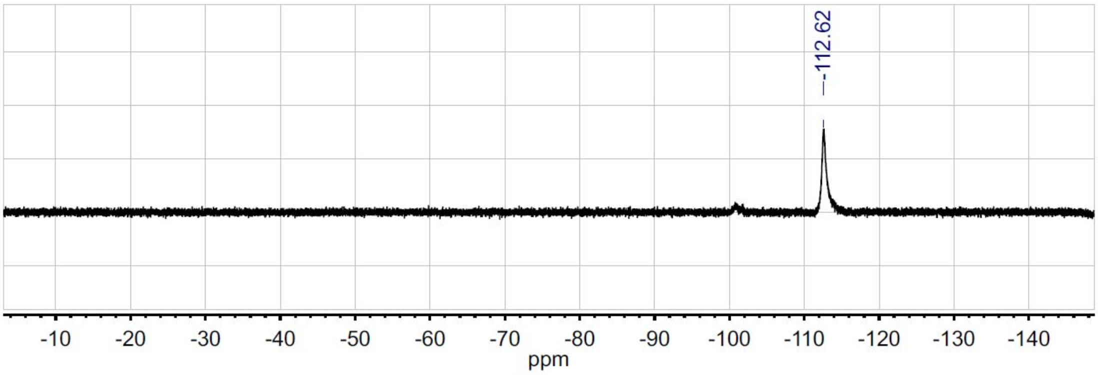

Supplementary Figure 36 | ^{19}F NMR spectra of 1e.

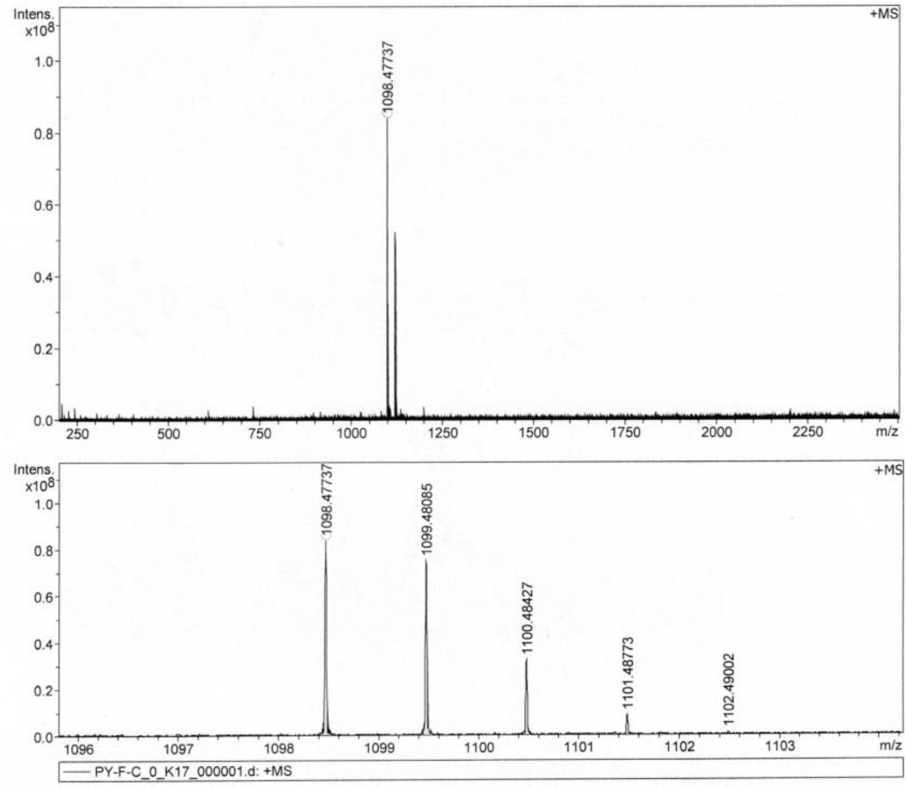

Meas. m/z	#	Ion Formula	Score	m/z	err [ppm]	Mean err [ppm]	mSigma	rdb	e⁻ Conf	N-Rule
1098.477374	1	C80H62F4	100.00	1098.478216	0.8	0.7	10.5	48.0	odd	ok

Supplementary Figure 37 | Positive mode MALDI TOF mass spectrum of 1e.

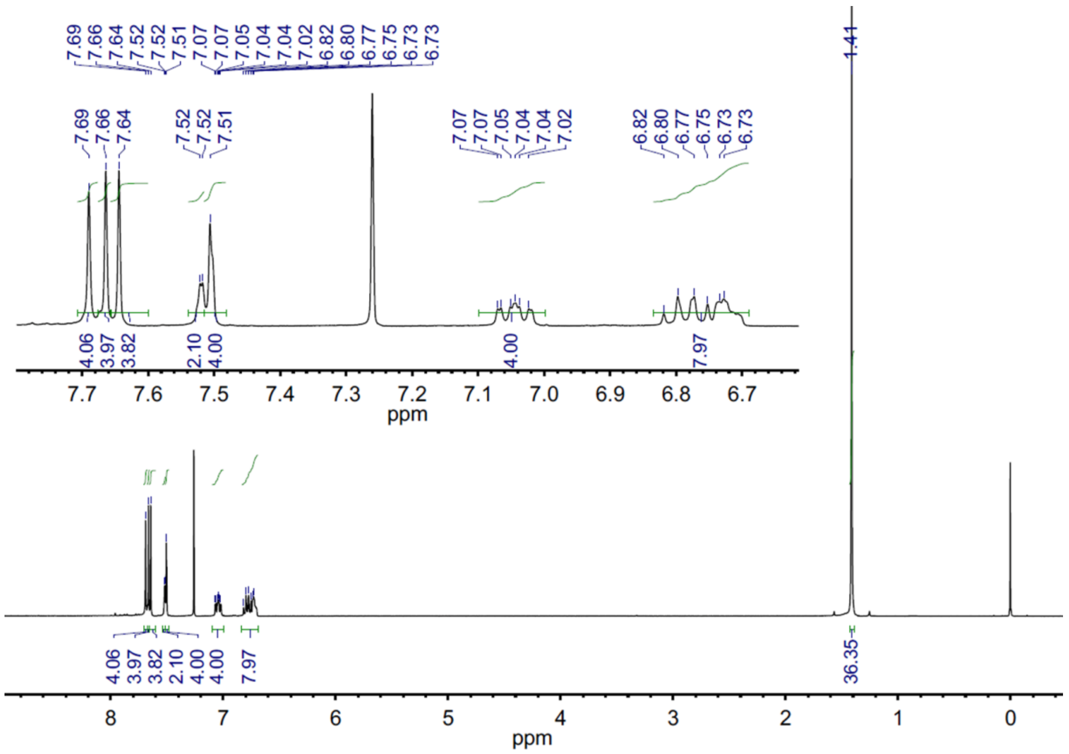

Supplementary Figure 38 | ^{1}H NMR spectrum of 10f.

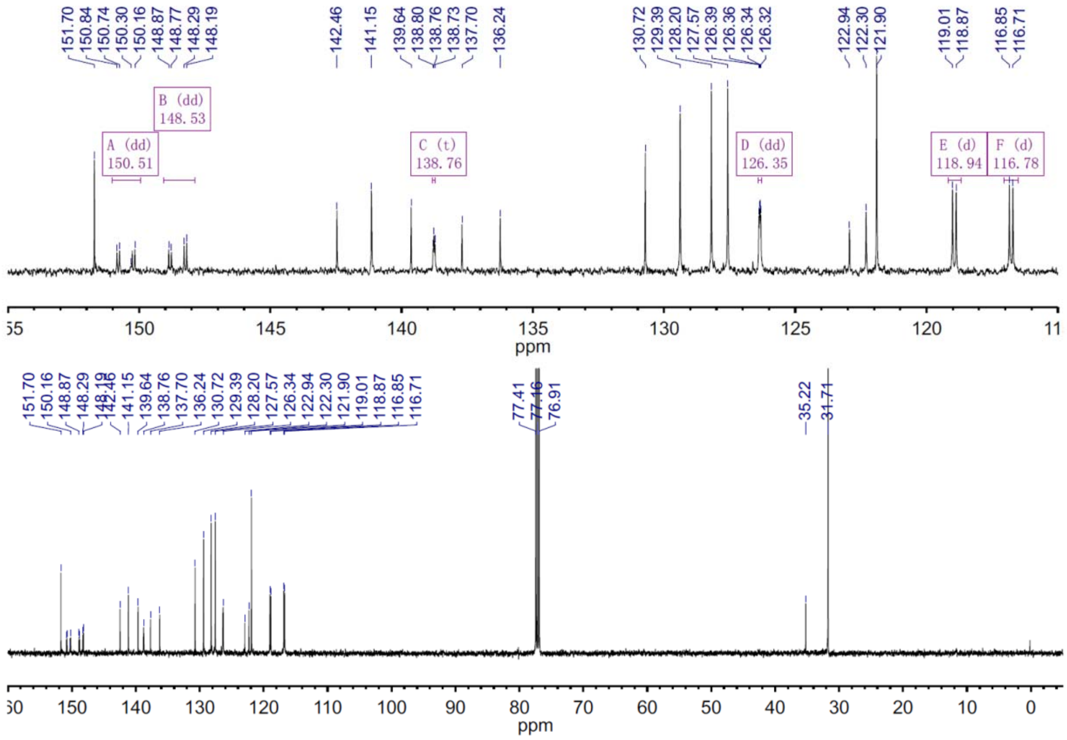

Supplementary Figure 39 | ^{13}C NMR spectrum of 10f.

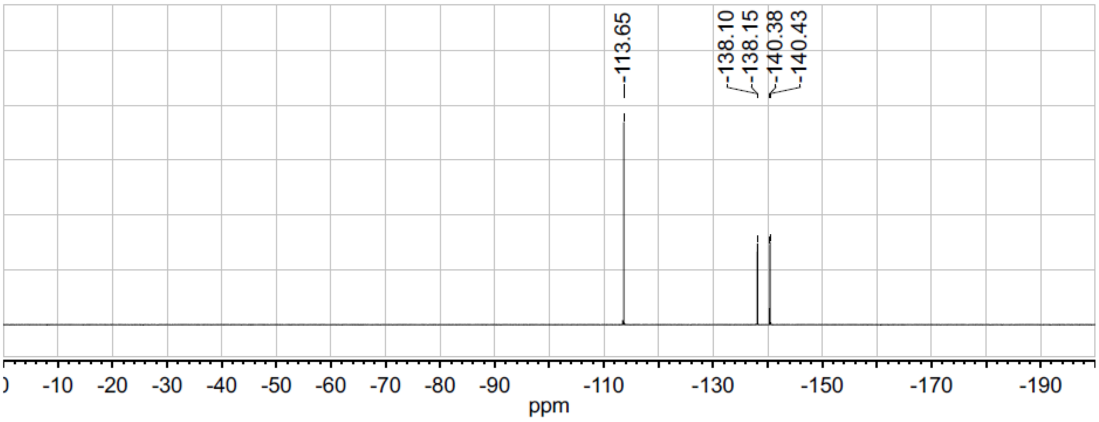

Supplementary Figure 40 | ^{19}F NMR spectrum of 10f.

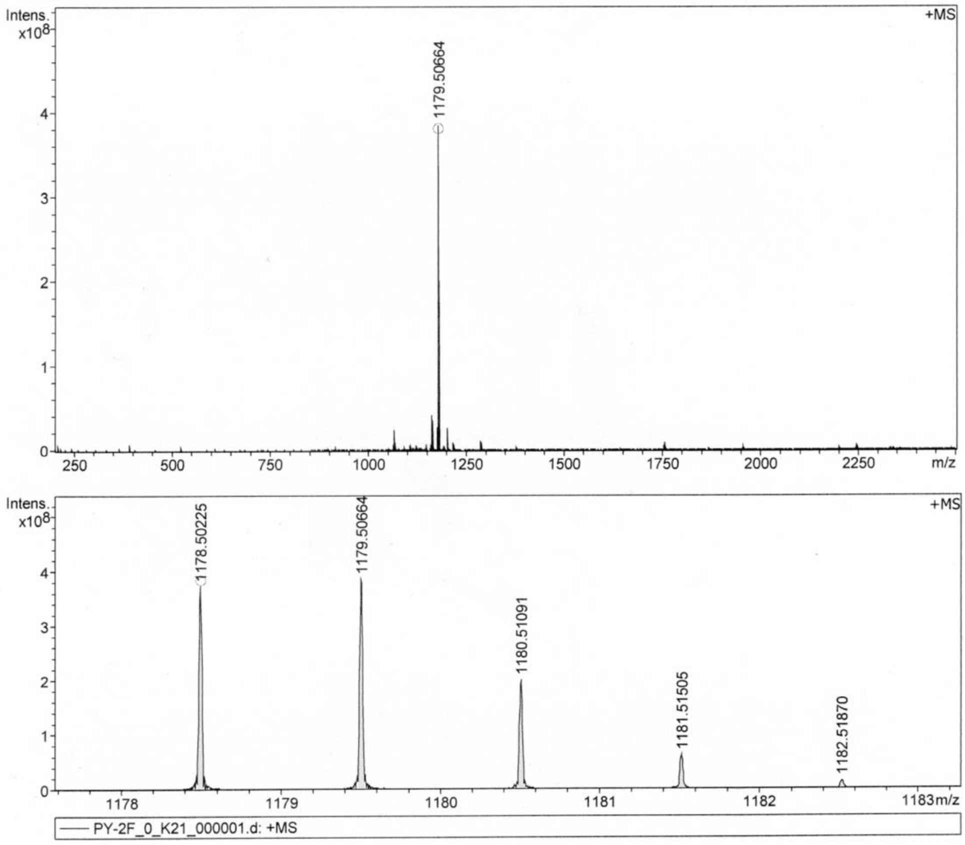

Meas. m/z	#	Ion Formula	Score	m/z	err [ppm]	Mean err [ppm]	mSigma	rdb	e⁻ Conf	N-Rule
1178.502255	1	C80H66F8	100.00	1178.503129	0.7	-0.1	83.0	44.0	odd	ok

Supplementary Figure 41 | Positive mode MALDI TOF mass spectrum of 10f.

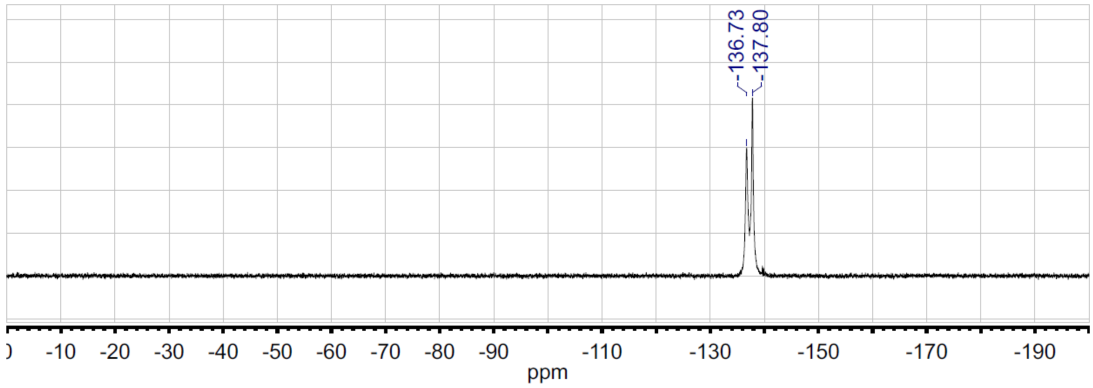

Supplementary Figure 42 | ^{19}F NMR spectrum of 1f.

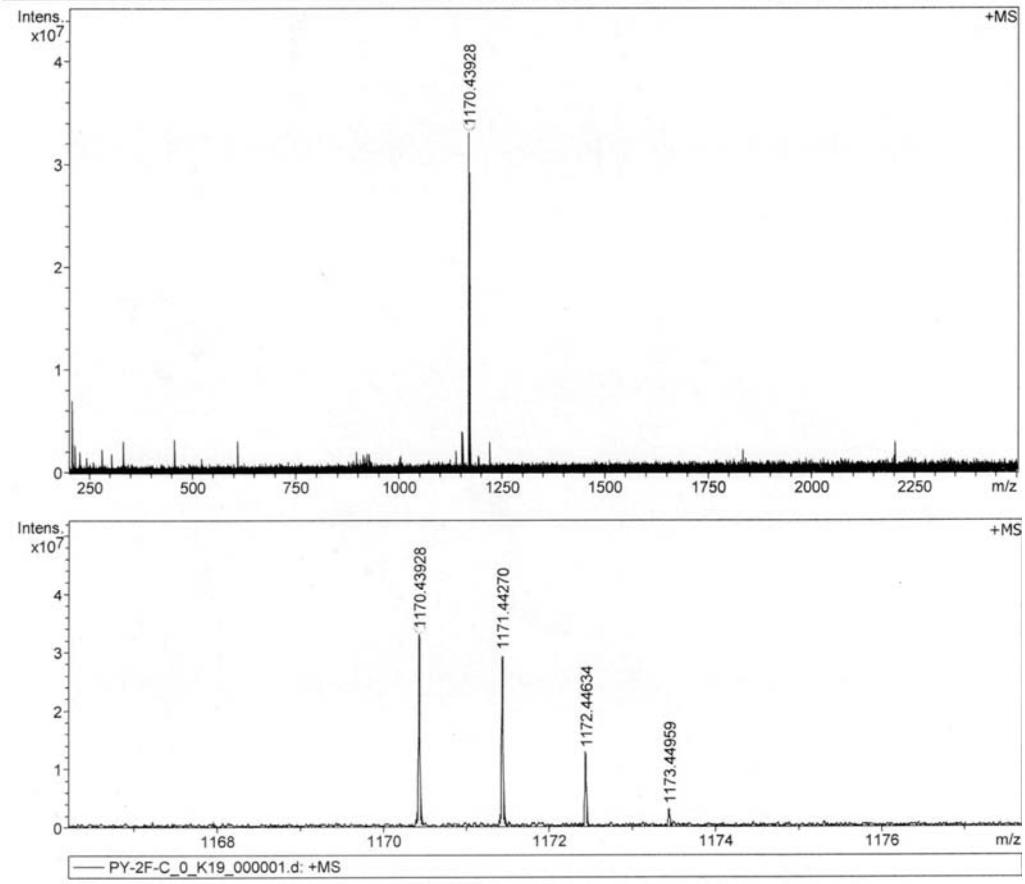

Supplementary Figure 43 | Positive mode MALDI TOF mass spectrum of 1f.

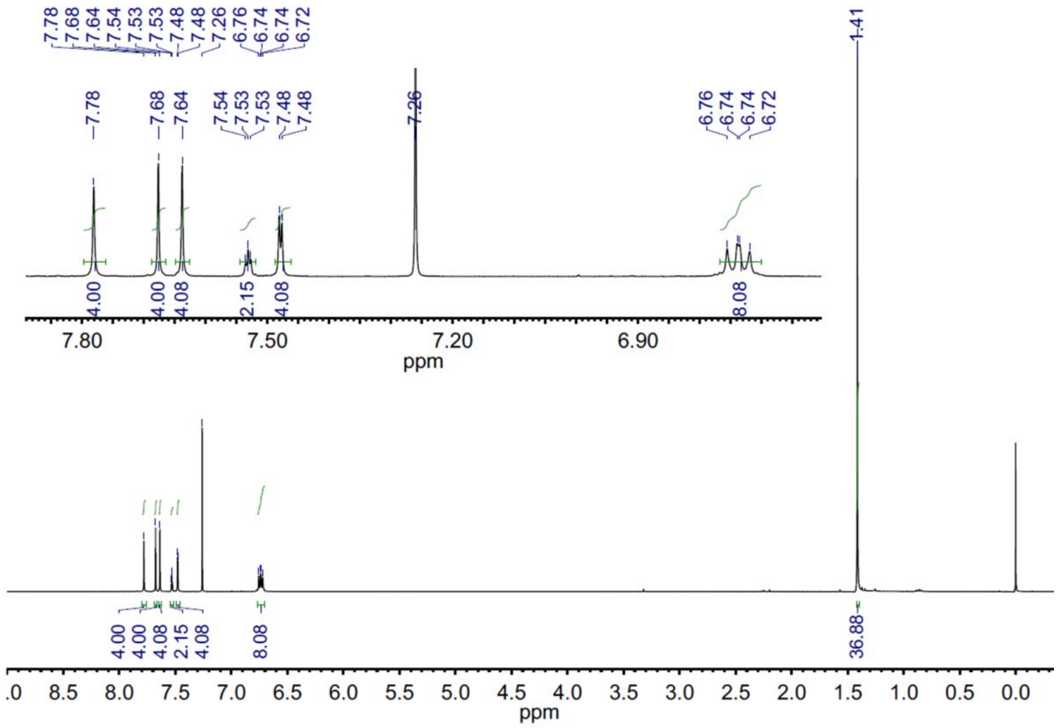

Supplementary Figure 44 | ^{1}H NMR spectrum of 10g.

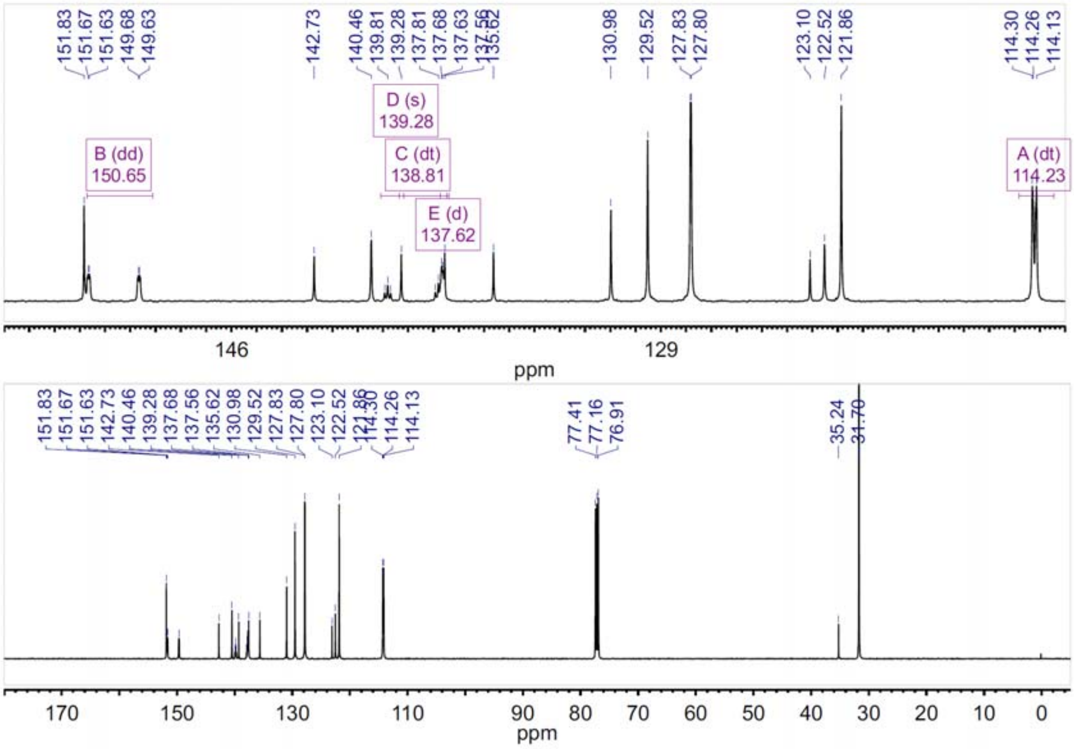

Supplementary Figure 45 | ^{13}C NMR spectrum of 10g.

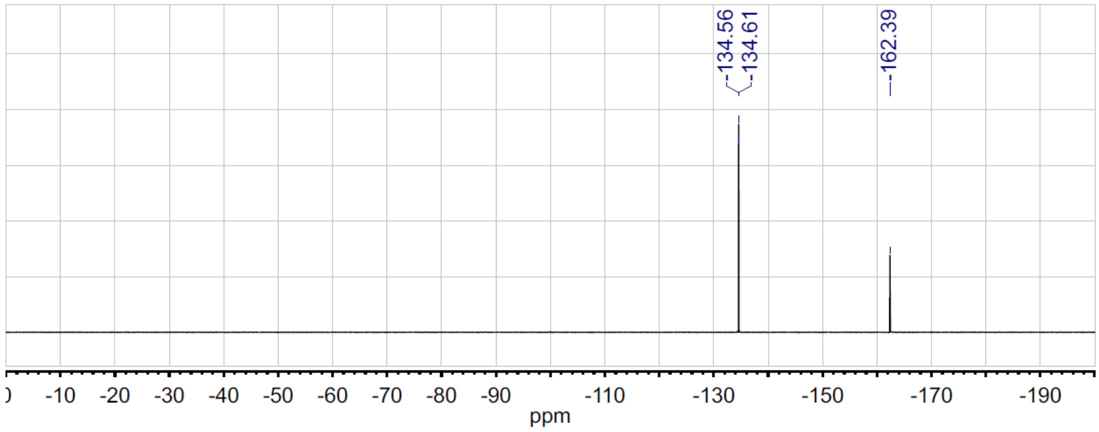

Supplementary Figure 46 | ^{19}F NMR spectrum of 10g.

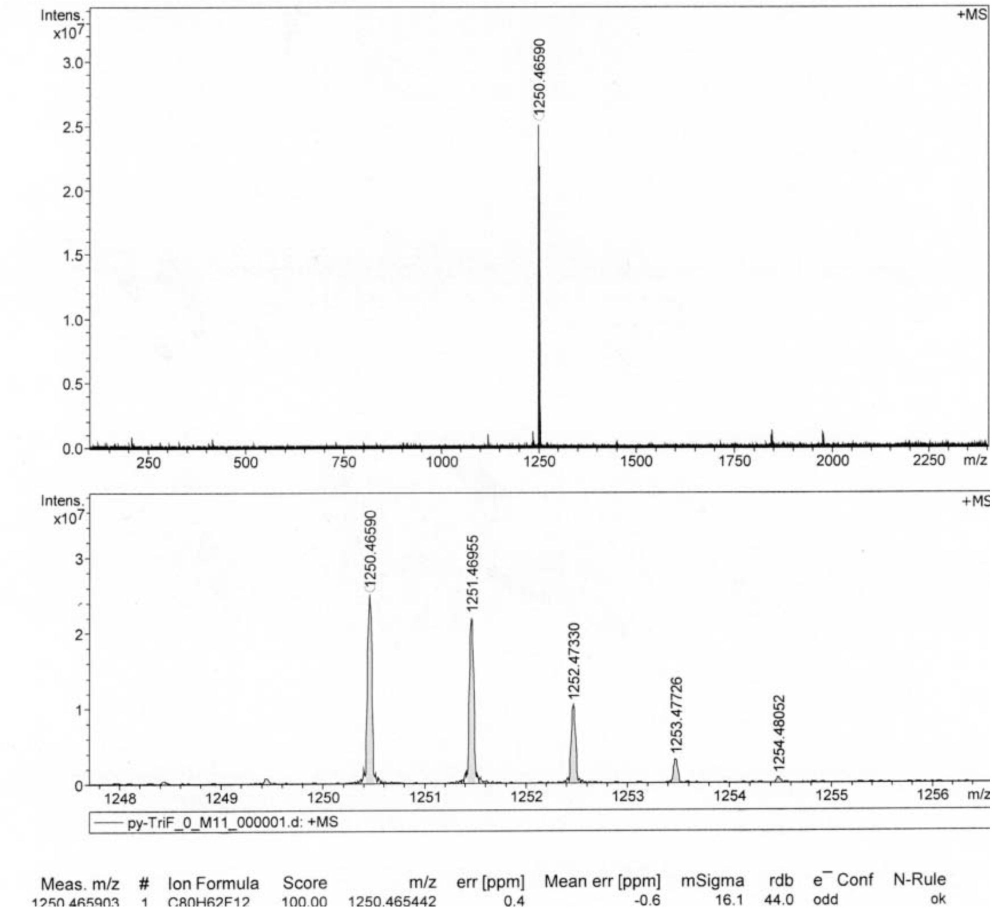

Supplementary Figure 47 | Positive mode MALDI TOF mass spectrum of 10g.

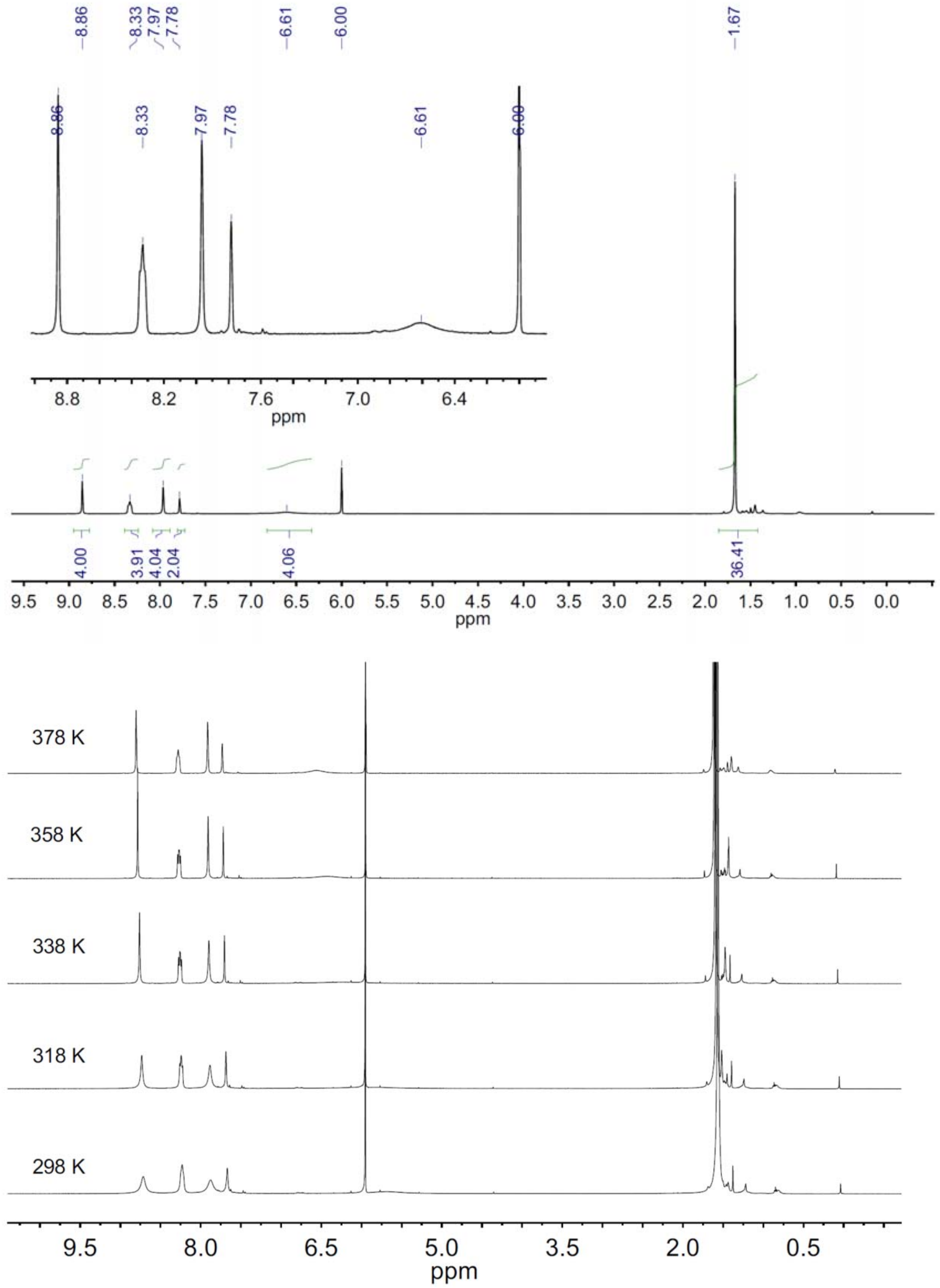

Supplementary Figure 48 | ^{1}H NMR and variable temperature ^{1}H NMR spectra of 1g.

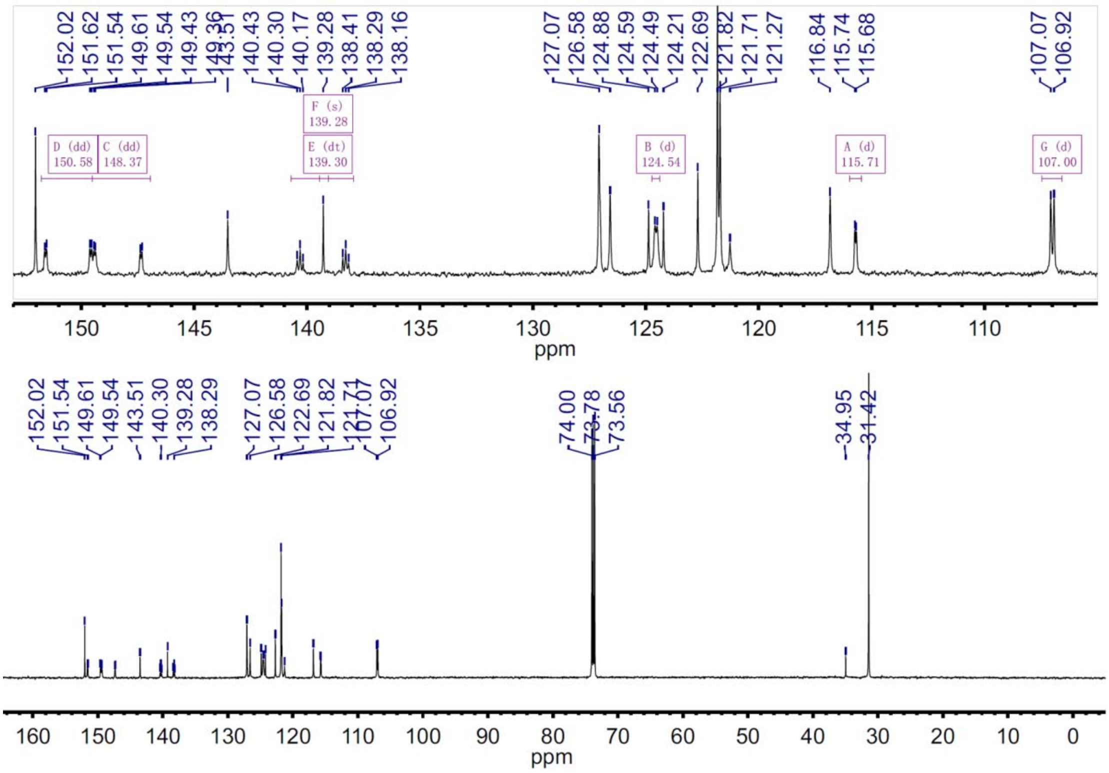

Supplementary Figure 49 | ^{13}C NMR spectra of 1g.

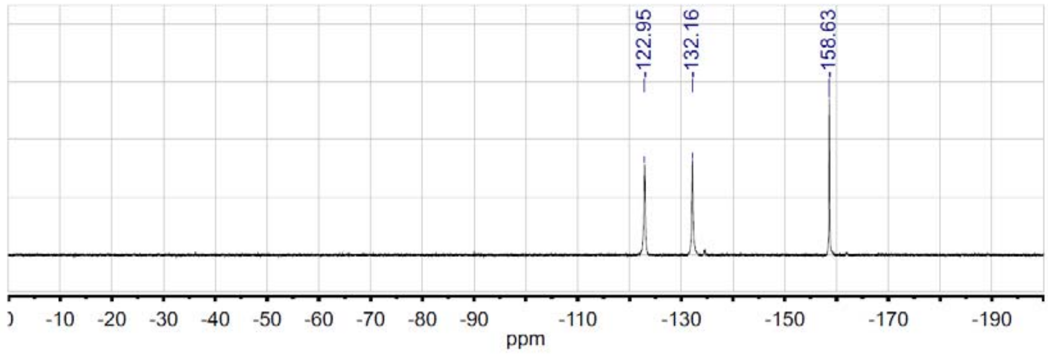

Supplementary Figure 50 | ^{19}F NMR spectrum of 1g.

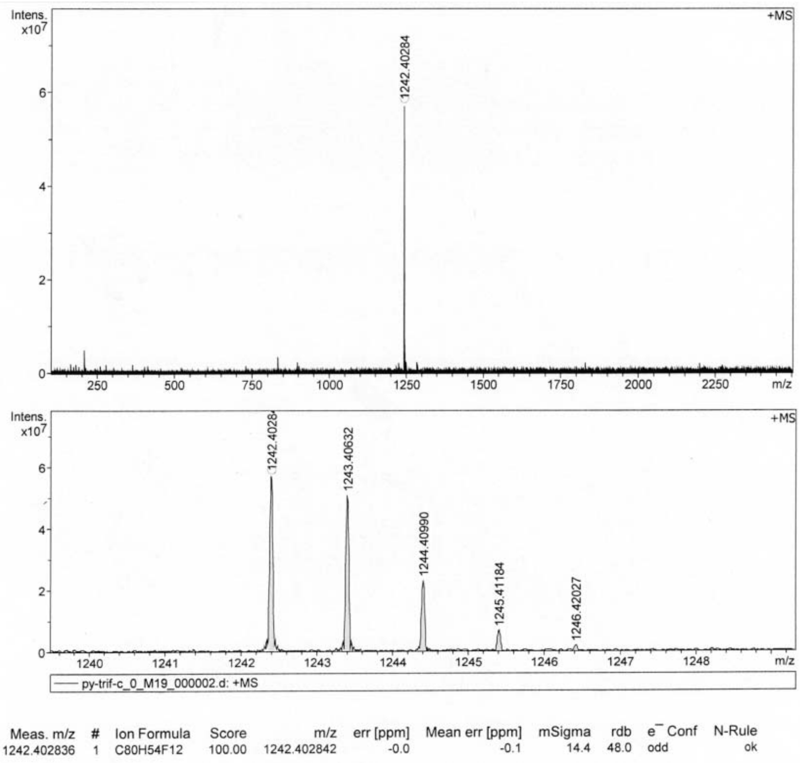

Supplementary Figure 51 | Positive mode MALDI TOF mass spectrum of 1g.

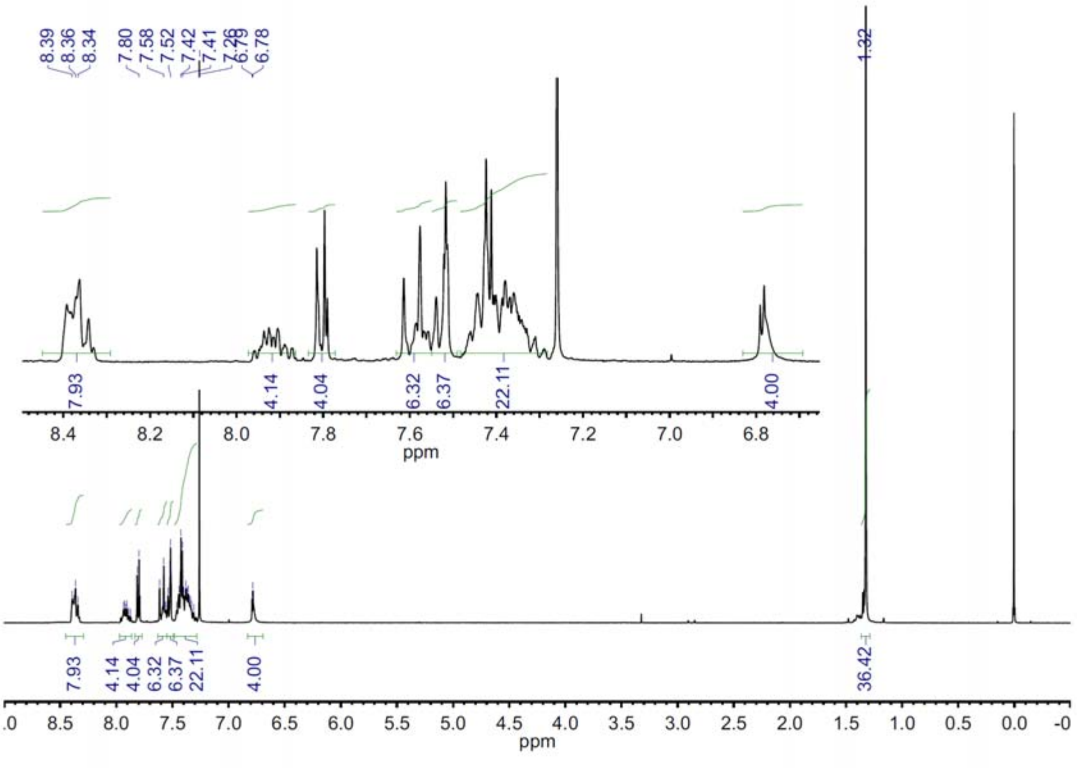

Supplementary Figure 52 | ^{1}H NMR spectrum of 11.

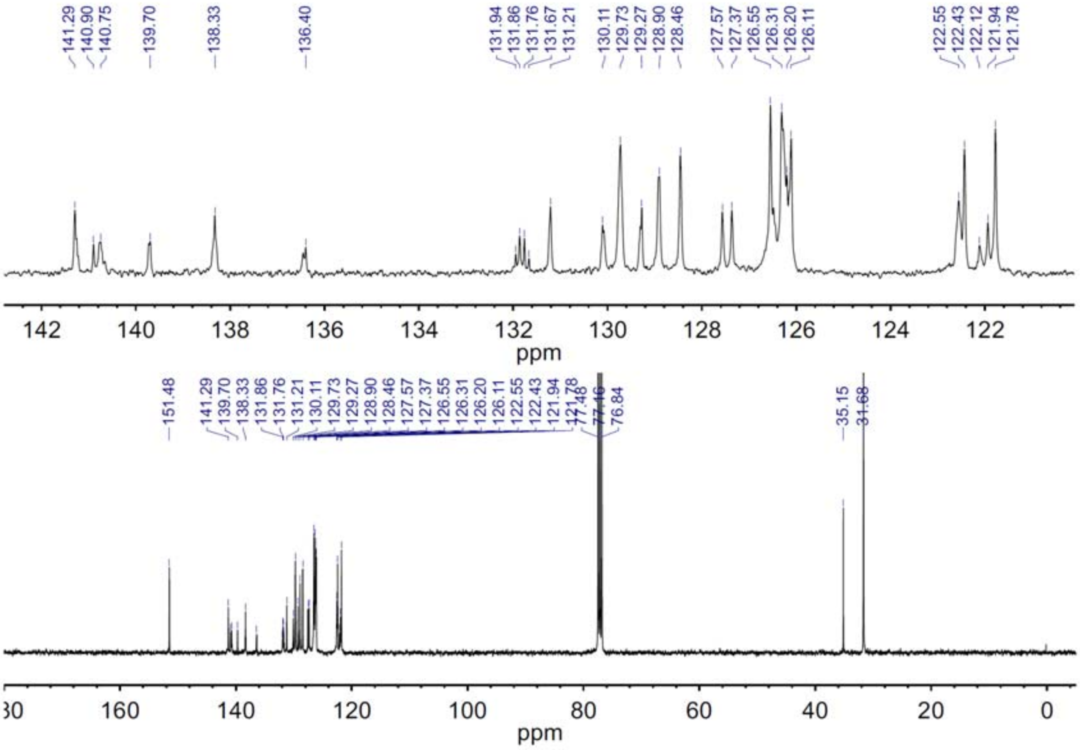

Supplementary Figure 53 | ^{13}C NMR spectrum of 11.

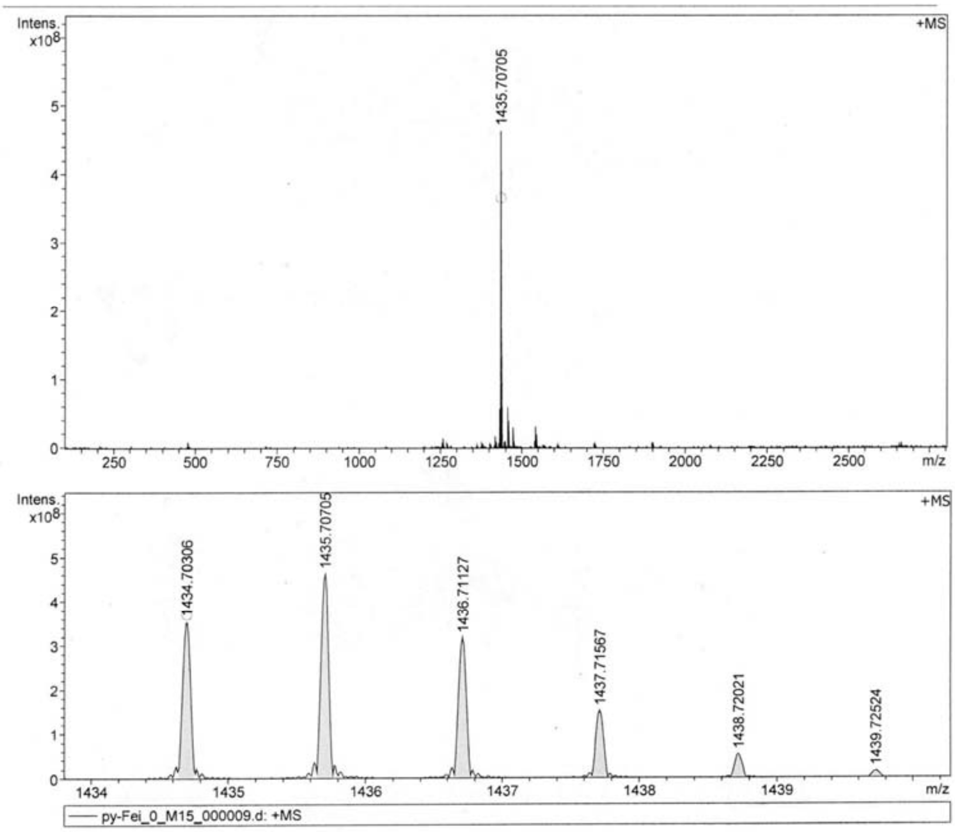

Meas. m/z	#	Ion Formula	Score	m/z	err [ppm]	Mean err [ppm]	mSigma	rdb	e⁻ Conf	N-Rule
1434.703060	1	C112H90	100.00	1434.703704	-0.4	-0.3	55.9	68.0	odd	ok

Supplementary Figure 54 | Positive mode MALDI TOF mass spectrum of 11.

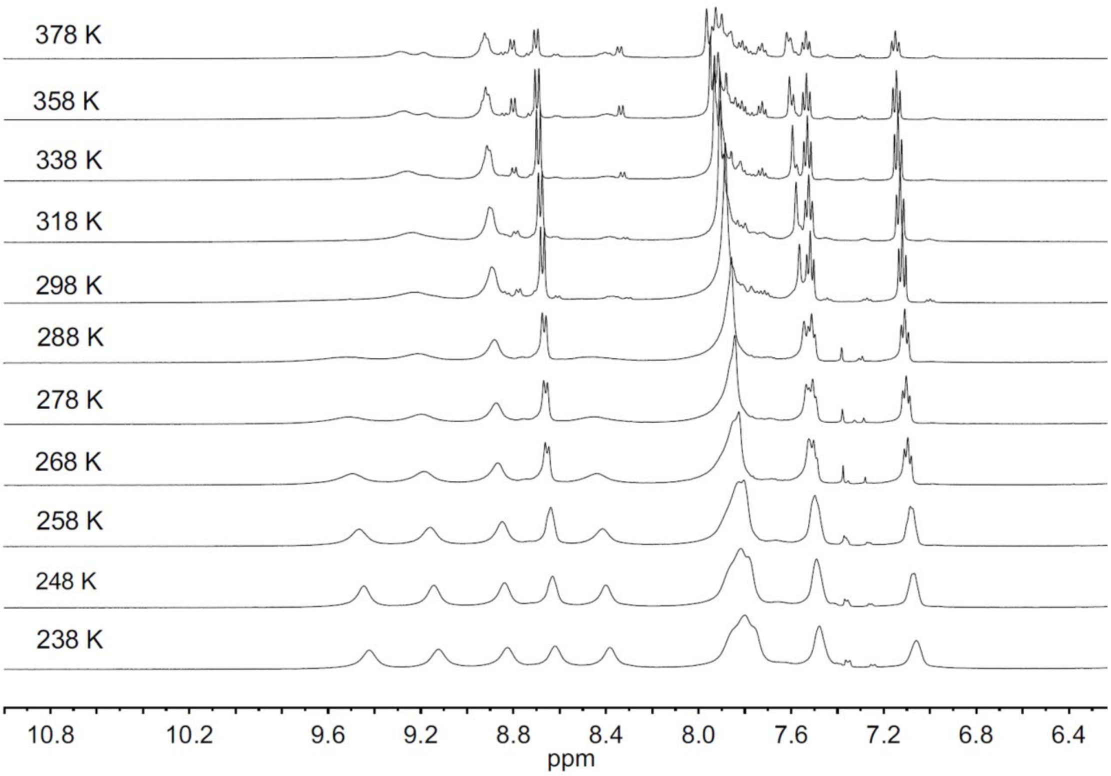

Supplementary Figure 55 | VT-^{1}H NMR spectra of 2.

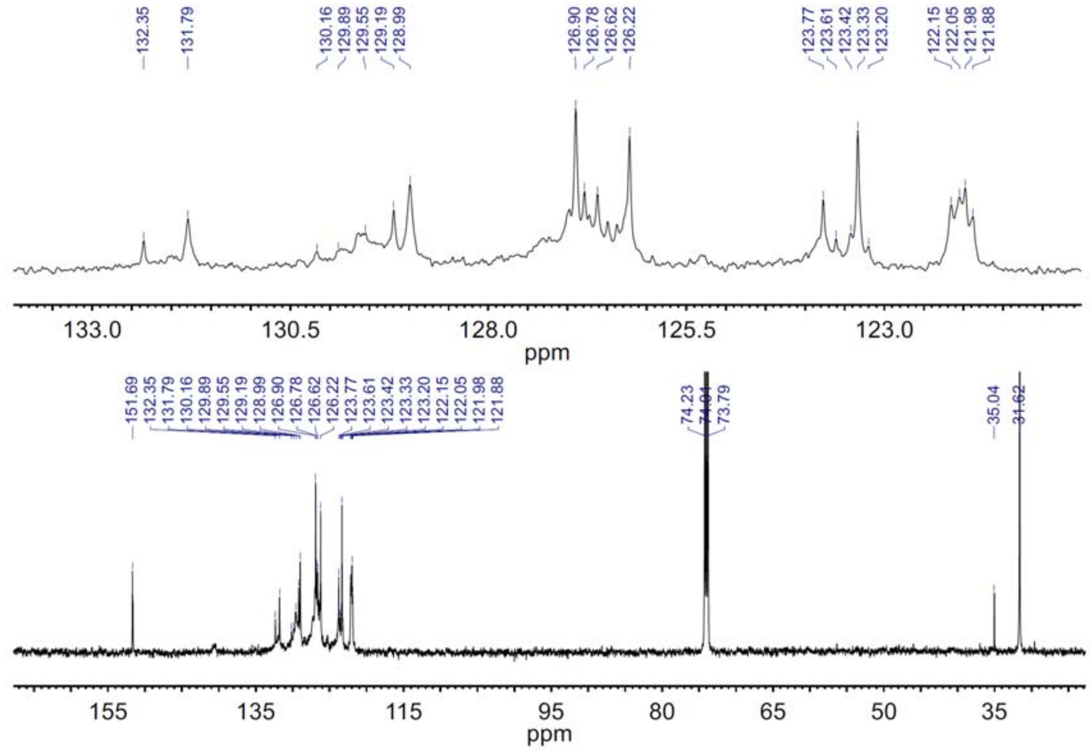

Supplementary Figure 56 | ^{13}C NMR spectrum of 2.

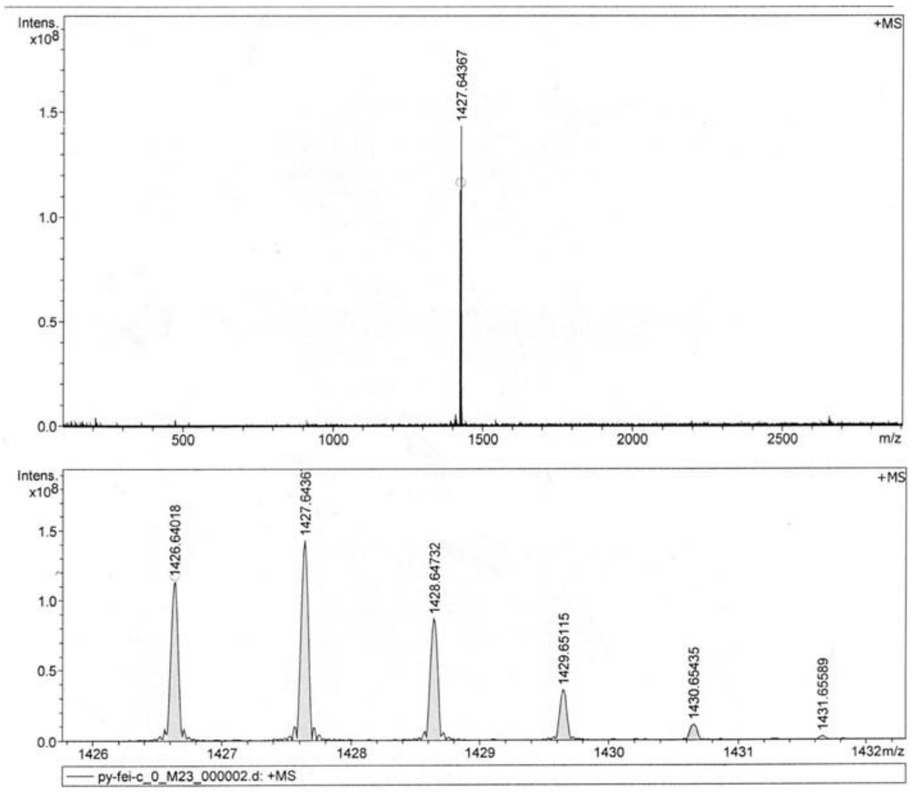

Meas. m/z	#	Ion Formula	Score	m/z	err [ppm]	Mean err [ppm]	mSigma	rdb	e⁻ Conf	N-Rule
1426.640184	1	C112H82	100.00	1426.641104	-0.6	0.5	9.6	72.0	odd	ok

Supplementary Figure 57 | Positive mode MALDI TOF mass spectrum of 2.

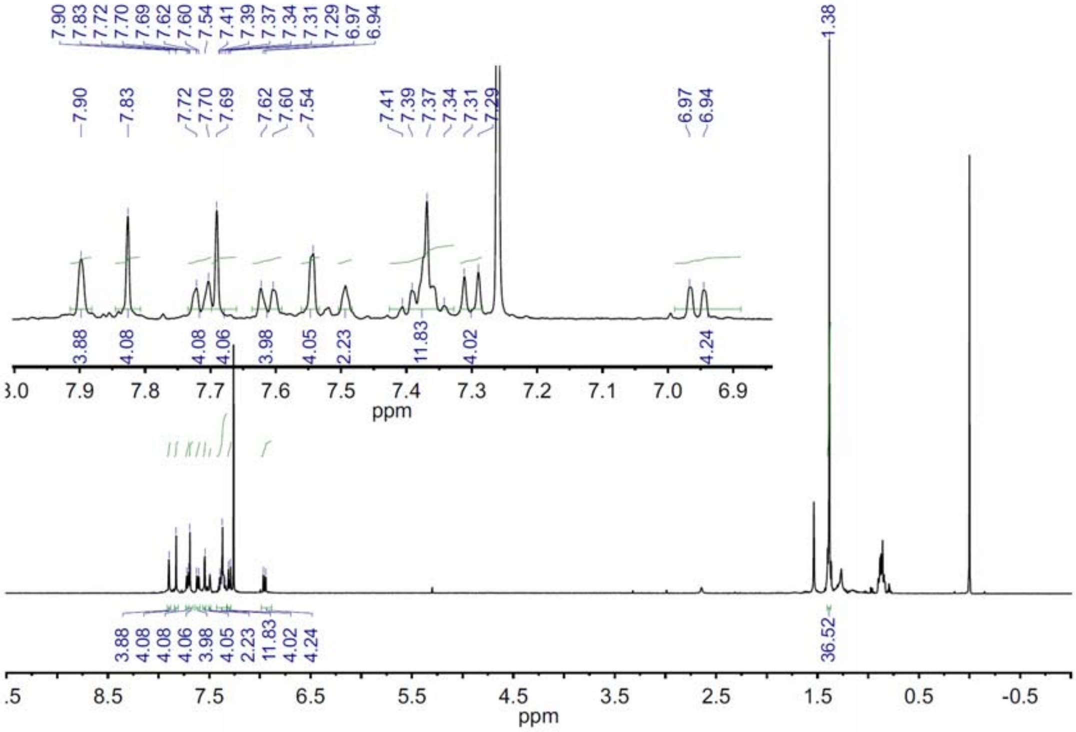

Supplementary Figure 58 | ^{1}H NMR spectrum of 12.

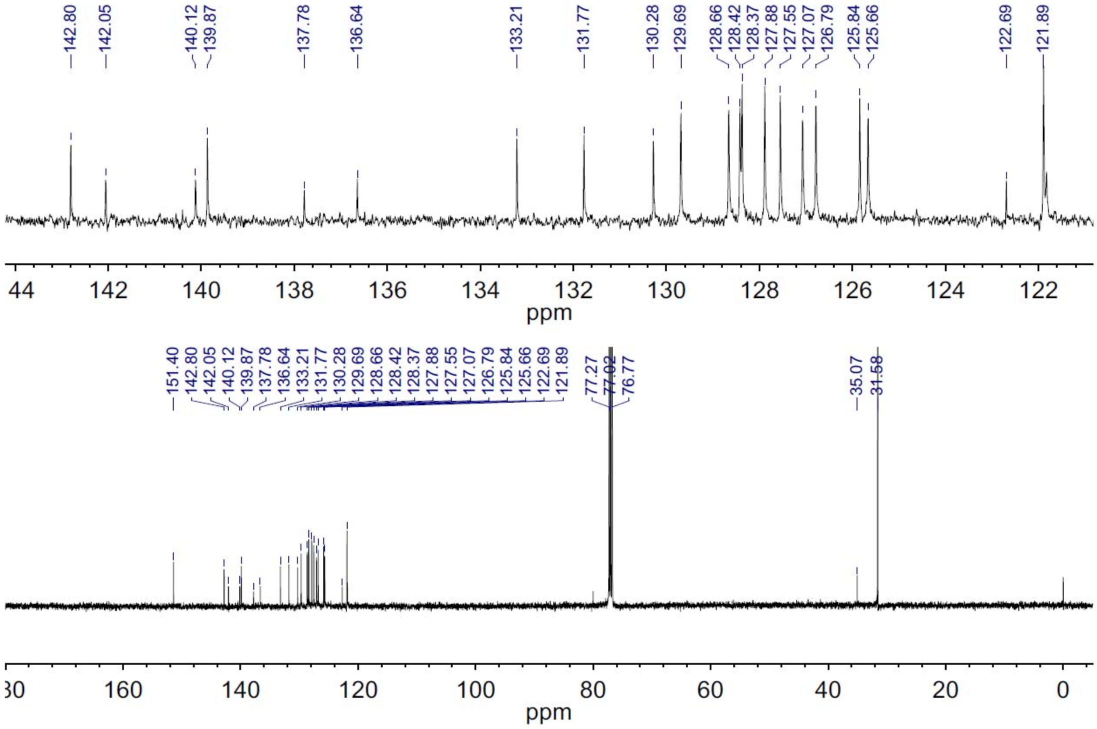

Supplementary Figure 59 | ^{13}C NMR spectrum of 12.

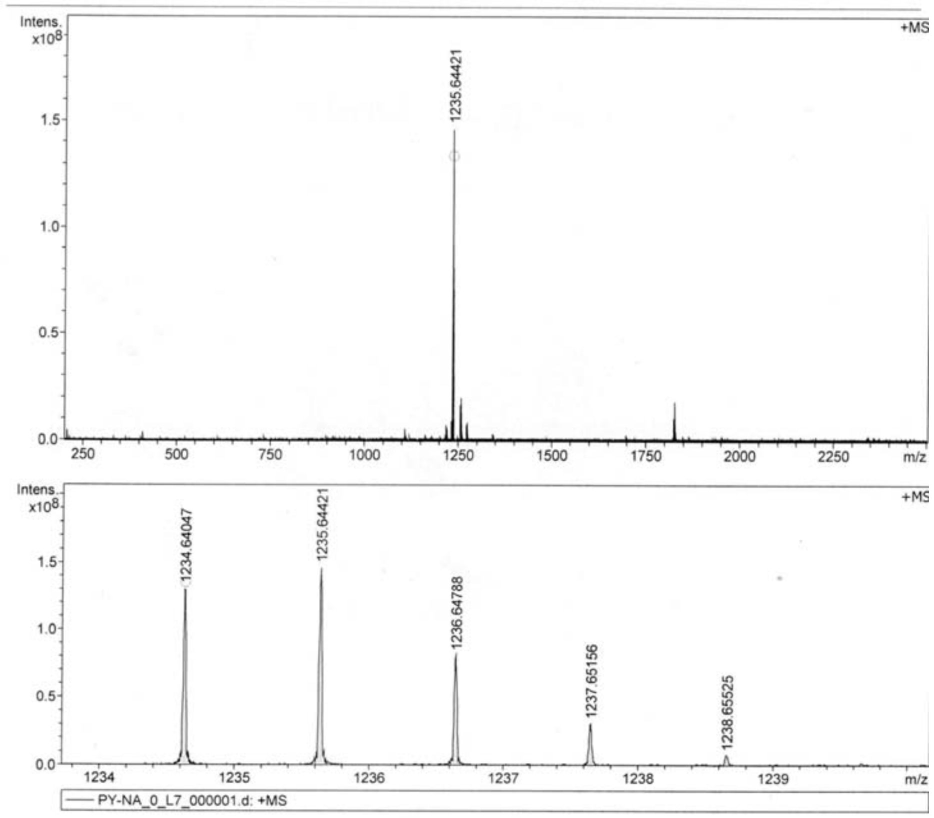

Meas. m/z	#	Ion Formula	Score	m/z	err [ppm]	Mean err [ppm]	mSigma	rdb	e⁻ Conf	N-Rule
1234.640474	1	C96H82	100.00	1234.641104	0.5	0.2	32.3	56.0	odd	ok

Supplementary Figure 60 | Positive mode MALDI TOF mass spectrum of 12.

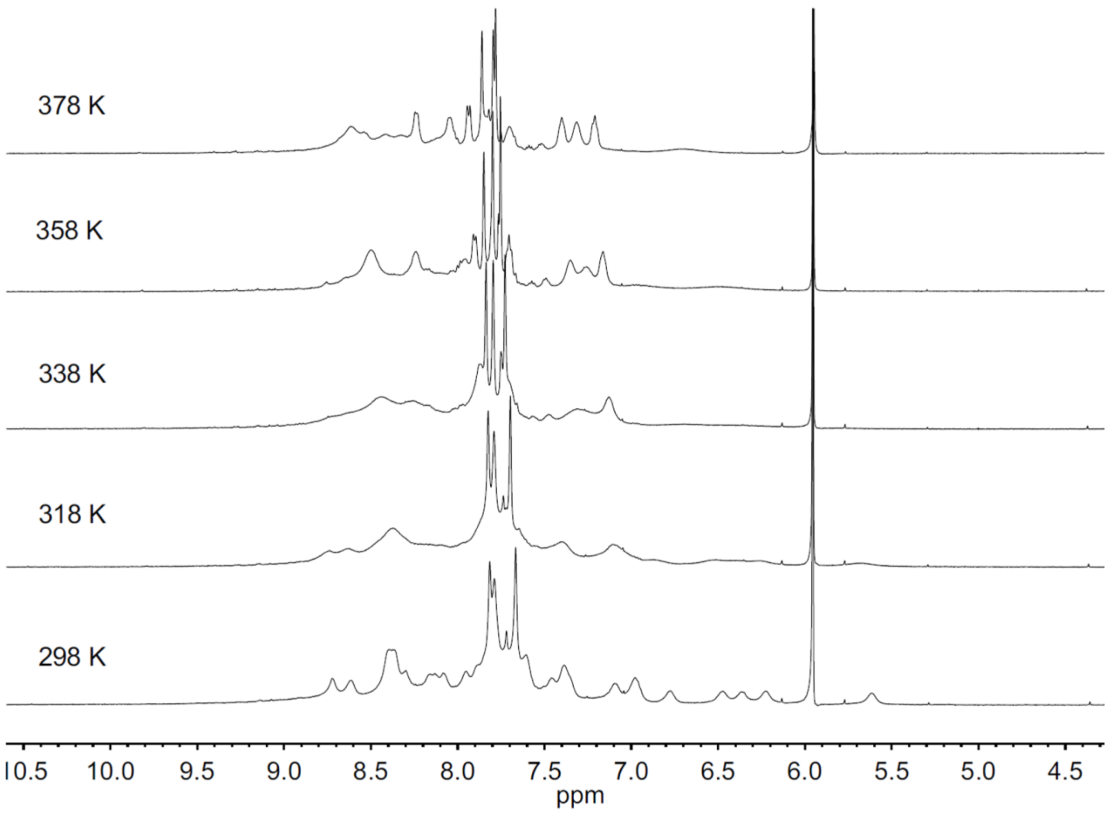

Supplementary Figure 61 | VT-^{1}H NMR spectra of 3.

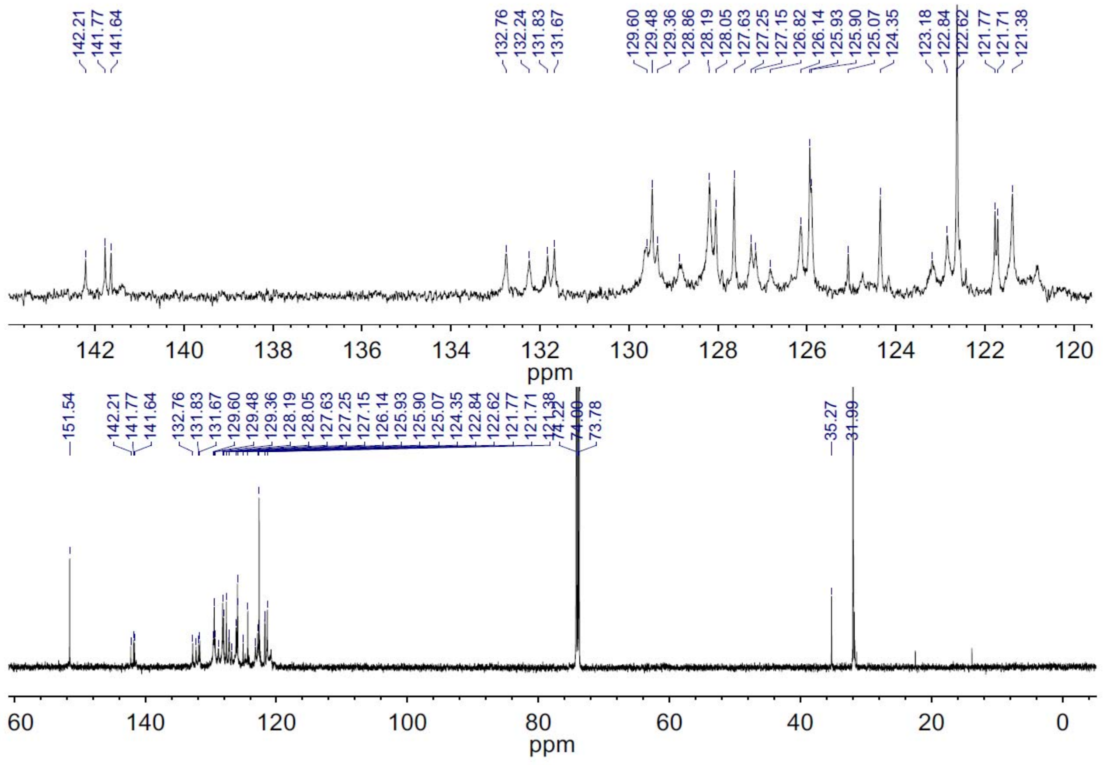

Supplementary Figure 62 | ^{13}C NMR spectrum of 3.

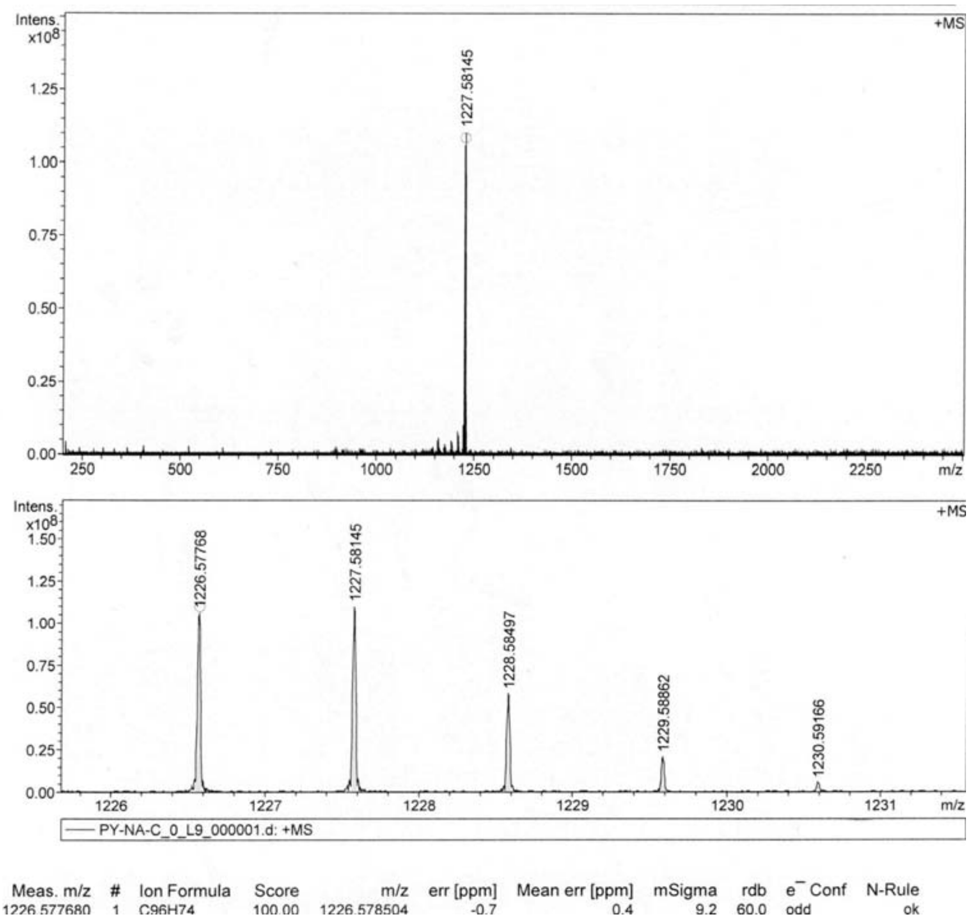

Supplementary Figure 63 | Positive mode MALDI TOF mass spectrum of 3.

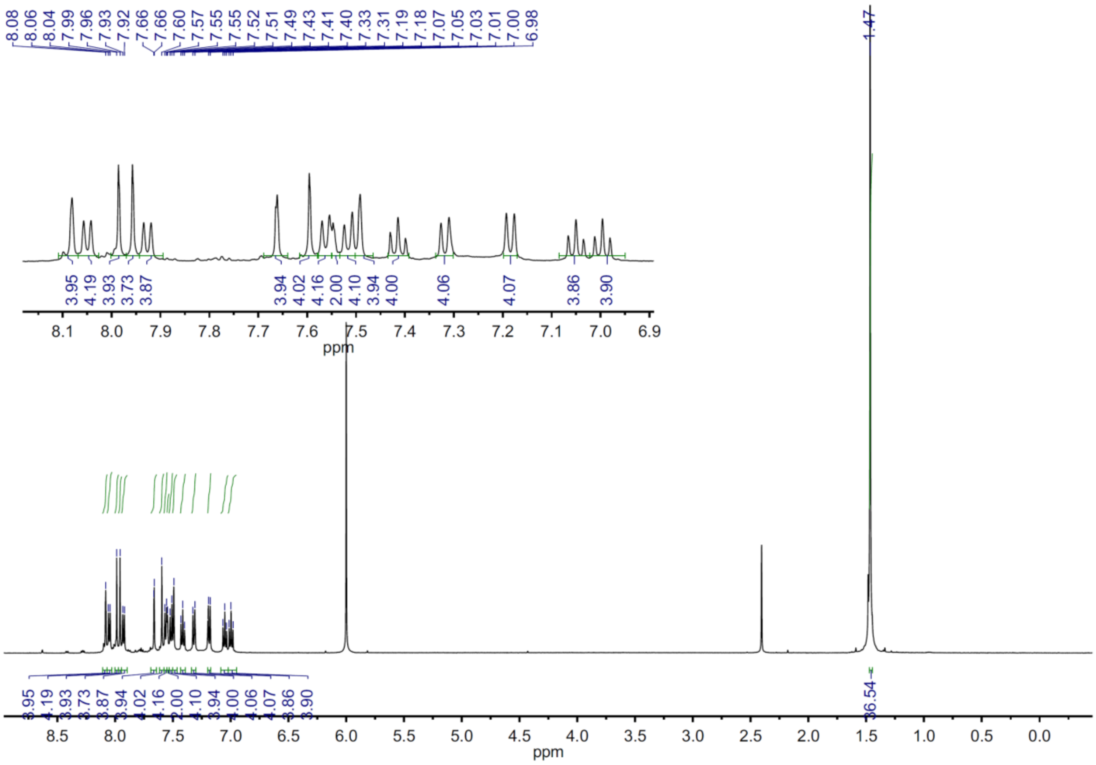

Supplementary Figure 64 | ^{1}H NMR spectrum of 13.

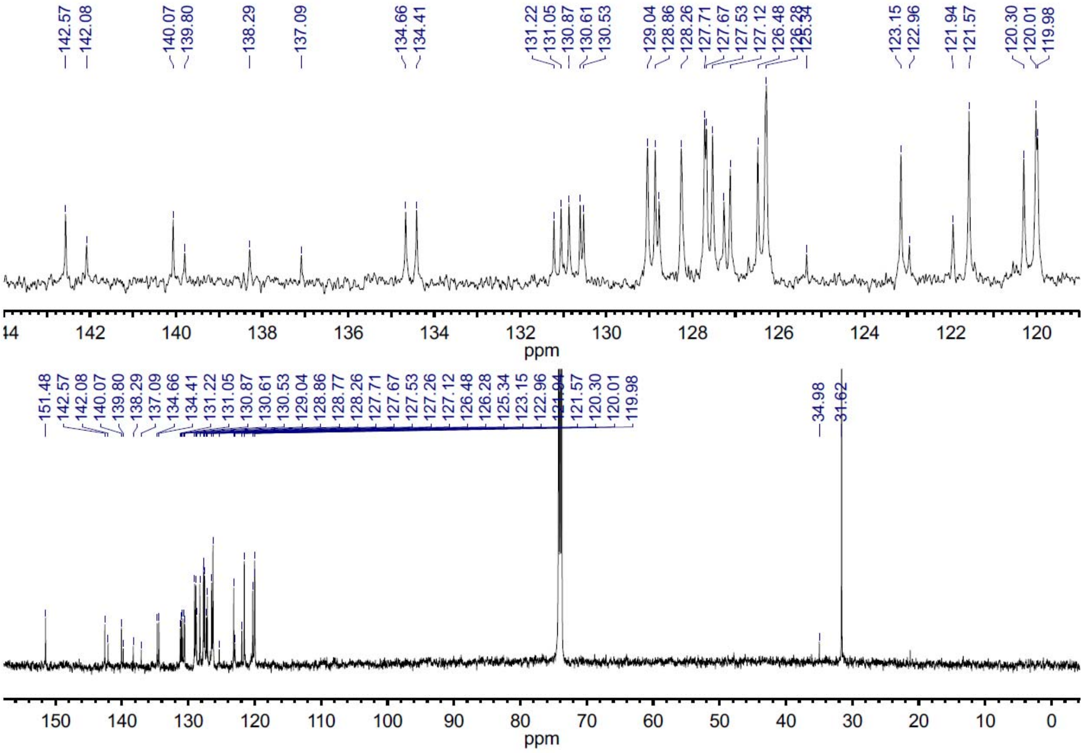

Supplementary Figure 65 | ^{13}C NMR spectrum of 13.

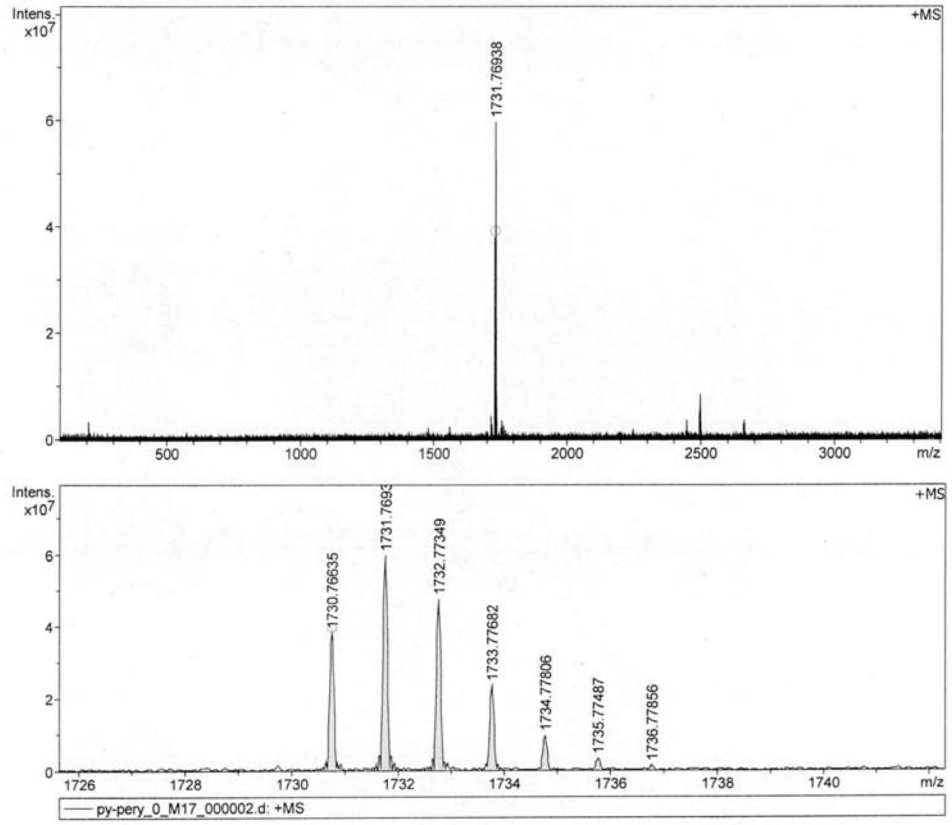

Meas. m/z	#	Ion Formula	Score	m/z	err [ppm]	Mean err [ppm]	mSigma	rdb	e^- Conf	N-Rule
1730.766348	1	C136H98	100.00	1730.766305	0.0	0.1	33.2	88.0	odd	ok

Supplementary Figure 66 | Positive mode MALDI TOF mass spectrum of 13.

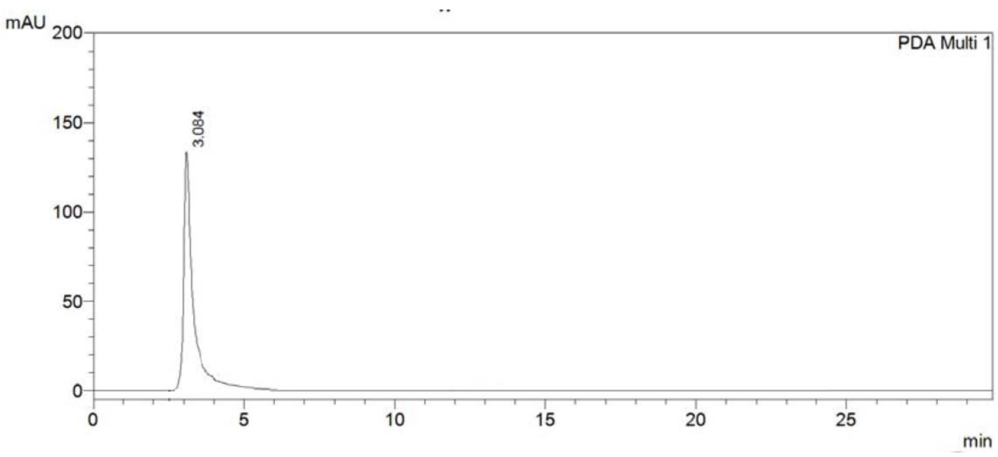

Supplementary Figure 67 | Representative HPLC curve of compound 4. Shim-Pack PREP-SIL column (250mm × 20 mm), THF/MeOH = 90/10 as eluent, flow rate 1 mL·min^{-1}, and detection wavelength is 575 nm.

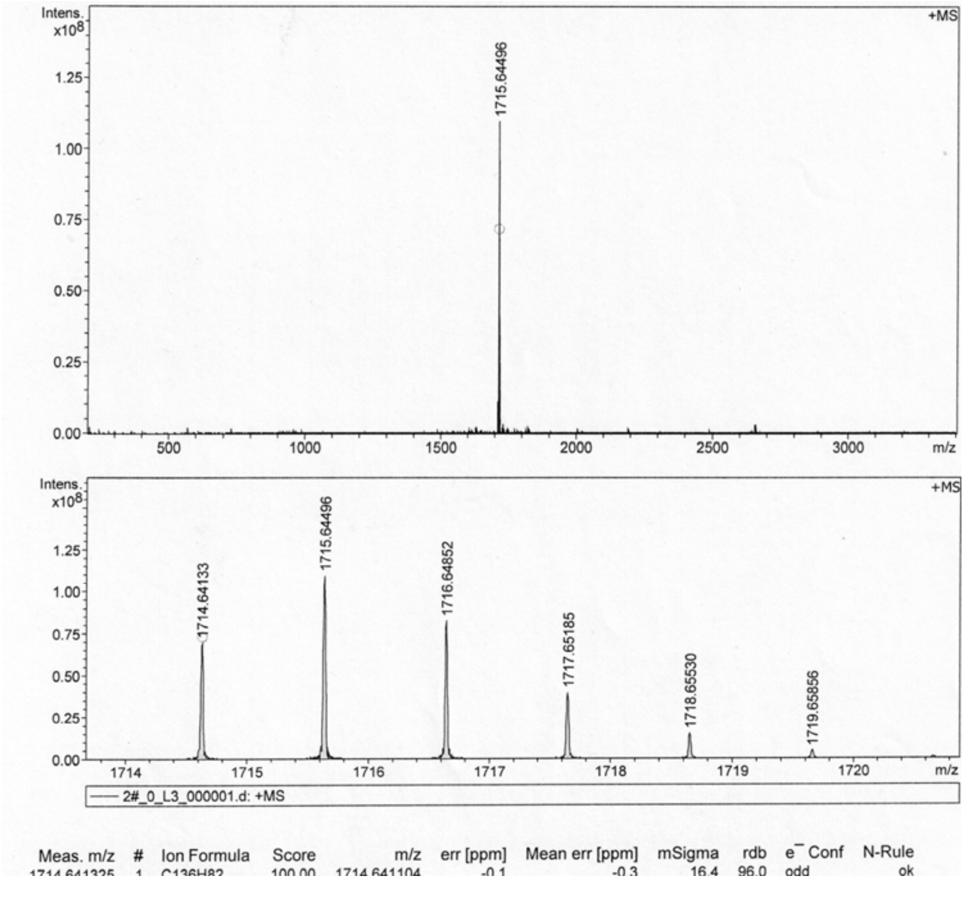

Supplementary Figure 68 | Positive mode MALDI TOF mass spectrum of 4.

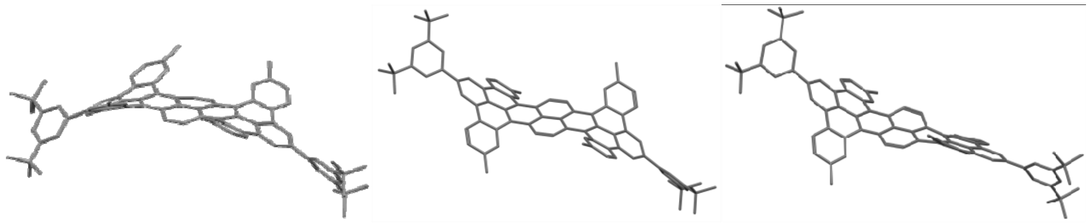

*s*AA *t*AA AT

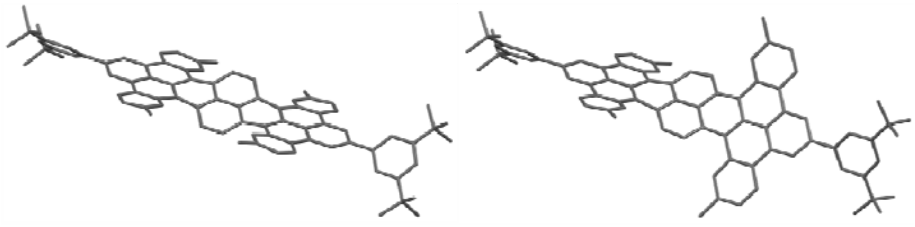

sTT*t*TT

Supplementary Figure 69 | Five possible conformations of 1a.

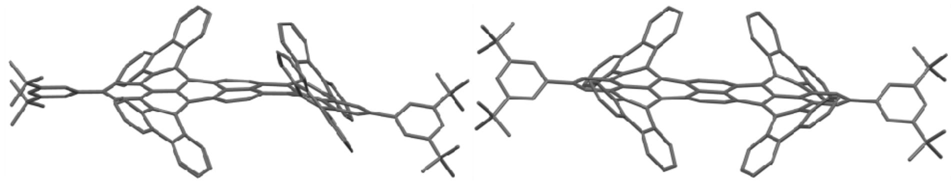

AT *t*TT

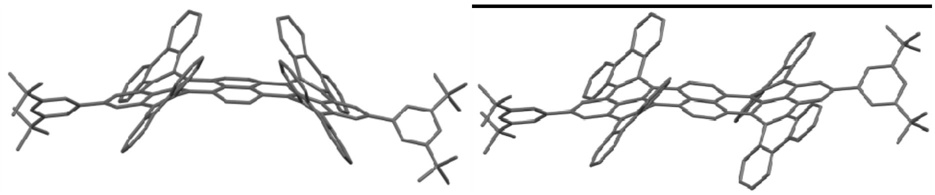

*s*AA *t*AA

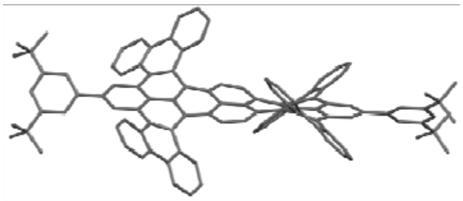

sTT

Supplementary Figure 70 | Five possible conformations of 2.

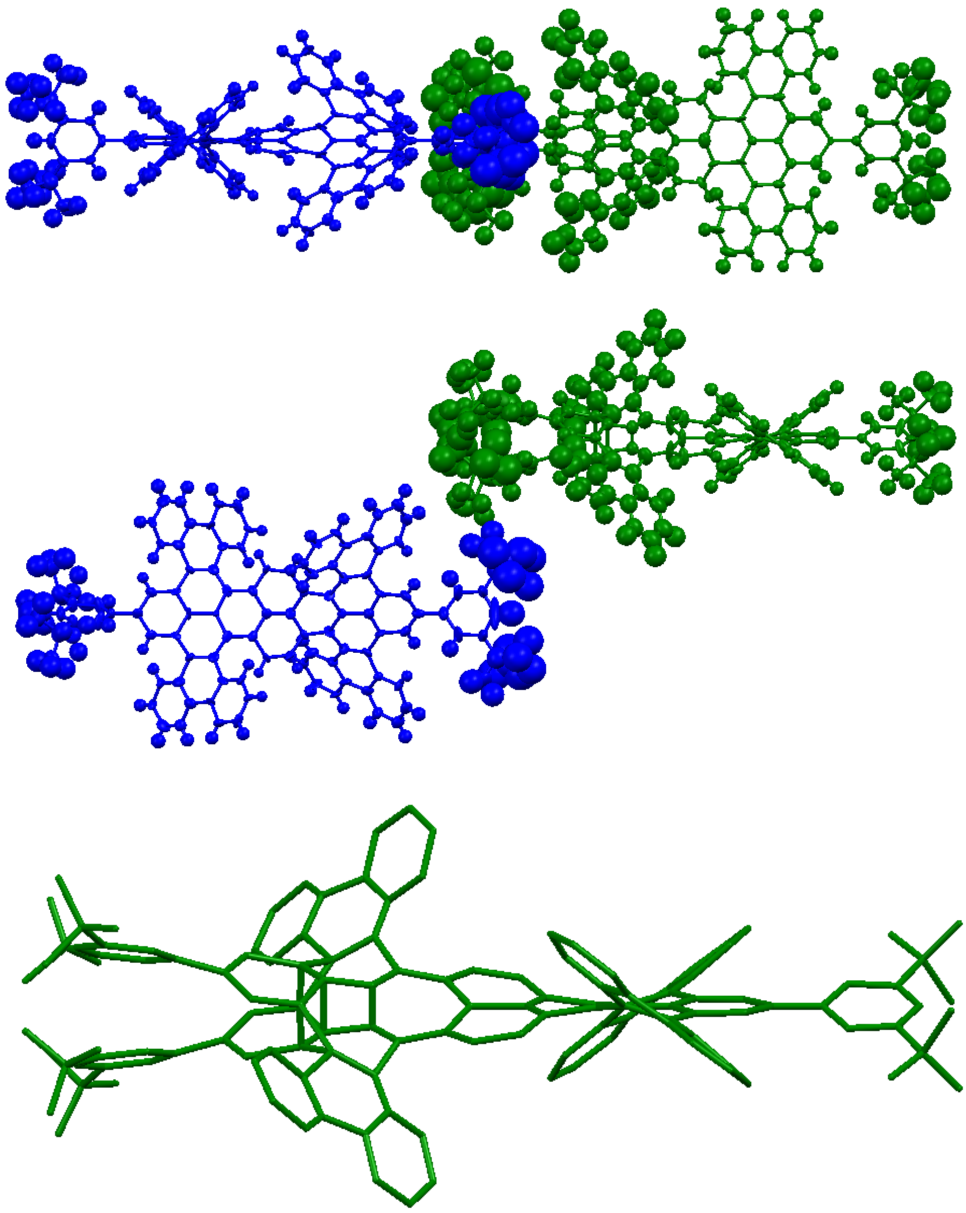

Supplementary Figure 71 | Crystal structure of 2. Top: along b axis (ellipsoid at 50% level); middle: based on the top figure, then flip by 90 degree around the horizontal axis (ellipsoid at 50% level); down: strong disorder for half of the molecule (hydrogen atoms were omitted).

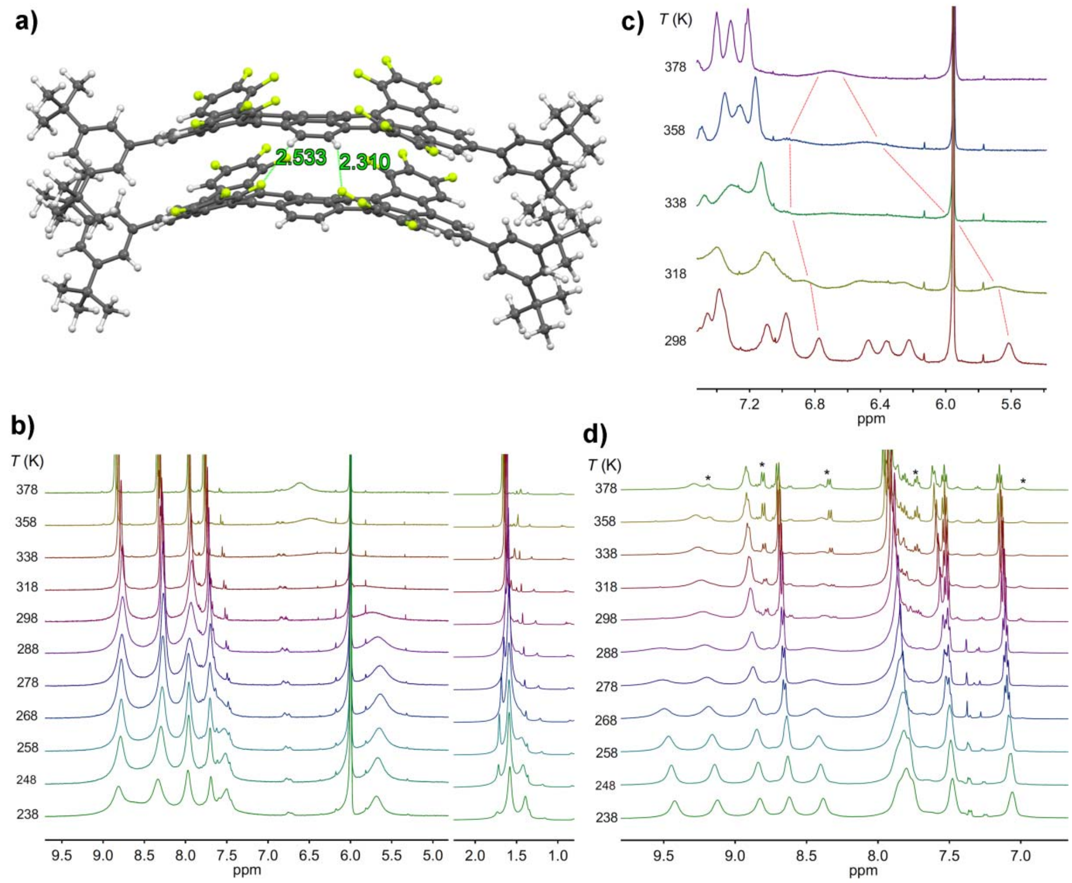

Supplementary Figure 72 │ Conformational flexibility of the 2D acenes in solution. (**a**) Optimized molecular structure of the **1g** dimer at the HF/3-21G level, with hydrogen atomsof the central benzo group of one molecule hydrogen bonding with fluorine atoms of the other. (**b**)Variable-temperature ^{1}H NMR spectra of **1g** (500 MHz, $C_2D_2Cl_4$), revealing the transformation from monomers at high temperature to persistent π-dimers at low temperature. (**c**) Variable-temperature ^{1}H NMR spectra of **3** (500 MHz, $C_2D_2Cl_4$), revealing the freezing of five different conformers upon cooling. (**d**) Variable-temperature ^{1}H NMR spectra of *c*TT-**2** (500 MHz, $C_2D_2Cl_4$), revealing thermal isomerization at high temperature (coalescence of peaks marked with asterisks).

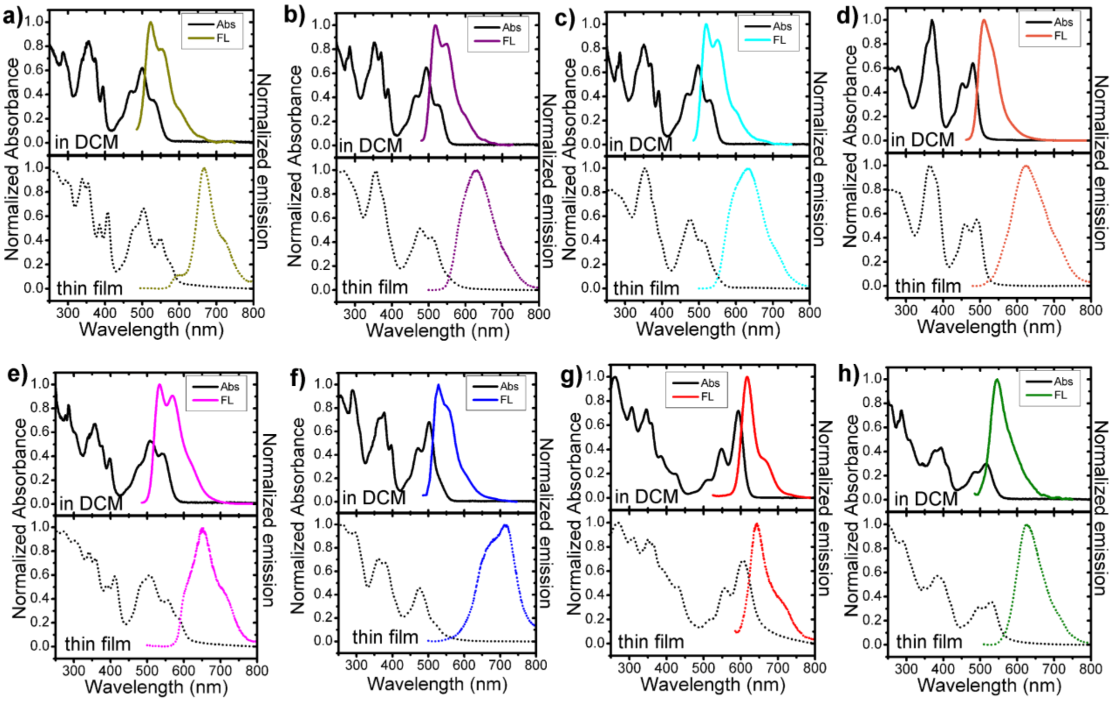

Supplementary Figure 73 | Solution and solid state optical properties of 1-3. Absorbance (black) and emission (colored) spectra of **a) 1a**, **b) 1e**, **c) 1f**, **d) 1g**, **e) 1b**, **f) 1c**, **g) 2**, and **h) 3** recorded in CH_2Cl_2 solution (solid lines) and in the solid state (spin-cast from CH_2Cl_2 solutions; dotted lines).

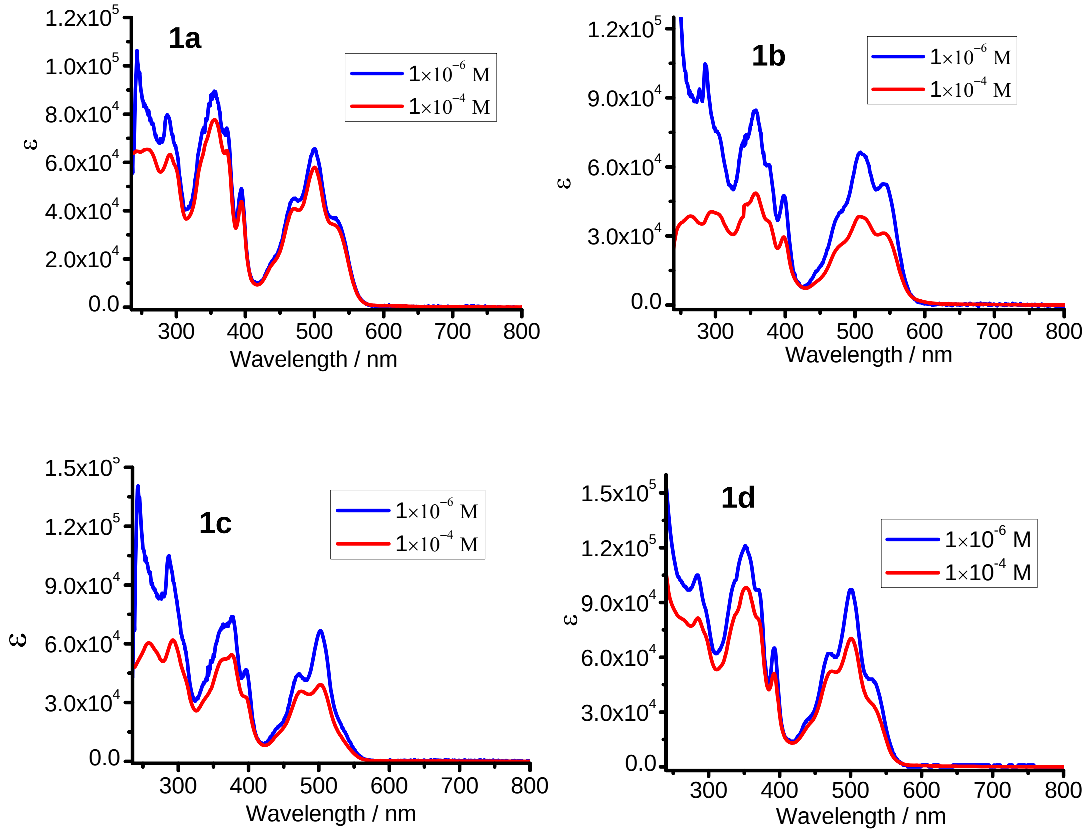

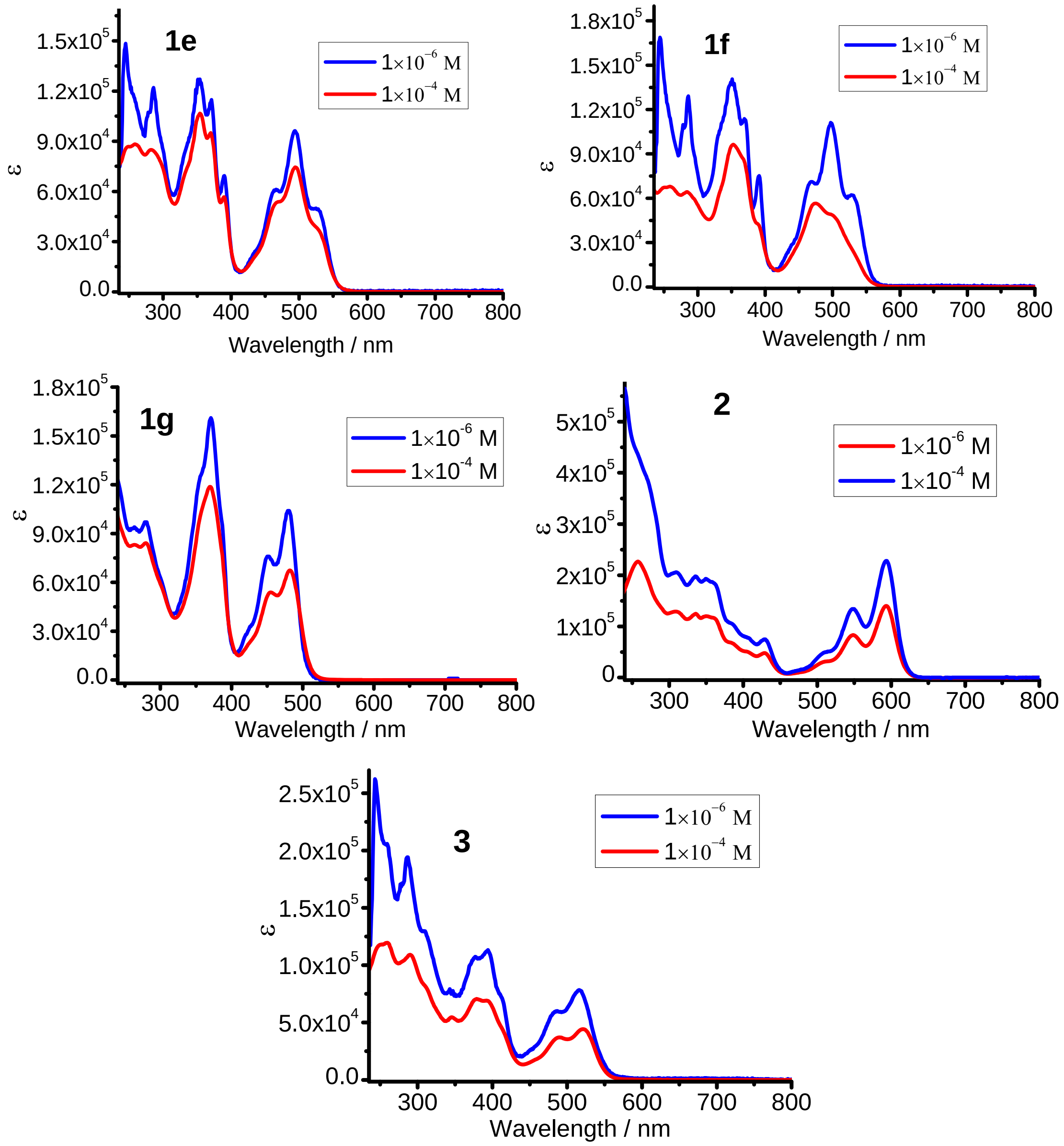

Supplementary Figure 74 | UV-vis spectra.1a, **1b**, **1c**, **1d**, **1e**, **1f**, **1g**, **2** and **3** at different concentrations in CH_2Cl_2.

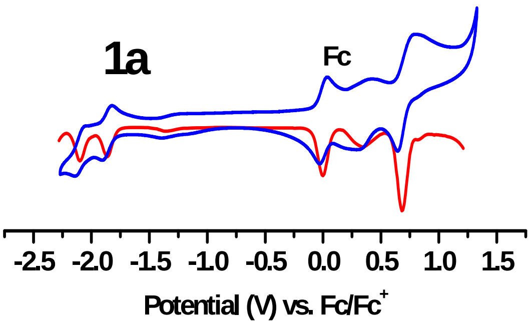
1a
Fc
-2.5 -2.0 -1.5 -1.0 -0.5 0.0 0.5 1.0 1.5
Potential (V) vs. Fc/Fc+

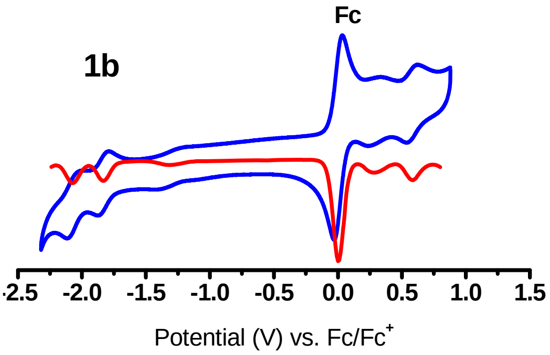
1b
Fc
-2.5 -2.0 -1.5 -1.0 -0.5 0.0 0.5 1.0 1.5
Potential (V) vs. Fc/Fc+

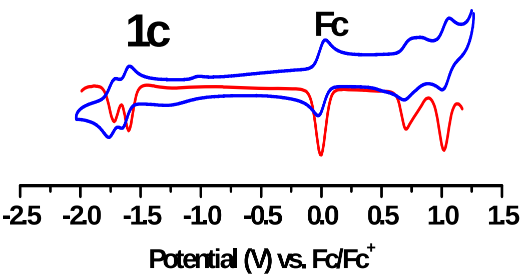
1c
Fc
-2.5 -2.0 -1.5 -1.0 -0.5 0.0 0.5 1.0 1.5
Potential (V) vs. Fc/Fc+

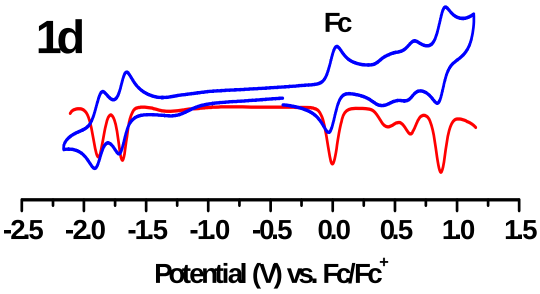
1d
Fc
-2.5 -2.0 -1.5 -1.0 -0.5 0.0 0.5 1.0 1.5
Potential (V) vs. Fc/Fc+

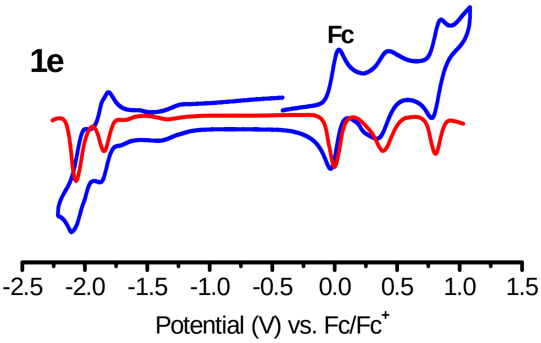
1e
Fc
-2.5 -2.0 -1.5 -1.0 -0.5 0.0 0.5 1.0 1.5
Potential (V) vs. Fc/Fc+

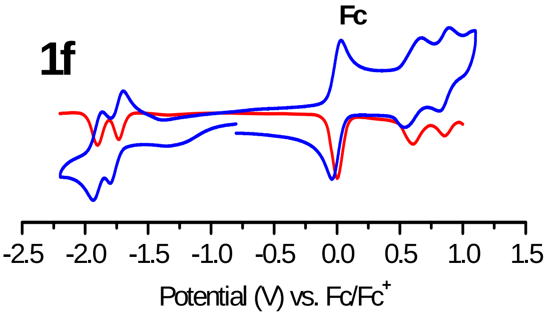
1f
Fc
-2.5 -2.0 -1.5 -1.0 -0.5 0.0 0.5 1.0 1.5
Potential (V) vs. Fc/Fc+

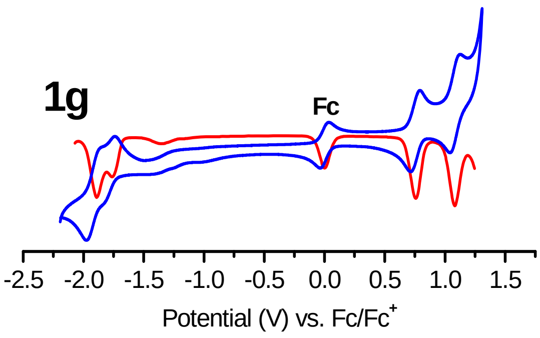
1g
Fc
-2.5 -2.0 -1.5 -1.0 -0.5 0.0 0.5 1.0 1.5
Potential (V) vs. Fc/Fc+

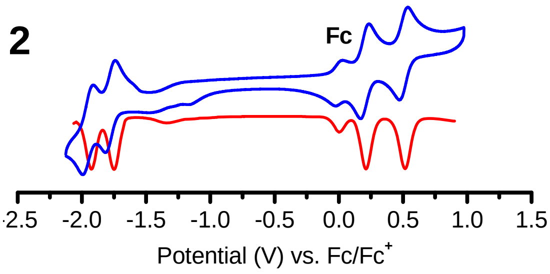
2
Fc
-2.5 -2.0 -1.5 -1.0 -0.5 0.0 0.5 1.0 1.5
Potential (V) vs. Fc/Fc+

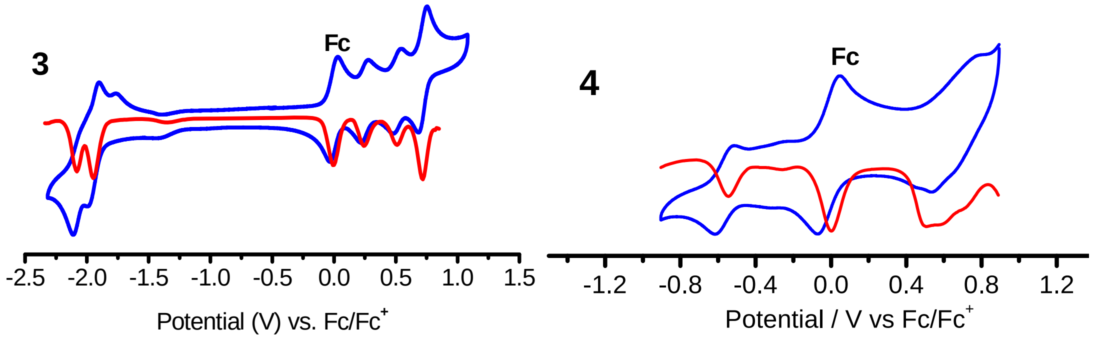

Supplementary Figure 75 | Cyclic voltammograms. **1a**, **1b**, **1c**, **1d**, **1e**, **1f**, **1g**, **2**,**3**in CH_2Cl_2 and **4** in 1,2-dichlorobenzene.

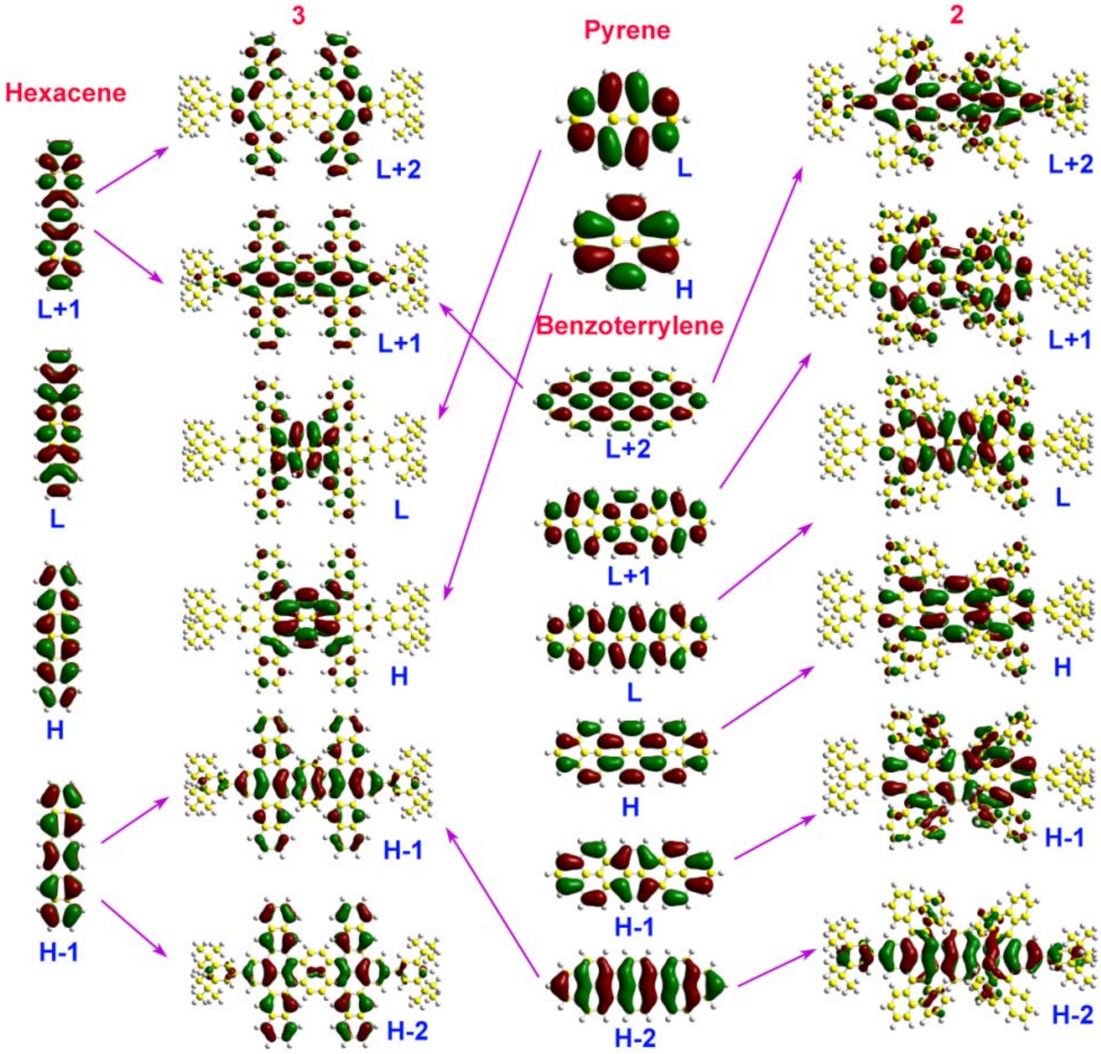

Supplementary Figure 76 | Orbital analysis. Frontier orbitals of **2** and **3**calculated at the B3LYP/6-31G(d) level.

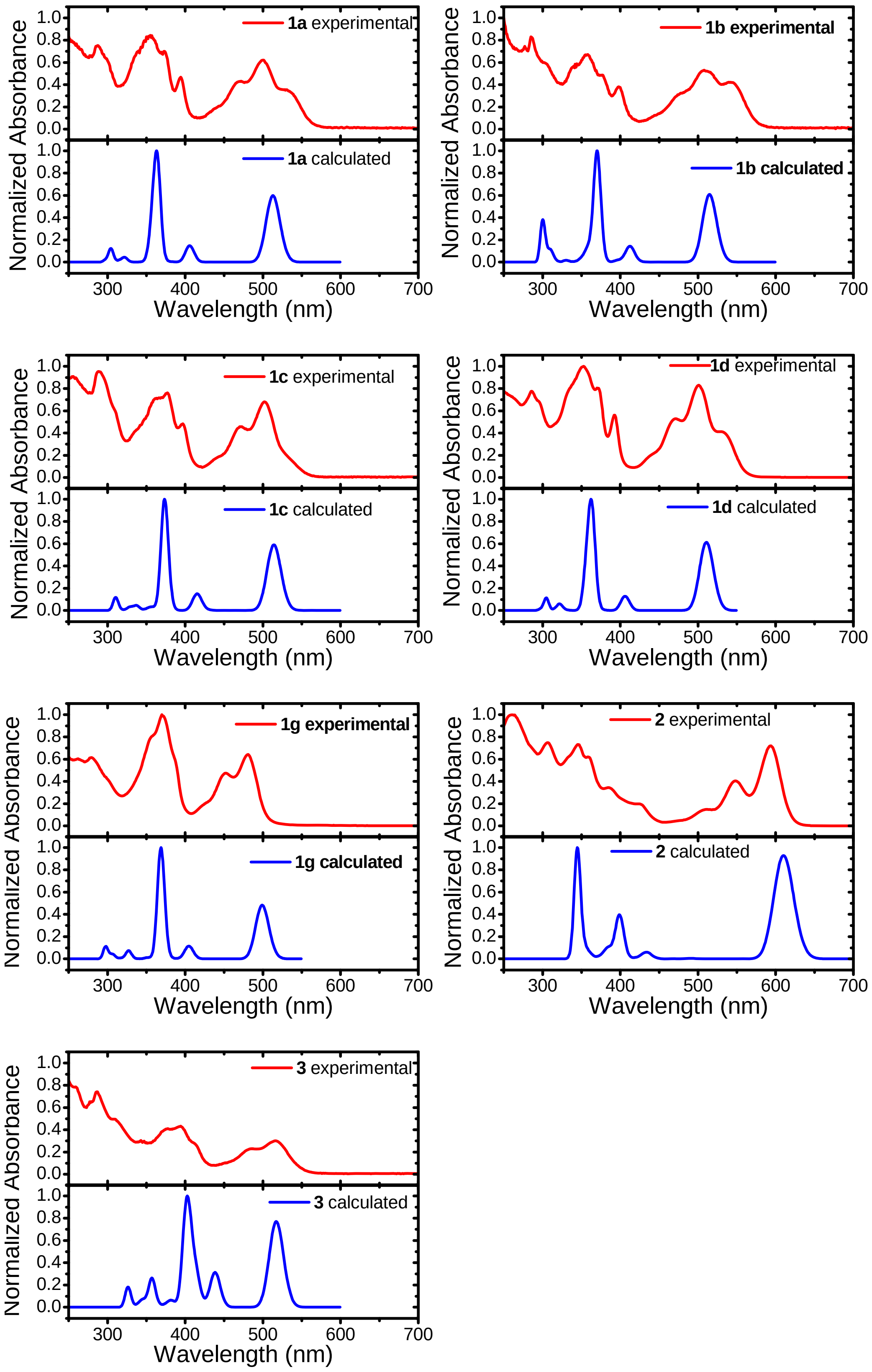

Supplementary Figure 77 | Comparison of the experimental and calculated UV-vis absorption spectra.

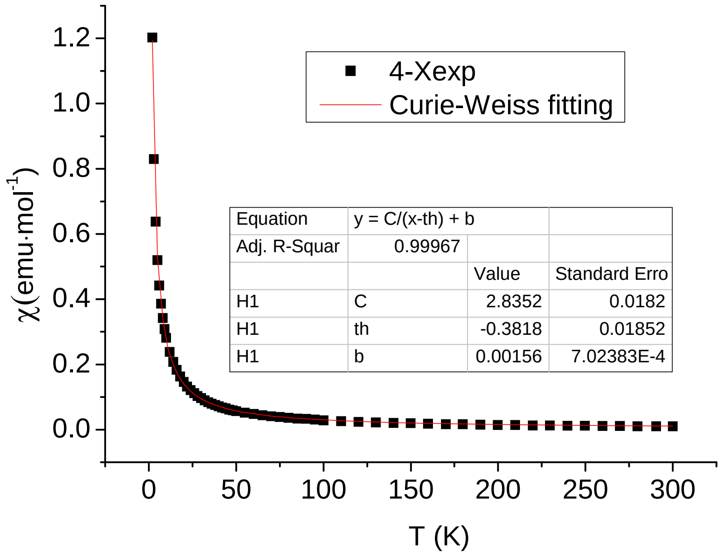

Supplementary Figure 78 | χ vs T plotof 4 and fitting with Curie-Weiss Law. C = 2.84 emu· mol^{-1}; θ = -0.38 K, which indicated small intermolecular antiferromagnetic interactions. Temperature independent contribution = 0.00156 emu· mol^{-1}. According to Curie-Weiss Law:

$\chi_p = \frac{C}{T-\theta}$, $\chi_{exp} = \frac{C}{T-\theta} + \chi T$ ($\chi T = \chi dia + \chi_{Pauli}$ = temperature independent contribution).

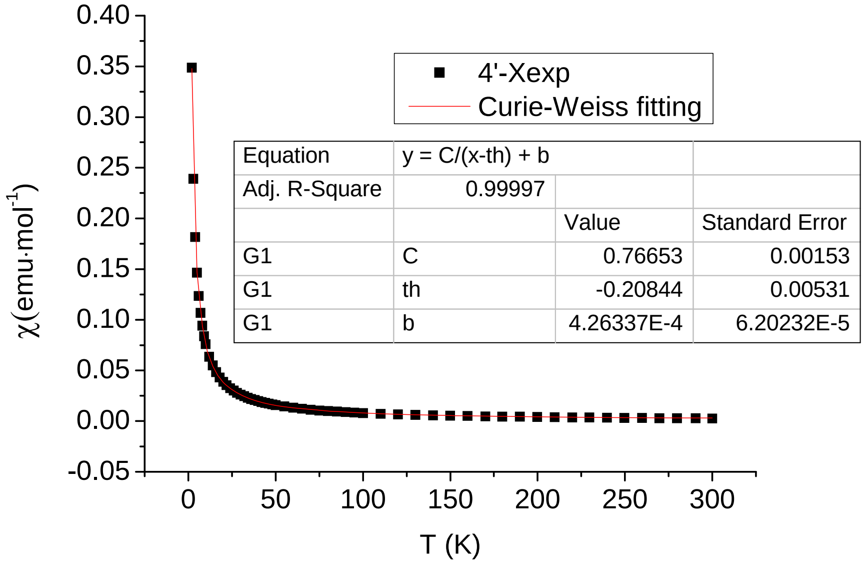

Supplementary Figure 79 | χ vs T plotof 4' and fitting with Curie-Weiss Law. C = 0.77 emu· mol^{-1}; θ = -0.21 K, which indicated small intermolecular antiferromagnetic interactions.Temperature independent contribution = 0.00043 emu· mol^{-1}. According to Curie-Weiss Law: $\chi_p = \frac{C}{T-\theta}$, $\chi_{exp} = \frac{C}{T-\theta} + \chi T$ ($\chi T = \chi dia + \chi_{Pauli}$ = temperature independent contribution).

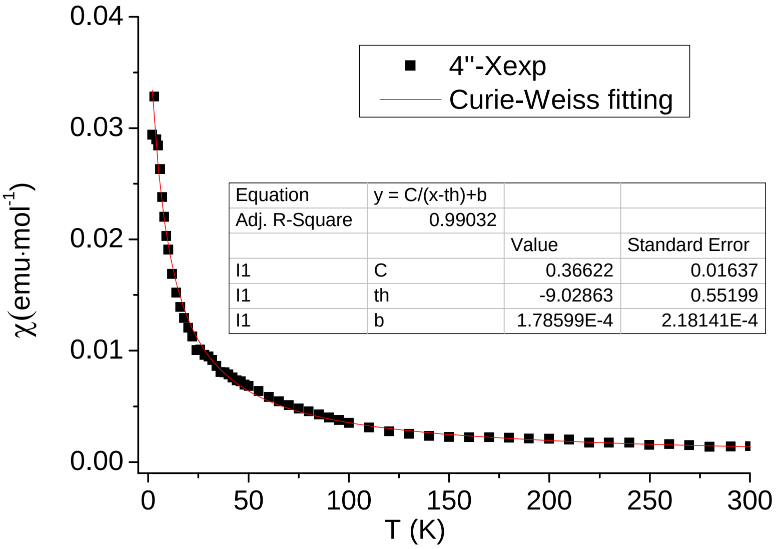

Supplementary Figure 80 | χ vs T plotof 4'' and fitting with Curie-Weiss Law. C = 0.37 emu· mol^{-1}; θ = -9.03 K, which indicated bigger intermolecular antiferromagnetic interactions than **4** and **4'**.Temperature independent contribution = 0.00018 emu· mol^{-1}. According to Curie-Weiss Law: $\chi_p = \frac{C}{T-\theta}$, $\chi_{exp} = \frac{C}{T-\theta} + \chi T$ ($\chi T = \chi_{dia} + \chi_{Pauli}$ = temperature independent contribution).

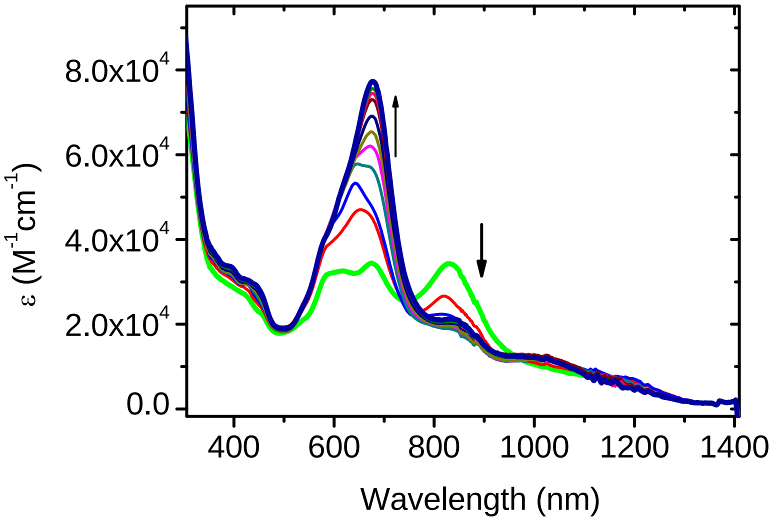

Supplementary Figure 81 | UV-Vis-NIR spectra of 4''in the presence of Zinc dust. Solvent: CH_2Cl_2.

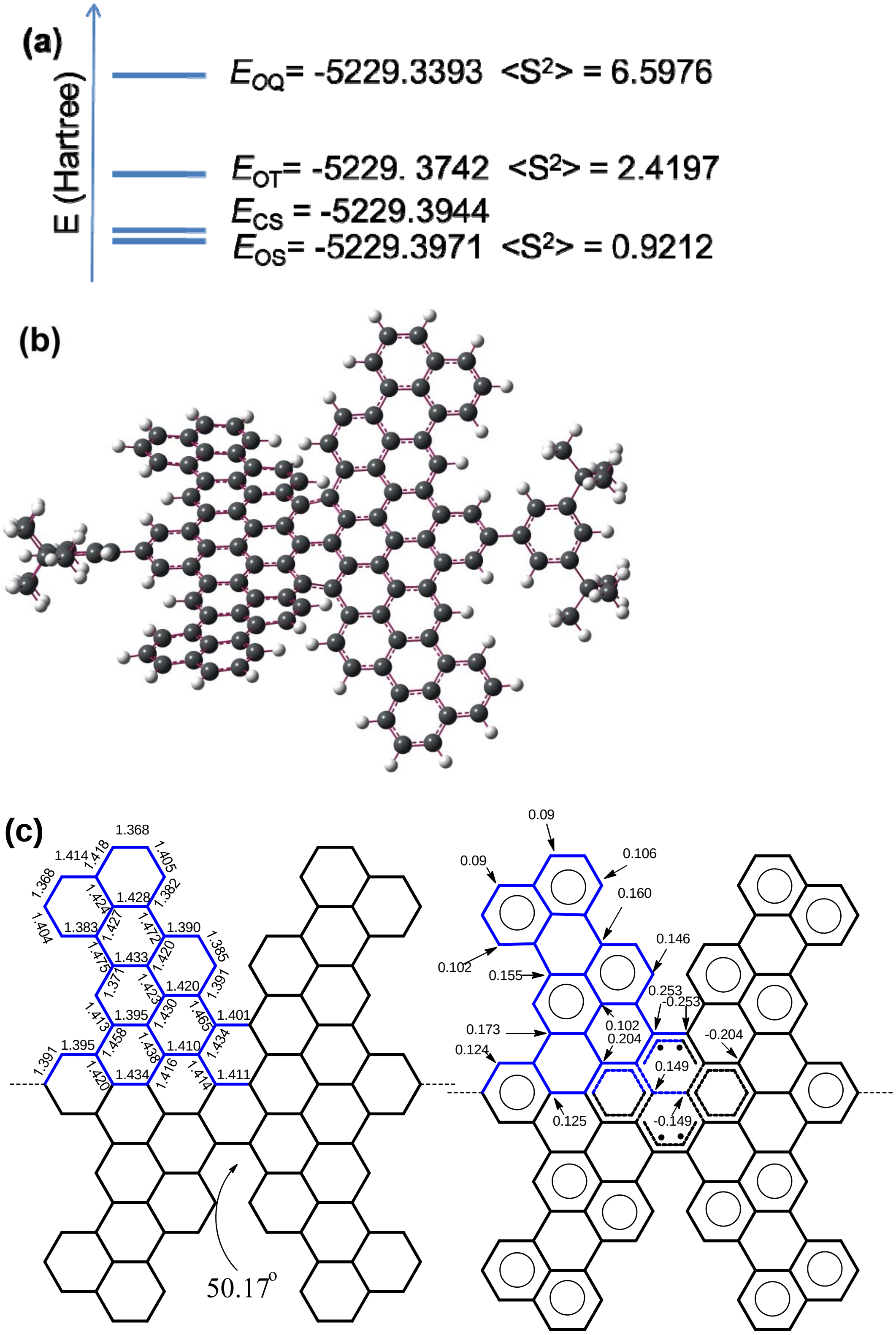

(a)
E (Hartree)
E_{OQ}= -5229.3393 <S²> = 6.5976
E_{OT}= -5229. 3742 <S²> = 2.4197
E_{CS} = -5229.3944
E_{OS}= -5229.3971 <S²> = 0.9212
(b)
(c)
50.17°

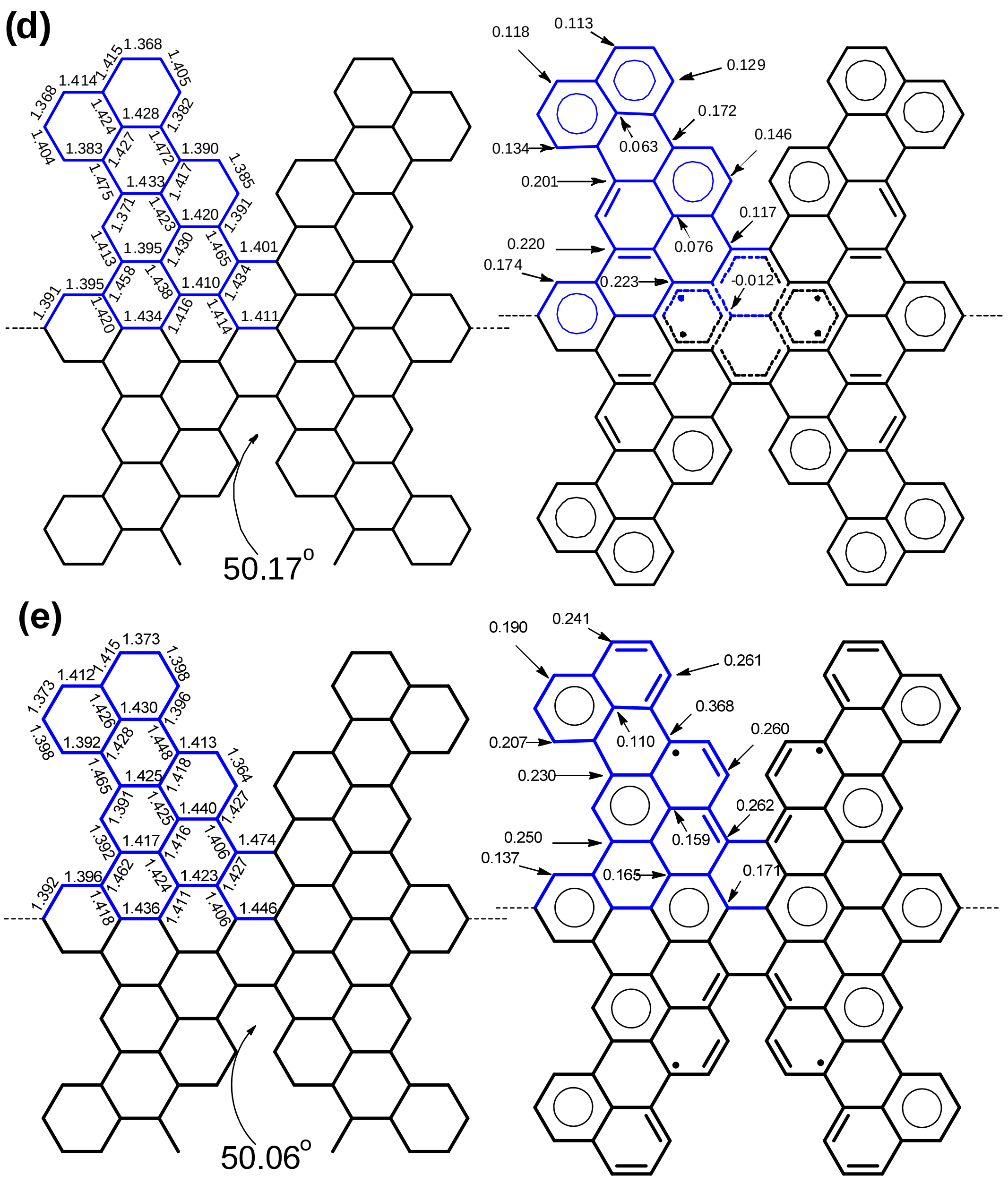

Supplementary Figure 82 | Calculation of 4. (a) Relative stability of the each electronic states (CS: closed shell, ST: singlet tetraradical, and QT: quintet tetraradical) of **4** obtained by (U) CAM-B3LYP/6-31G* level calculations. (b) The UCAM--UB3LYP optimized structure of **4**. The selected bond length (Å), the dihedral angles between the neighboring pentacene-annelated-tribenzo-octacene cores, and the spin density are shown for singlet tetraradical (c),triplet tetraradical (d) and quintet tetraradical (e).

Supplementary Table 1| Relative Energies (kJ/mol) of Conformers of 1–3 Calculated at the B3LYP/6-31G(d) Level of DFT [a]

Compd	*t*TT-	sTT-	sAA-	*t*AA-	AT-
**1a**	9.88	3.36	0.00	20.29	7.58
**1b**	10.72	8.45	0.00	21.22	6.77
**1c**	5.40	7.44	0.00	19.32	5.58
**1d**	10.23	3.03	0.00	20.51	7.50
**1g**	23.76	24.65	0.00	13.61	14.39
**2**	6.91	0.00	19.46	11.00	6.95
**3**	16.03	9.34	0.00	18.97	10.08

[a]Energy of sAA-**1**, sAA-**3**, and sTT-**2** was set as 0.

Supplementary Table 2 │ Crystal data and structure refinement for 1e.

Identification code	**1e**
Empirical formula	4(C80H62F4), 6(CHCl3), 3(C3H6O)
Formula weight	5287.61
Temperature	100(2) K
Wavelength	1.54184 Å
Crystal system, space group	triclinic, P-1
Unit cell dimensions	a = 14.9996(2 Å α = 76.5448(11)°.<br>b =23.1568(3) Å β = 84.5867(11)°.<br>c = 44.2689(6) Å 76.0922(12)°.
Volume	14504.0(4) Å ^{3}
Z, Calculated density	2, 1.211 mg/m^3
Absorption coefficient	2.089 mm^{-1}
F(000)	5512
Crystal size	0.3× 0.1×0.08 mm
Theta range for data collection	3.7040 to 74.8890°.
Limiting indices	-12<=h<=18, -26<=k<=27, -52<=l<=53
Reflections collected / unique	97025 / 43256 [R(int) = 0.1334]
Completeness to theta = 68.251	99.8 %
Absorption correction	multi-scan
Max. and min. transmission	1.000 and 0.538
Refinement method	Full-matrix least-squares on F^2
Data / restraints / parameters	52971 / 198 / 3550
Goodness-of-fit on F^2	2.396
Final R indices [I>2sigma(I)]	R1 = 0.1334, wR2 = 0.3556
R indices (all data)	R1 = 0.1476, wR2 = 0.3661
Largest diff. peak and hole	1.478 and -2.226 e.A^{-3}

Supplementary Table 3 | Optical and Electrochemical Properties of 1–3.

Compd	λ_{abs} (nm) [ε, ($M^{-1}\cdot cm^{-1}$)]	λ_{em} (nm)(Φ_f)	Solid state λ_{abs} (nm)	Solid state λ_{em} (nm)	$E_{1/2}^{ox}$ (V)	$E_{1/2}^{red}$ (V)	HOMO/LUMO (eV)	E_g^E (eV)	E_g (eV)^{opt}
**1a**	499,530 (36,800)	524 (0.83)	502,547	599,666	0.35, 0.68	–1.86, –2.10	–5.15, –2.94	2.21	2.17
**1b**	507,542 (52,600)	532 (0.80)	508,588	650	0.28, 0.58	–1.83, –2.07	–5.08, –2.97	2.11	2.12
**1c**	502 (66,600)	526 (0.73)	474	716	0.70, 1.02	–1.60, –1.72	–5.50, –3.20	2.30	2.24
**1d**	500,530 (37,800)	521 (0.89)	480,514	629	0.42, 0.63, 0.87	–1.70, –1.88	–5.22, –3.10	2.12	2.18
**1e**	494,526 (50,100)	516 (0.87)	477,510	628	0.39, 0.81	–1.85, –2.07	–5.19, –2.95	2.24	2.22
**1f**	497,530 (61,800)	519 (0.85)	477,513	630	0.60, 0.86	–1.74, –1.90	–5.40, –3.06	2.34	2.20
**1g**	480 (60,100)	512 (0.81)	460,491	623	0.85, 1.08	–1.76, –1.89	–5.65, –3.04	2.61	2.43
**2**	593 (95,700)	617 (0.52)	605	641	0.21, 0.51	–1.75, –1.93	–5.01, –3.05	1.96	1.97
**3**	515 (78,300)	545 (0.89)	529	626	0.25, 0.51, 0.72	–1.95, –2.08	–5.05, –2.85	2.20	2.22

[a]$E_{1/2}^{ox}$, $E_{1/2}^{red}$: Half-wave potentials of the oxidative and reductive waves, respectively, measured in CH_2Cl_2; scan rate: 100 mV/s; potentials vs Fc/Fc^+ couple. E_g^E: Electrochemical HOMO–LUMO gap. [b]For an irreversible wave, the potential was determined by DPV. [c]Determined from λ_{onset} in CH_2Cl_2.

Supplementary Table 4 | Calculated HOMOs and LUMOs orbitals and energies (eV). Compounds *s*AA-**1**, *t*AA-**1**, AT-**1**, *s*TT-**1**, *t*TT-**1,** *s*AA-**1b,** *s*AA-**1c**,*s*AA-**d**,*s*AA-**1g,** *s*AA-**2** and *s*AA-**3.**

	LUMO+2	LUMO+1	LUMO	HOMO	HOMO-1	HOMO-2
*t*TT-1a	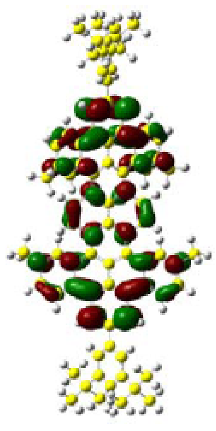 -1.10	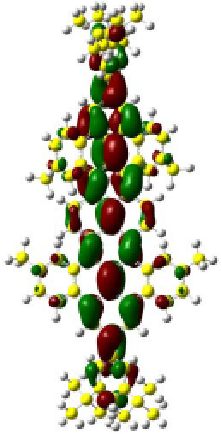 -1.27	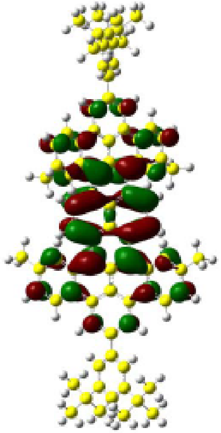 -2.19	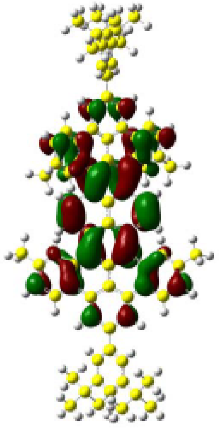 -4.56	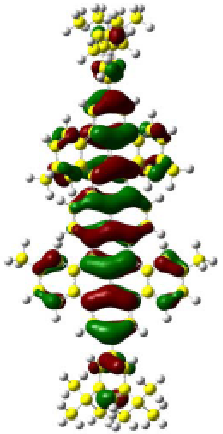 -5.30	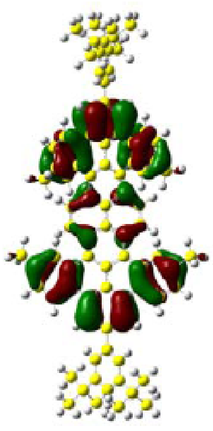 -5.56
*s*TT-1a	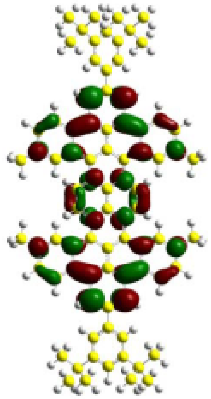 -1.10	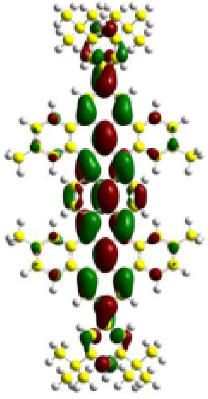 -1.32	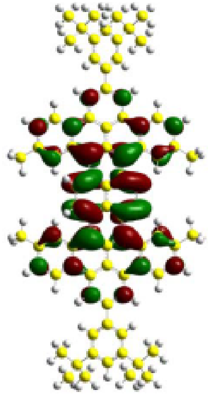 -2.17	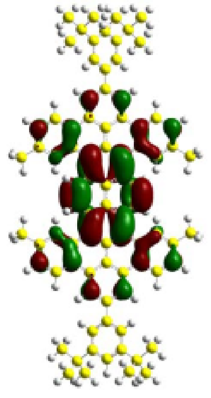 -4.61	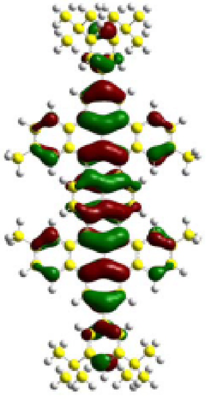 -5.28	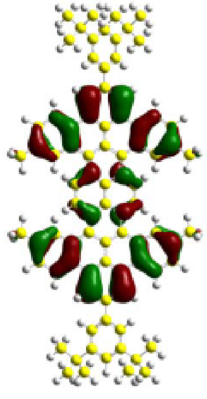 -5.51

*s*AA-1a	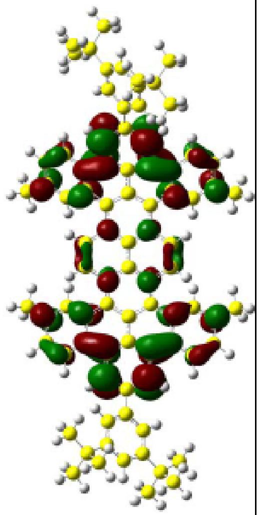	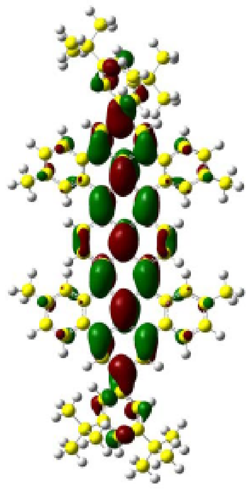	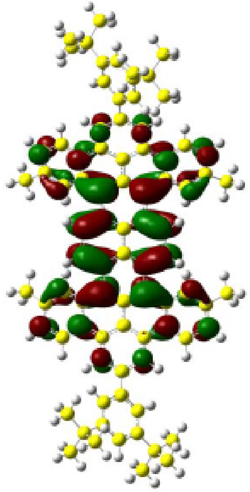	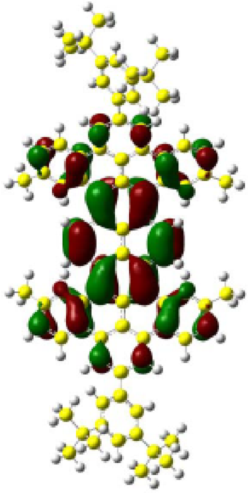	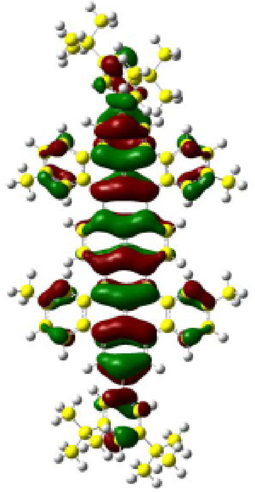	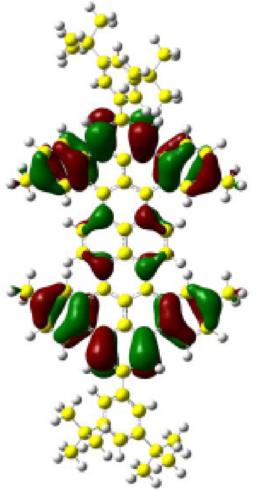
	-1.05	-1.42	-2.00	-4.63	-5.25	-5.56
AT-1a	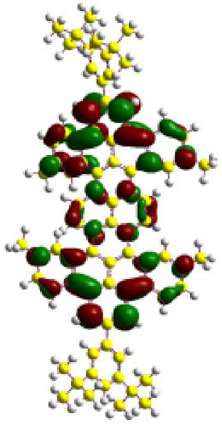	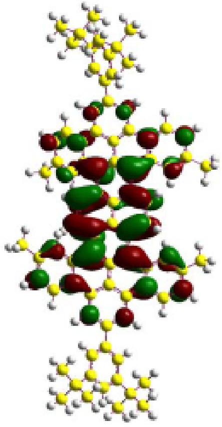	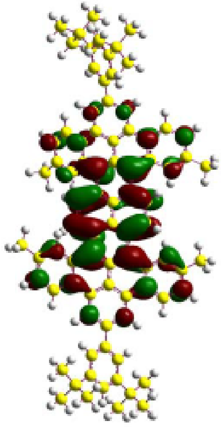	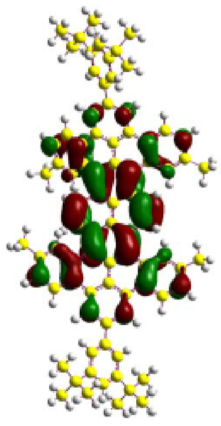	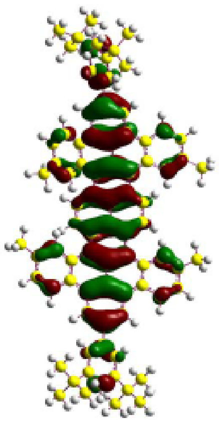	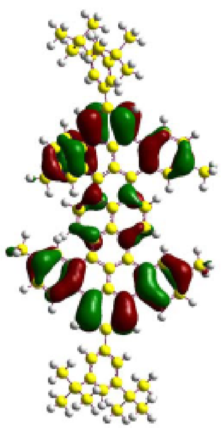
	-1.07	-1.36	-2.10	-4.62	-5.28	-5.57

tAA-1a	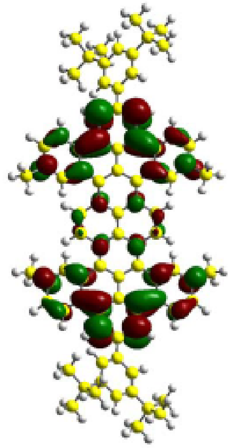 -1.03	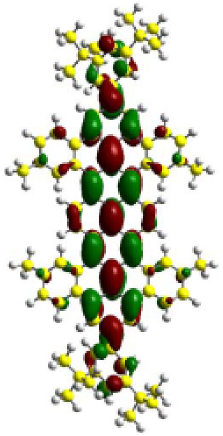 -1.43	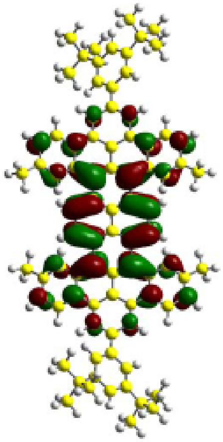 -2.00	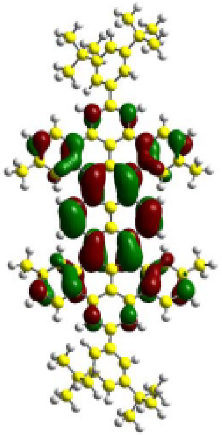 -4.65	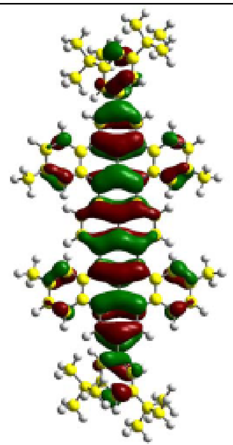 -5.25	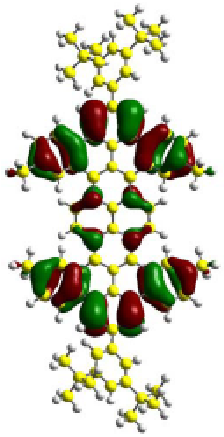 -5.59
*s*AA-1b	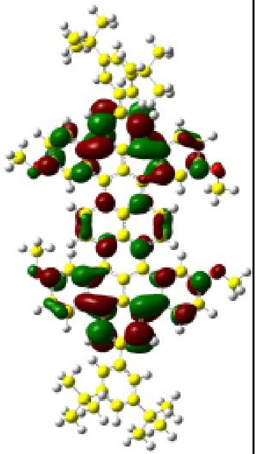 -0.91	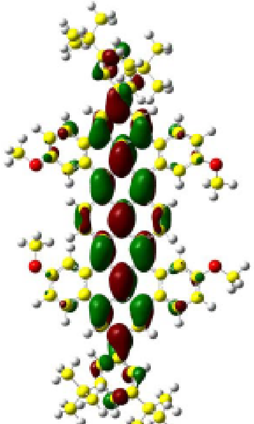 -1.39	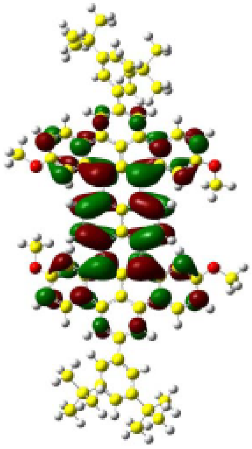 -1.99	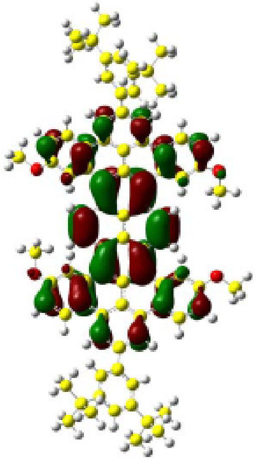 -4.63	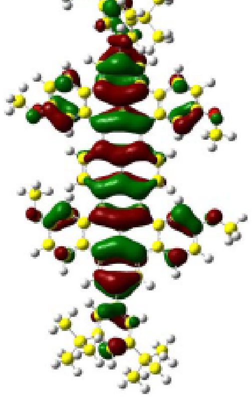 -5.16	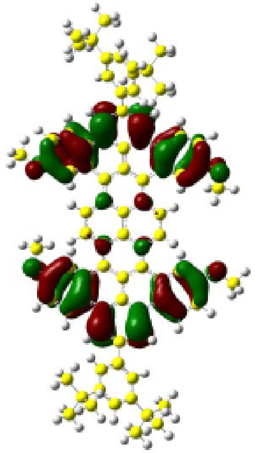 -5.30

*s*AA-1c	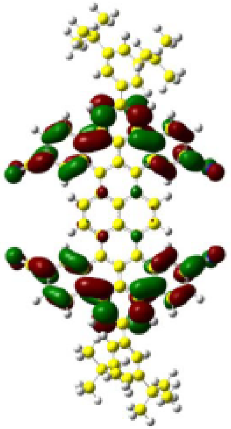<br>-2.13	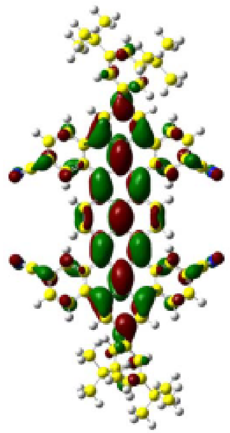<br>-2.22	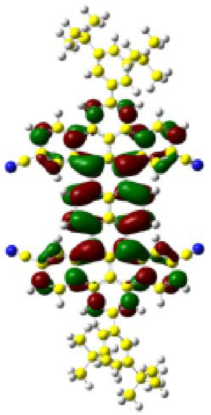<br>-2.78	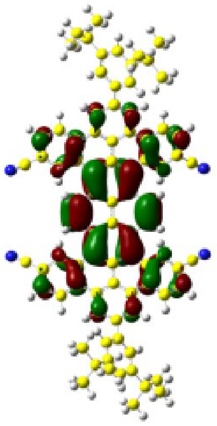<br>-5.42	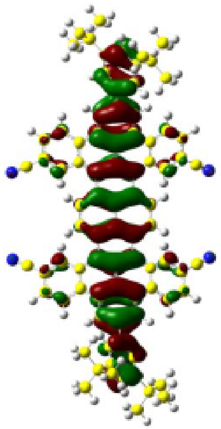<br>-5.95	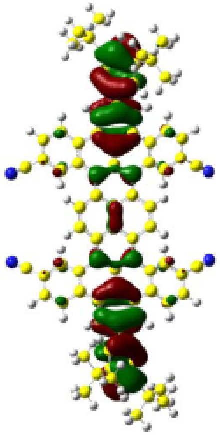<br>-6.31
*s*AA-1d	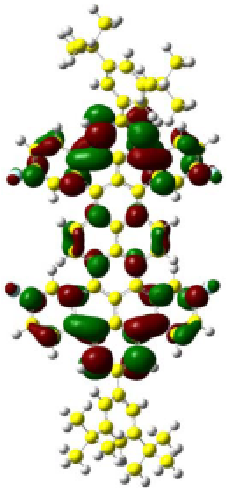<br>-1.24	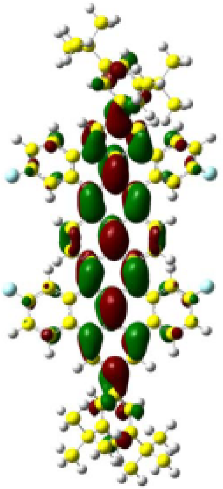<br>-1.67	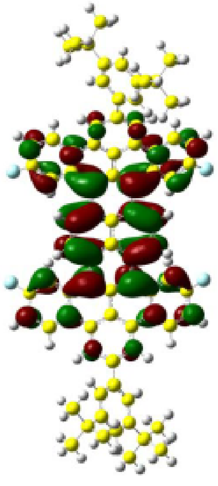<br>-2.31	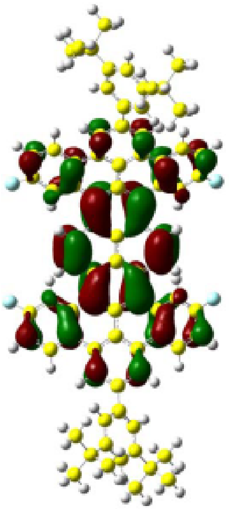<br>-4.95	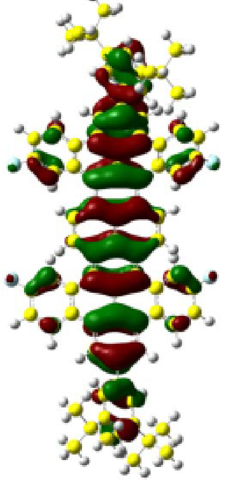<br>-5.50	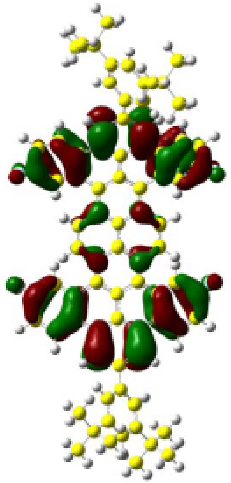<br>-5.80

*s*AA-1g	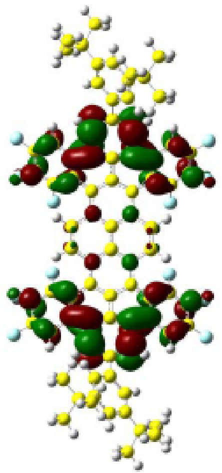 -1.63	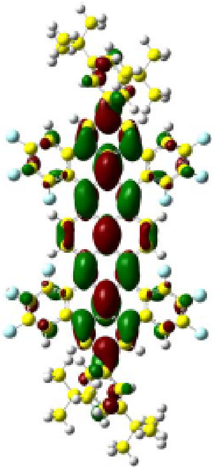 -1.94	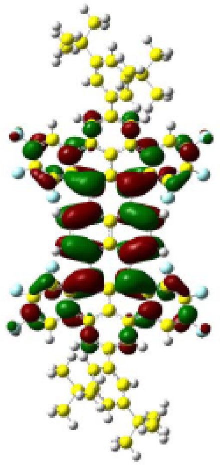 -2.45	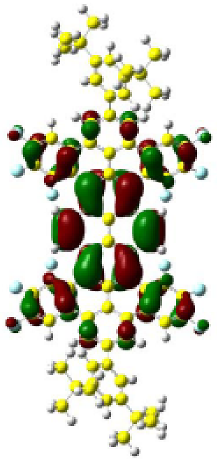 -5.16	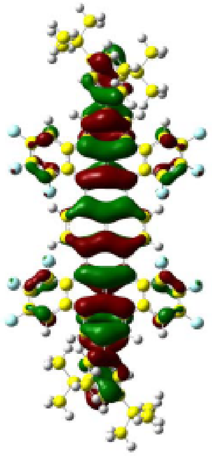 -5.70	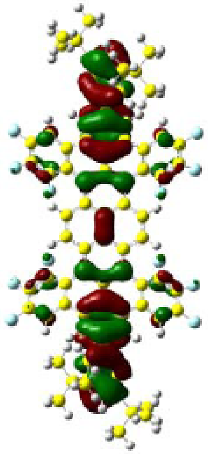 -6.12
*s*AA-2	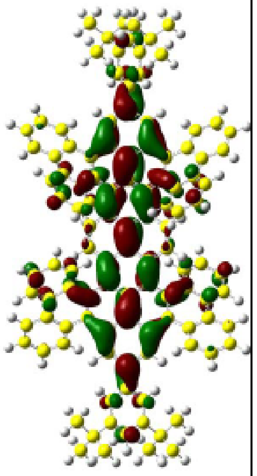 -1.46	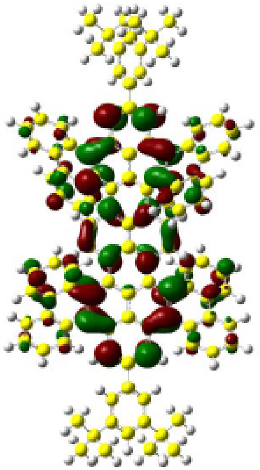 -1.59	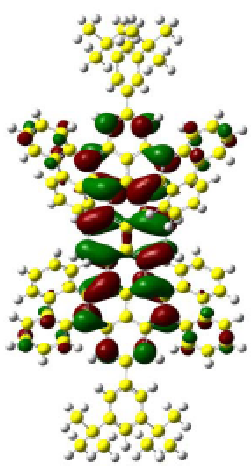 -2.27	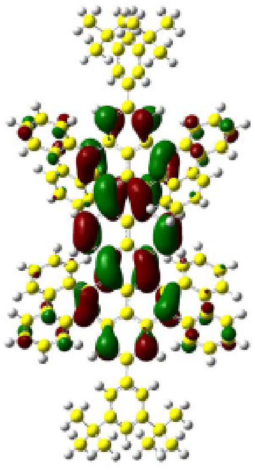 -4.47	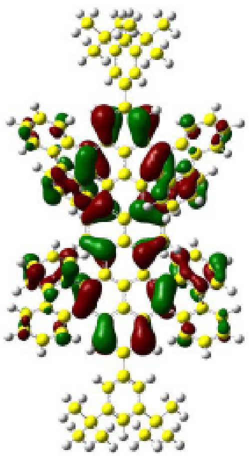 -5.16	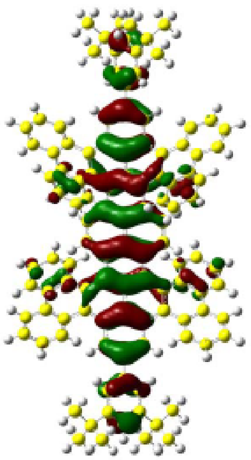 -5.38

*s*AA-3	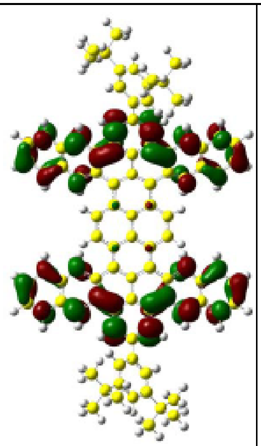 -1.47	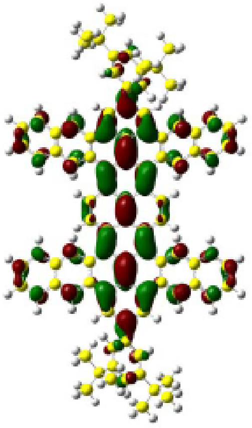 -1.70	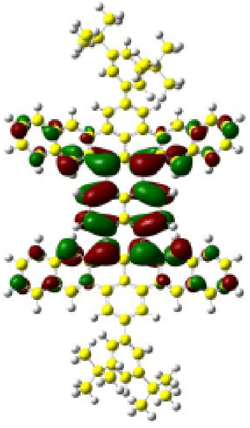 -2.10	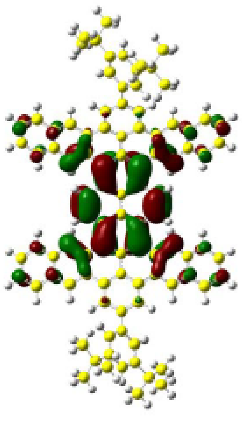 -4.75	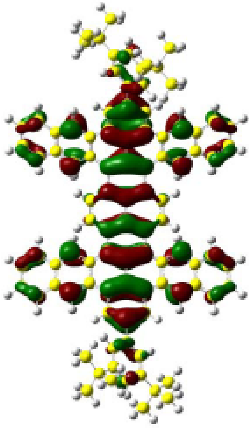 -5.12	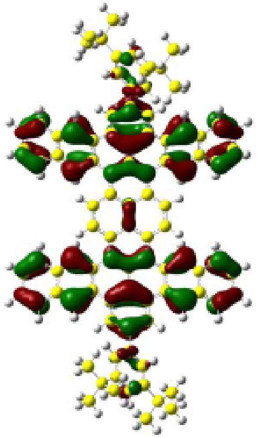 -5.44

Supplementary Table 5 | Absorption wavelengths and oscillator strength. *s*AA-**1a**, evaluated by the TD-DFT (B3LYP/6-31G(d)) calculation.

Excitation energies (eV)	Absorption[nm](oscillator strength)	Assignments (%)	
2.42	512.8 （1.00）	289H ->L290	81
3.06	405.7（0.25）	H-1->L	50
3.41	363.7（1.53）	H-4->L	16
		H-1->L+1	68
		H ->L+3	6
3.47	357.1（0.42）	H-4->L	45
		H->L+3	25
3.85	322.1（0.06）	H-7->L	42
		H-2->L+3	3
		H->L+5	3
3.93	315.5（0.03）	H-5->L	62
		H-4->L+1	3
4.07	304.4（0.20）	H-1->L+3	34
		H->L+7	17
4.17	297.5（0.03）	H-7->L	22
		H-3->L+1	7
		H-1->L+4	40
		H->L+5	7

HF=-3245.7532641

Supplementary Table 6│Absorption wavelengths and oscillator strength. *s*AA-**1b**, evaluated by the TD-DFT (B3LYP/6-31G(d)) calculation.

Excitation energies (eV)	Absorption[nm](oscillator strength)	Assignments (%)	
2.41	514.7(0.93)	H305 ->L306	82
3.01	412.4(0.22)	H ->L-1	45
3.12	397.5(0.03)	H-3 ->L	89
		H ->L+4	7
3.35	370.1(1.53)	H-1 ->L+1	81
3.45	358.9(0.18)	H-1 ->L+1	7
		H ->L+4	80
3.53	351.4(0.05)	H-5 ->L	88
		H-1 ->L+4	2
3.75	330.3(0.03)	H-4 ->L+1	62
3.91	317.0(0.01)	H-3 ->L+1	4
		H-2 ->L+3	3
		H-1 ->L+4	34
		H ->L+7	42
		H ->L+8	7
3.94	314.3(0.01)	H-1 ->L+3	52
4.00	310.1(0.16)	H ->L+7	15
4.09	303.4(0.11)	H-2 ->L+2	22
4.14	299.8(0.53)	H-5 ->L+1	67
		H-2 ->L+2	66
		H-1 ->L+7	18

HF=-3546.5708605

Supplementary Table 7 | Absorption wavelengths and oscillator strength. *s*AA-**1c**, evaluated by the TD-DFT (B3LYP/6-31G(d)) calculation.

Excitation energies (eV)	Absorption[nm](oscillator strength)	Assignments (%)	
2.41	514.（0.95）	H -> L	82
2.98	415.5（0.23）	H-1-> L	47
		H-> +1	38
3.01	411.5（0.01）	H-> L+3	92
3.23	384.（0.01）	H-3-> L	85
		H-2-> L+1	3
		H-> L+2	3
3.32	373.5（1.61）	H-4-> L	3
		H-1->L+1	86
3.46	358.2（0.03）	H-4-> L	87
		H->L+3	3
3.50	354.7（0.02）	H-4->L+1	5
		H-2->L+2	11
		H-1->L+3	72
3.55	349.7（0.01）	H-9-> L	8
		H-2-> L+1	6
		H-1->L+4	5
		H->L+6	77
3.67	337.4（0.06）	H-7-> L	69
		H-1->L+3	3
		H-> L+5	3
3.68	337.1（0.01）	H-7-> L	15
		H-6-> L	81
3.77	328.9（0.05）	H-2->L+1	70
3.99	310.6（0.17）	H-3-> L+2	79
4.02	308.1（0.02）	H-9-> L	20
		H-3-> L+3	36
		H-2-> L+1	6
		H-1-> L+4	8
		H-1-> L+7	11

HF=-3457.4502895

Supplementary Table 8 | Absorption wavelengths and oscillator strength. *s*AA-**1d**, evaluated by the TD-DFT (B3LYP/6-31G(d)) calculation.

Excitation energies (eV)	Absorption[nm](oscillator strength)	Assignments (%)	
2.43	510.8(1.01)	289H->L	81
3.05	406.1(0.21)	H->L+1	46
3.41	363.4(1.421)	H-4->L	9
		H-1->L+1	63
		H->L+3	17
3.48	356.467(0.58)	H-4->L	17
		H-1->L+1	25
		H -> L+3	49
3.55	349.241(0.01)	H-10->L	11
		H -> L+5	85
3.84	322.9(0.03)	H-9->L	41
3.85	321.9(0.06)	H-3->L+1	52
		H-1 -> L+4	2
3.92	316.6(0.02)	H-1->L+3	8
		H->L+7	75
4.07	304.7(0.18)	H-1->L+3	31
4.16	298.1(0.03)	H-10->L	29
		H-3->L+1	6
		H-1->L+4	35

HF=-3485.4126198

Supplementary Table 9 | Absorption wavelengths and oscillator strength. *s*AA-**1g**, evaluated by the TD-DFT (B3LYP/6-31G(d)) calculation.

Excitation energies (Ev)	Absorption[nm](oscillator strength)	Assignments (%)	
2.48	499.0(0.93)	H321 ->L322	81
3.06	404.7(0.22)	H-1 ->L	51
3.23	383.8(0.01)	H ->L+3	88
3.36	368.8(1.93)	H-1 ->L+1	86
3.53	351.7(0.02)	H-4 ->L	84
		H ->L+3	7
3.79	327.6(0.11)	H-8 ->L	22
		H-2 ->L+1	48
		H-1 ->L+4	4
		H ->L+5	13
3.81	325.5(0.03)	H-5 ->L	81
		H ->L+7	6
3.91	316.9(0.01)	H-4 ->L+1	22
		H-3 ->L+4	3
		H ->L+7	61
4.04	306.7(0.08)	H-4 ->L+1	23
		H-2 ->L+2	23
4.14	299.6(0.07)	H-2 ->L+1	6
		H-1 ->L+4	36
4.17	297.6(0.01)	H-11 ->L	5
		H-2 ->L+2	5
		H ->L+8	59
		H ->L+10	8
4.18	296.9(0.15)	H-13 ->L	2
		H-11 ->L	2
		H-3 ->L+4	6
		H-2 ->L+2	47
		H ->L+7	3

HF=-4279.2018895

Supplementary Table 10 | Absorption wavelengths and oscillator strength. *s*AA-**2**, evaluated by the TD-DFT (B3LYP/6-31G(d)) calculation.

Excitation energies (eV)	Absorption[nm](oscillator strength)	Assignments (%)	
2.03	610.0 (1.12)	H->L	82
2.86	433.6 (0.07)	H-2 ->L	46
		H ->L+2	26
3.11	398.9 (0.47)	H-2 ->L	10
		H ->L+2	4
		H ->L+4	42
3.23	384.2 (0.09)	H-6 ->L	64
		H-1 ->L+1	12
3.44	360.1(0.04)	H-5 ->L	3
		H-2 ->L+1	34
		H-1 ->L+2	13
3.50	353.9(0.06)	H-10 ->L	74
3.511	353.1 (0.04)	H-8 ->L	9
		H-2 ->L+1	35
		H-1 ->L+2	4
		H ->L+10	41
3.60	344.7 (1.15)	H-2 ->L+2	88

HF=-4316.567482

Supplementary Table 11 | Absorption wavelengths and oscillator strength. *s*AA-**3**, evaluated by the TD-DFT (B3LYP/6-31G(d)) calculation.

Excitation energies (eV)	Absorption[nm](oscillator strength)	Assignments (%)	
2.40	517.2(0.84)	H325 ->L326	82
2.83	438.5(0.33)	H-1 ->L	44
3.00	413.5(0.31)	H-4 ->L	57
		H-1 ->+1	38
3.03	409.1(0.01)	H-3 ->L	87
3.08	402.2(1.03)	H-1 ->L+1	49
3.23	383.4(0.04)	H-4 ->L+1	20
		H-1 ->L+3	65
3.27	379.7(0.03)	H-2 ->L+1	30
		H ->L+5	35
3.32	373.4(0.01)	H-5 ->L	48
		H-1 ->L+4	4
		H ->L+5	31
3.39	365.7(0.01)	H-6 ->L	13
		H ->L+6	79
3.47	357.1(0.28)	H-4 ->L+1	63
		H ->L+9	2
3.58	346.0(0.07)	H-6 ->L	80
3.65	339.8(0.02)	H-4 ->L+1	3
		H-2 ->L+2	83
		H-1 ->L+3	6
3.79	327.6(0.07)	H-5->L	7
		H-4 ->L+2	68
3.81	325.8(0.10)	H-1 ->L+6	62
		H ->L+9	3
3.81	325.7(0.04)	H-4 ->L+2	3
		H-3 ->L+4	45
		H-1 ->L+6	11

HF=-3703.0488548

Supplementary Table 12 | Mulliken atomic spin densities of open shell singlet 4.

1	55 C -0.107994	110 C 0.000090	165 H -0.004655
1 C -0.203834	56 C -0.143240	111 H -0.000007	166 C 0.116313
2 C 0.176029	57 C -0.014542	112 H 0.000002	167 C 0.055558
3 C 0.176030	58 C 0.009884	113 H 0.000032	168 H 0.004635
4 C 0.160592	59 C 0.010089	114 C -0.000374	169 C -0.105886
5 C -0.203833	60 C 0.010089	115 C 0.000090	170 H -0.004635
6 C -0.148585	61 C -0.006083	116 H 0.000032	171 C 0.105886
7 C 0.148585	62 C -0.006083	117 H -0.000007	172 C -0.055558
8 C -0.160592	63 H -0.000513	118 H 0.000002	173 C -0.055564
9 C -0.176029	64 H -0.000513	119 C 0.000098	174 H -0.004636
10 C -0.176029	65 H -0.000487	120 H 0.000013	175 C 0.105899
11 C 0.203834	66 C 0.014542	121 H 0.000001	176 H 0.004636
12 C 0.203833	67 C -0.009884	122 H -0.000009	177 C -0.105899
13 C -0.253134	68 C -0.010089	123 C 0.000006	178 C 0.055564
14 C 0.253125	69 C -0.010089	124 H -0.000001	179 H -0.002491
15 C -0.253125	70 C 0.006083	125 H 0.000002	180 C 0.064007
16 C 0.253134	71 C 0.006083	126 H 0.000003	181 H 0.004144
17 C 0.089803	72 H 0.000513	127 H -0.005640	182 C -0.092625
18 C -0.083487	73 H 0.000513	128 H -0.005640	183 H 0.002491
19 C -0.105038	74 H 0.000487	129 H 0.005640	184 C -0.064007
20 C -0.105040	75 C 0.000374	130 H 0.005640	185 H -0.004144
21 C 0.123582	76 C 0.000374	131 C -0.088761	186 C 0.092625
22 C 0.123582	77 C -0.000098	132 C 0.159815	187 H -0.004144
23 H -0.005251	78 H -0.000013	133 C -0.091557	188 C 0.092636
24 H -0.005250	79 H -0.000001	134 C 0.054099	189 H 0.002492
25 C -0.089803	80 H 0.000009	135 C -0.159815	190 C -0.064014
26 C 0.083487	81 C -0.000006	136 C 0.088761	191 H 0.004144
27 C 0.105038	82 H 0.000001	137 C -0.054099	192 C -0.092636
28 C 0.105040	83 H -0.000002	138 C 0.091557	193 H -0.002492
29 C -0.123582	84 H -0.000003	139 C 0.088752	194 C 0.064014
30 C -0.123582	85 C -0.000090	140 C -0.159794	195 H 0.004560
31 H 0.005251	86 H -0.000032	141 C 0.091547	196 C -0.101834
32 H 0.005250	87 H 0.000007	142 C -0.054093	197 H -0.002427
33 C -0.173172	88 H -0.000002	143 C 0.159794	198 C 0.062017
34 C 0.107994	89 C -0.000090	144 C -0.088753	199 H 0.003994
35 C 0.152821	90 H 0.000007	145 C 0.054093	200 C -0.089393
36 C 0.143240	91 H -0.000002	146 C -0.091547	201 H -0.004560
37 C -0.155321	92 H -0.000032	147 C 0.153766	202 C 0.101834
38 C -0.101918	93 C -0.000098	148 C -0.153766	203 H 0.002427
39 C 0.152831	94 H 0.000009	149 C -0.153786	204 C -0.062017
40 C -0.155334	95 H -0.000013	150 C 0.153786	205 H -0.003994
41 C -0.173183	96 H -0.000001	151 H 0.006096	206 C 0.089393
42 C -0.101929	97 C -0.000006	152 C -0.145611	207 H -0.004561
43 C 0.108006	98 H -0.000003	153 H -0.004654	208 C 0.101844
44 C 0.143254	99 H 0.000001	154 C 0.116303	209 H 0.002427

45 C -0.152831	100 H -0.000002	155 H 0.004654	210 C -0.062023
46 C 0.155334	101 C -0.000374	156 C -0.116303	211 H -0.003995
47 C 0.173183	102 C 0.000098	157 H -0.006096	212 C 0.089402
48 C 0.101929	103 H 0.000001	158 C 0.145611	213 H 0.004561
49 C -0.108006	104 H -0.000009	159 H -0.006096	214 C -0.101844
50 C -0.143254	105 H 0.000013	160 C 0.145629	215 H 0.003995
51 C -0.152821	106 C 0.000006	161 H 0.004655	216 C -0.089402
52 C 0.155321	107 H 0.000003	162 C -0.116313	217 H -0.002427
53 C 0.173172	108 H -0.000001	163 H 0.006096	218 C 0.062023
54 C 0.101918	109 H 0.000002	164 C -0.145629	

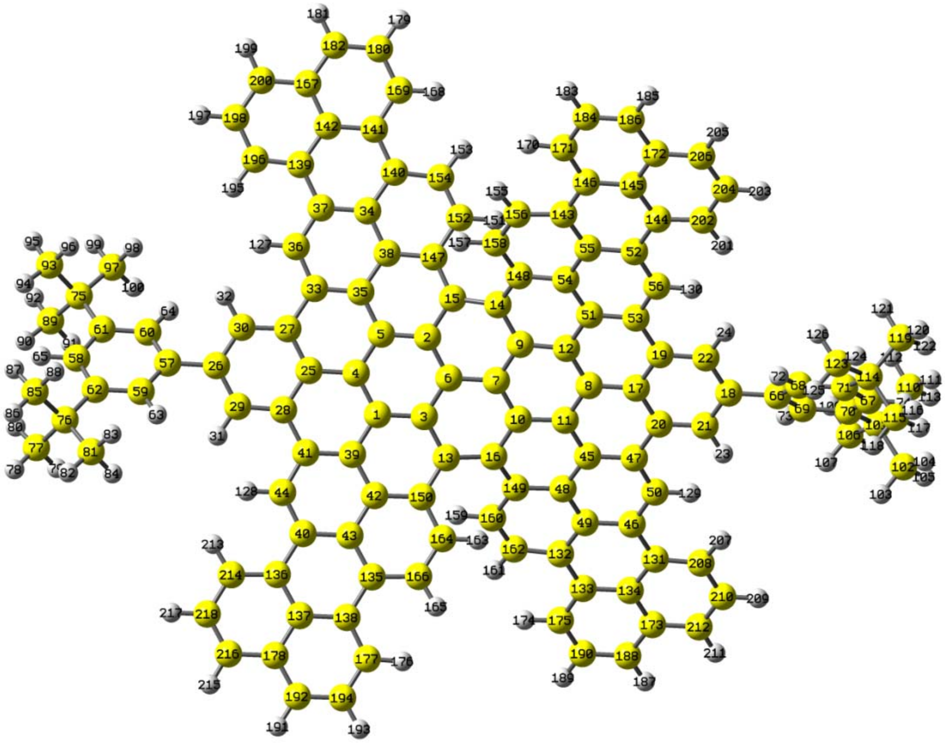

Supplementary Table 13 | Mulliken atomic spin densities of open shell triplet 4.

1	55 C -0.118772	110 C 0.000115	165 H 0.004554
1 C 0.223261	56 C -0.169337	111 H -0.000009	166 C -0.115651
2 C -0.049826	57 C 0.018798	112 H 0.000002	167 C -0.069625
3 C -0.049851	58 C -0.012698	113 H 0.000041	168 H -0.005633
4 C -0.180134	59 C -0.012917	114 C -0.000481	169 C 0.128525
5 C 0.223262	60 C -0.012917	115 C 0.000115	170 H -0.005633
6 C -0.011922	61 C 0.007811	116 H 0.000041	171 C 0.128535
7 C -0.011906	62 C 0.007811	117 H -0.000009	172 C -0.069631
8 C -0.180141	63 H 0.000682	118 H 0.000002	173 C -0.069646
9 C -0.049840	64 H 0.000682	119 C 0.000126	174 H -0.005635
10 C -0.049864	65 H 0.000629	120 H 0.000017	175 C 0.128567
11 C 0.223267	66 C 0.018805	121 H 0.000002	176 H -0.005634
12 C 0.223268	67 C -0.012705	122 H -0.000011	177 C 0.128557
13 C 0.116925	68 C -0.012924	123 C 0.000008	178 C -0.069641
14 C 0.116894	69 C -0.012924	124 H -0.000001	179 H 0.003038
15 C 0.116879	70 C 0.007818	125 H 0.000002	180 C -0.078027
16 C 0.116940	71 C 0.007818	126 H 0.000004	181 H -0.005067
17 C 0.099584	72 H 0.000683	127 H 0.006525	182 C 0.113398
18 C -0.110684	73 H 0.000683	128 H 0.006526	183 H 0.003038
19 C -0.132395	74 H 0.000630	129 H 0.006526	184 C -0.078035
20 C -0.132402	75 C -0.000481	130 H 0.006525	185 H -0.005067
21 C 0.174327	76 C -0.000481	131 C -0.111345	186 C 0.113408
22 C 0.174325	77 C 0.000126	132 C 0.171718	187 H -0.005069
23 H -0.007432	78 H 0.000017	133 C -0.105310	188 C 0.113436
24 H -0.007432	79 H 0.000002	134 C 0.063261	189 H 0.003039
25 C 0.099578	80 H -0.000011	135 C 0.171706	190 C -0.078053
26 C -0.110682	81 C 0.000008	136 C -0.111341	191 H -0.005068
27 C -0.132392	82 H -0.000001	137 C 0.063256	192 C 0.113427
28 C -0.132400	83 H 0.000002	138 C -0.105300	193 H 0.003039
29 C 0.174327	84 H 0.000004	139 C -0.111323	194 C -0.078045
30 C 0.174326	85 C 0.000115	140 C 0.171656	195 H -0.005980
31 H -0.007432	86 H 0.000041	141 C -0.105276	196 C 0.133591
32 H -0.007432	87 H -0.000009	142 C 0.063244	197 H 0.003094
33 C 0.219997	88 H 0.000002	143 C 0.171668	198 C -0.079724
34 C -0.118763	89 C 0.000115	144 C -0.111327	199 H -0.005279
35 C -0.133018	90 H -0.000009	145 C 0.063249	200 C 0.117935
36 C -0.169332	91 H 0.000002	146 C -0.105286	201 H -0.005980
37 C 0.201369	92 H 0.000041	147 C -0.101752	202 C 0.133596
38 C 0.075762	93 C 0.000126	148 C -0.101765	203 H 0.003094
39 C -0.133017	94 H -0.000011	149 C -0.101770	204 C -0.079728
40 C 0.201396	95 H 0.000017	150 C -0.101758	205 H -0.005280
41 C 0.220020	96 H 0.000002	151 H -0.005935	206 C 0.117940
42 C 0.075767	97 C 0.000008	152 C 0.145619	207 H -0.005981
43 C -0.118787	98 H 0.000004	153 H 0.004553	208 C 0.133621
44 C -0.169348	99 H -0.000001	154 C -0.115626	209 H 0.003095

45	C	-0.133024	100	H	0.000002	155	H	0.004554	210	C	-0.079743
46	C	0.201401	101	C	-0.000481	156	C	-0.115637	211	H	-0.005281
47	C	0.220022	102	C	0.000126	157	H	-0.005936	212	C	0.117962
48	C	0.075777	103	H	0.000002	158	C	0.145630	213	H	-0.005981
49	C	-0.118796	104	H	-0.000011	159	H	-0.005937	214	C	0.133617
50	C	-0.169353	105	H	0.000017	160	C	0.145668	215	H	-0.005280
51	C	-0.133025	106	C	0.000008	161	H	0.004555	216	C	0.117958
52	C	0.201374	107	H	0.000004	162	C	-0.115662	217	H	0.003095
53	C	0.220000	108	H	-0.000001	163	H	-0.005937	218	C	-0.079740
54	C	0.075772	109	H	0.000002	164	C	0.145658			

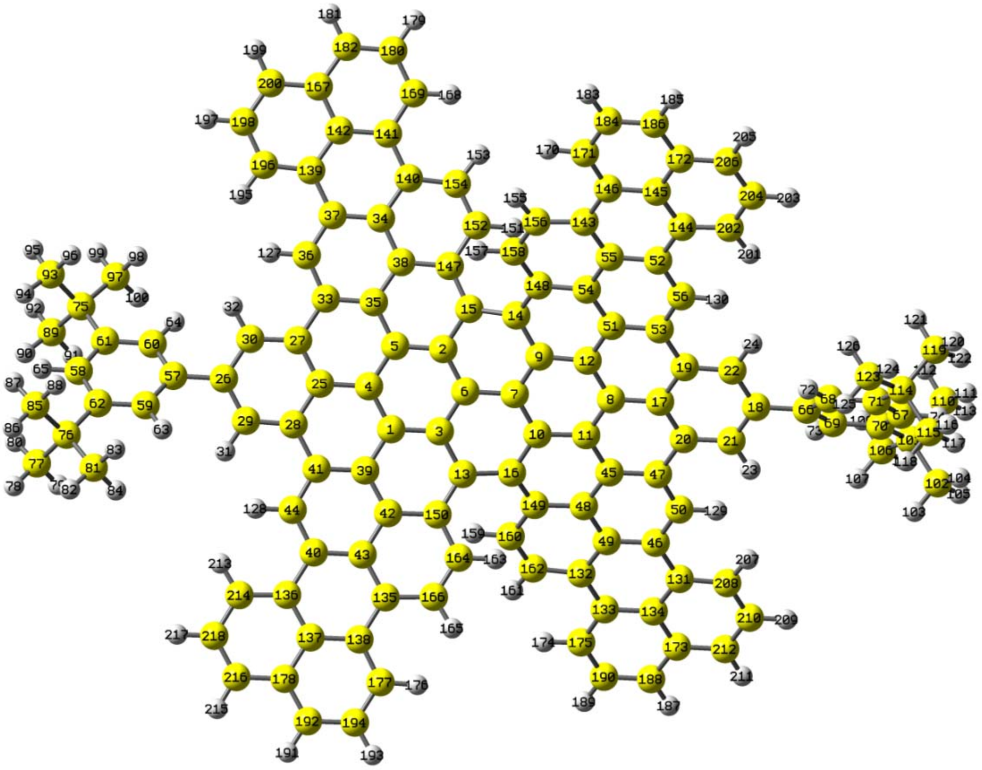

Supplementary Table 14 | Mulliken atomic spin densities of open shell quintet 4.

1	55 C -0.176343	110 C 0.000079	165 H 0.007531
1 C 0.165392	56 C -0.164051	111 H -0.000006	166 C -0.195221
2 C -0.131713	57 C 0.013607	112 H 0.000001	167 C -0.126466
3 C -0.131713	58 C -0.008728	113 H 0.000028	168 H -0.011503
4 C -0.112891	59 C -0.009014	114 C -0.000338	169 C 0.261318
5 C 0.165392	60 C -0.009014	115 C 0.000079	170 H -0.011503
6 C 0.171119	61 C 0.005389	116 H 0.000028	171 C 0.261319
7 C 0.171112	62 C 0.005389	117 H -0.000006	172 C -0.126466
8 C -0.112883	63 H 0.000464	118 H 0.000001	173 C -0.126466
9 C -0.131707	64 H 0.000464	119 C 0.000088	174 H -0.011503
10 C -0.131707	65 H 0.000436	120 H 0.000013	175 C 0.261319
11 C 0.165387	66 C 0.013606	121 H 0.000001	176 H -0.011503
12 C 0.165387	67 C -0.008727	122 H -0.000008	177 C 0.261318
13 C 0.261691	68 C -0.009014	123 C 0.000004	178 C -0.126466
14 C 0.261685	69 C -0.009014	124 H -0.000001	179 H 0.005792
15 C 0.261691	70 C 0.005388	125 H 0.000002	180 C -0.152037
16 C 0.261686	71 C 0.005388	126 H 0.000003	181 H -0.010849
17 C 0.094874	72 H 0.000464	127 H 0.006211	182 C 0.241081
18 C -0.085352	73 H 0.000464	128 H 0.006211	183 H 0.005792
19 C -0.112331	74 H 0.000436	129 H 0.006210	184 C -0.152038
20 C -0.112331	75 C -0.000338	130 H 0.006210	185 H -0.010850
21 C 0.136724	76 C -0.000338	131 C -0.159183	186 C 0.241082
22 C 0.136724	77 C 0.000088	132 C 0.368360	187 H -0.010850
23 H -0.005858	78 H 0.000013	133 C -0.195282	188 C 0.241082
24 H -0.005858	79 H 0.000001	134 C 0.110404	189 H 0.005792
25 C 0.094878	80 H -0.000008	135 C 0.368360	190 C -0.152038
26 C -0.085355	81 C 0.000004	136 C -0.159183	191 H -0.010849
27 C -0.112335	82 H -0.000001	137 C 0.110404	192 C 0.241081
28 C -0.112335	83 H 0.000002	138 C -0.195282	193 H 0.005792
29 C 0.136727	84 H 0.000003	139 C -0.159183	194 C -0.152037
30 C 0.136727	85 C 0.000079	140 C 0.368360	195 H -0.009219
31 H -0.005858	86 H 0.000028	141 C -0.195282	196 C 0.207132
32 H -0.005858	87 H -0.000006	142 C 0.110404	197 H 0.005000
33 C 0.209717	88 H 0.000001	143 C 0.368360	198 C -0.128069
34 C -0.176345	89 C 0.000079	144 C -0.159183	199 H -0.008451
35 C -0.133872	90 H -0.000006	145 C 0.110404	200 C 0.189805
36 C -0.164054	91 H 0.000001	146 C -0.195282	201 H -0.009219
37 C 0.229997	92 H 0.000028	147 C -0.096480	202 C 0.207132
38 C 0.127819	93 C 0.000088	148 C -0.096475	203 H 0.005000
39 C -0.133872	94 H -0.000008	149 C -0.096475	204 C -0.128069
40 C 0.229997	95 H 0.000013	150 C -0.096481	205 H -0.008451
41 C 0.209717	96 H 0.000001	151 H -0.010475	206 C 0.189805
42 C 0.127819	97 C 0.000004	152 C 0.260199	207 H -0.009219
43 C -0.176345	98 H 0.000003	153 H 0.007531	208 C 0.207132
44 C -0.164055	99 H -0.000001	154 C -0.195221	209 H 0.005000

45	C	-0.133868	100	H	0.000002	155	H	0.007530	210	C	-0.128069
46	C	0.229996	101	C	-0.000338	156	C	-0.195220	211	H	-0.008451
47	C	0.209714	102	C	0.000088	157	H	-0.010475	212	C	0.189805
48	C	0.127815	103	H	0.000001	158	C	0.260198	213	H	-0.009219
49	C	-0.176343	104	H	-0.000008	159	H	-0.010475	214	C	0.207132
50	C	-0.164051	105	H	0.000013	160	C	0.260197	215	H	-0.008451
51	C	-0.133868	106	C	0.000004	161	H	0.007530	216	C	0.189805
52	C	0.229996	107	H	0.000003	162	C	-0.195220	217	H	0.005000
53	C	0.209714	108	H	-0.000001	163	H	-0.010475	218	C	-0.128069
54	C	0.127815	109	H	0.000002	164	C	0.260199			

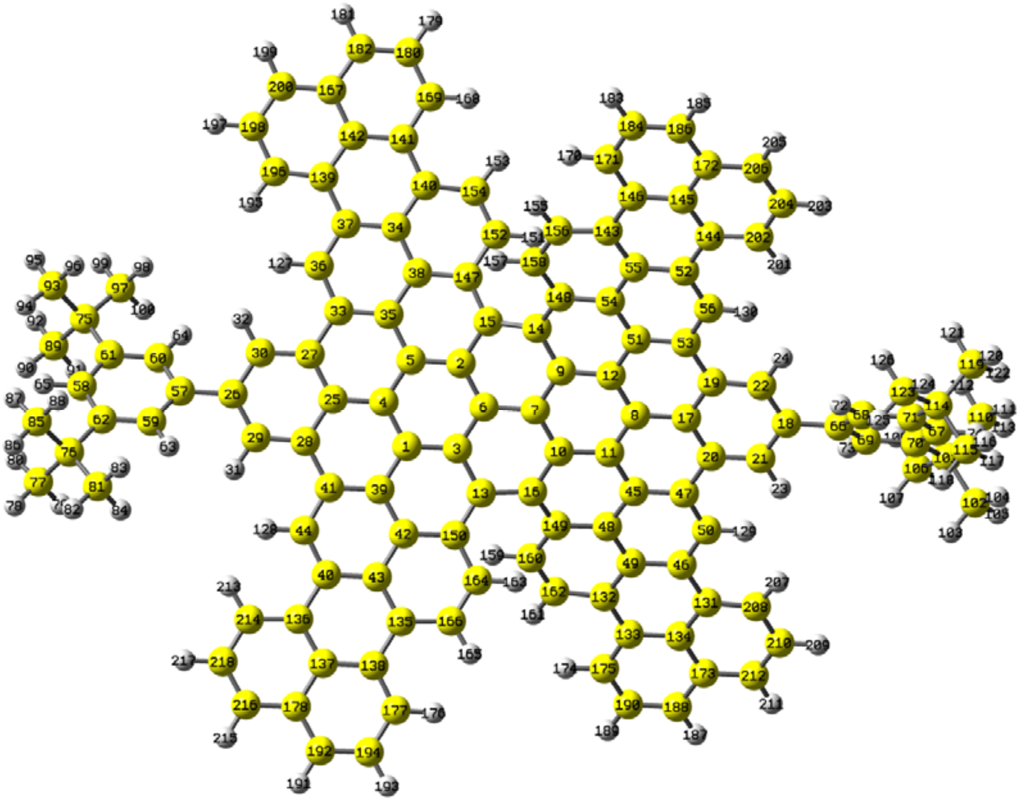

Supplementary Discussion

Conformational flexibility of the 2D acenes in solution

Although frozen in a single conformation as a result of crystal packing constraints in the solid state, these 2D acenes are highly flexible in solution and change their shape constantly upon heating. For example, the ^{1}H NMR spectra of **1g**, as a representative tetracene-dibenzoterrylene, revealed evident π–π stacking upon cooling the solution from 378 to 298 K, with the protons of the central benzo group shifting from 6.60 to 5.70 ppm (Supplementary Fig. 72b); upon further cooling, half of these upfield shifted protons shifted to 7.49 ppm, due to intermolecular C–F···H–C hydrogen bonds formed in the cove region (F···H bond lengths: 2.31–2.53 Å), strengthened by the freezing of the π-dimer (Supplementary Fig. 72a). The central benzo group in the middle of the molecule was structurally distinct, occupying a unique environment, in each of the five conformations. When unconstrained in solution, the interconversion among these conformations rendered them time-averaged and equivalent. Evidence for rapid equalization is evident in the ^{1}H NMR spectra of **3**. The signal for the central benzo group appears as only one coalesced broad signal at 6.70 ppm, as expected, at 378 K (Supplementary Fig. 72c); as the temperature decreased, bond rotations become slower on the NMR spectroscopic timescale, causing the coalesced signals to broaden and split into five singlets at 6.77, 6.47, 6.35, 6.22, and 5.61 ppm.

The missing fjord-region protons of*c*TT-**2** appeared (at 9.42, 8.38, and 7.76 ppm) upon cooling to 278 K; the signals of other conformers (marked by asterisks in Supplementary Fig. 72d) appeared upon heating a solution of *c*TT-**2** in TCE. Although *s*TT-**2** is more stable than the other conformers by 6.91–19.46 kJ/mol (Supplementary Table 1), the other conformers are also kinetically stable because their interconversion requires overcoming large energy barriers. We could not, however, obtain thermodynamic or kinetic information for their thermal isomerization processes because of undefined product conformations.

Supplementary Methods

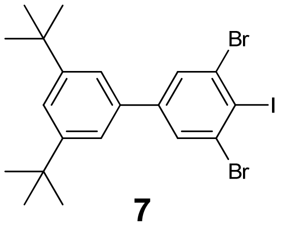

7

Synthesis of 3, 5-dibromo-3',5'-di-tert-butyl-4-iodobiphenyl 7: 3',5'-di-tert-butylbiphenyl-4-amine **5** (37.6 g, 134 mmol) was dissolved in 100 mL CH_2Cl_2/ MeOH (1:1). To this was added Br_2 (300 mmol) in 100 mL CH_2Cl_2 / MeOH (1:1). The mixture was stirred at room temperature for 10 hours. The solution was concentrated, the residue was taken up in CH_2Cl_2 (200 mL), and washed sequentially with 1 N NaOH (200 mL), saturated $Na_2S_2O_3$ solution, a saturated $NaHCO_3$ solution, water and brine. The organic phase was dried, concentrated to give **6** for next step without purification. $NaNO_2$ (8.97 g, 130 mmol) was slowly added at 0 °C to a suspension of compound **6** in concentrated sulfuric acid (50 mL) and HAc (250 mL). After additional stirring for 6h at 0 °C, the black viscous mixture was slowly added to the solution of KI (42.9 g, 260 mmol) and I_2 (12.6 g, 50 mmol) in water at 0 °C. After complete addition, most of the solvent was evaporated and the mixture was poured into dichloromethane/water (500/500 mL). The organic phase was extracted with CH_2Cl_2, washed with a saturated $Na_2S_2O_3$ solution, a saturated $NaHCO_3$ solution, water and brine, and dried with $MgSO_4$. The crude residue was subjected to flash silica gel column chromatography (CH_2Cl_2/petroleum ether 1:15) to yield solid **7** as off white powder (71 %).

^{1}H NMR (400 MHz, $CDCl_3$) δ ppm 7.79 (s, 2 H), 7.52 (s, 1 H), 7.34 (d, J = 1.6 Hz, 2 H), 1.41 (s, 18 H); See Supplementary Fig. 1. **^{13}C NMR (100 MHz, $CDCl_3$)** δ ppm151.88, 145.41, 137.33, 131.53, 130.13, 122.94, 121.48, 107.00, 35.17, 31.65; See Supplementary Fig. 2. **HRMS (m/z)**: $[M]^+$ calcd. for $C_{20}H_{23}Br_2I$, 547.9211; found, 547.9208; See Supplementary Fig. 3.

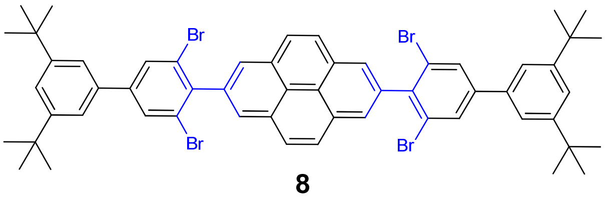

8

Synthesis of 2,7-bis(3,5-dibromo-3',5'-di-tert-butylbiphenyl-4-yl)pyrene 8: A mixture of **7** (2.74 g, 5 mmol), 2,7-bis(4,4,5,5-tetramethyl-1,3,2-dioxaborolan-2-yl)pyrene (0.91 g, 2 mmol), $Pd(PPh_3)_4$ (116 mg, 0.10 mmol) and Na_2CO_3 aqueous solution (2 M, 10 mL) in THF (80 mL) was degassed. The reaction mixture was vigorously stirred under nitrogen at 80°C for 36 h. After cooling to room temperature, water (30 mL) was added. The resultant suspension was extracted with dichloromethane (3×50 mL). The combined organic layers were washed with water and then dried over anhydrous $MgSO_4$. The solvent was removed and the crude product was purified by column chromatography (silica gel, petroleum ether) to afford compound **8** (1.5 g) as a white powder in 72% yield.

^{1}H NMR (400 MHz, $CDCl_3$) δ ppm 8.19 (s, 4 H), 8.12 (s, 4 H), 7.94 (s, 4 H), 7.53 (s, 2 H), 7.45 (s, 4 H), 1.43 (s, 36 H) ; See Supplementary Fig. 4.**^{13}C NMR (125 MHz, $CDCl_3$)** δ ppm 151.71, 144.91, 141.39, 138.90, 137.68, 131.31, 130.75, 127.92, 126.05, 124.90, 124.17, 122.69, 121.63, 35.11, 31.61, 31.57; See Supplementary Fig. 5. **HRMS (m/z):** $[M]^+$ calcd. for $C_{56}H_{54}Br_4$, 1042.0954, found, 1042.0961; See Supplementary Fig. 6.

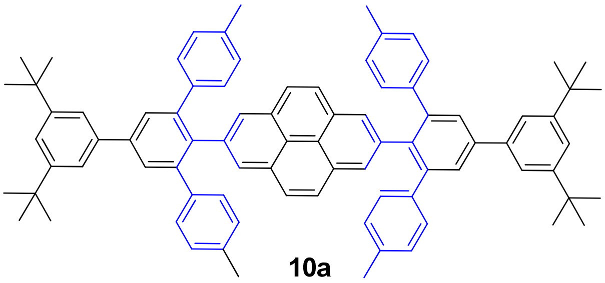

10a

Synthesis of 10a: A mixture of **8** (208 mg, 0.2 mmol), p-tolylboronic acid **9a** (136 mg, 1 mmol), $Pd(PPh_3)_4$ (23.2 mg, 0.02 mmol) and Na_2CO_3 aqueous solution (2 M, 2 mL) in toluene (30 mL) and EtOH (10 mL) was degassed. The reaction mixture was vigorously stirred under nitrogen at 80°C for 24 h. After cooling to room temperature, water (30 mL) was added. The resultant suspension was extracted with dichloromethane (3×30 mL). The combined organic layers were washed with water and then dried over anhydrous $MgSO_4$. The solvent was removed and the crude product was purified by column chromatography (silica gel, DCM/ petroleum ether= 1/3) to afford compound **10a** (172 mg) as a white powder in 79% yield.

^{1}H NMR (400 MHz, $CDCl_3$) δ ppm 7.69 (s, 4 H), 7.66 (s, 4 H), 7.59 (s, 4 H), 7.52 (s, 4 H), 7.48 (s, 2 H), 7.02 (d, *J* = 8.0 Hz, 8 H), 6.86 (d, *J* = 7.9 Hz, 8 H), 2.18 (s, 12 H), 1.39 (s, 36 H); See Supplementary Fig. 7. **^{13}C NMR (125 MHz, $CDCl_3$)** δ ppm 151.27, 142.79, 141.71, 140.19, 139.22, 137.64, 137.05, 135.74, 130.20, 129.94, 128.96, 128.46, 127.07, 122.78, 121.87, 121.68, 35.06, 31.59, 21.03 ; See Supplementary Fig. 8. **HRMS (m/z)**: $[M]^+$ calcd. For $C_{84}H_{82}$, 1090.6411; found, 1090.6402; See Supplementary Fig. 9.

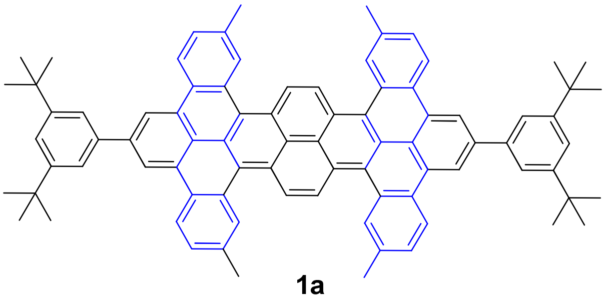

1a

Synthesis of 1a: To a stirred solution of **10a** (109 mg, 0.1 mmol) in dichloromethane (50 mL), a solution of iron(III) chloride (161 mg, 1 mmol) in nitromethane (10 mL) was added dropwise. A nitrogen stream was bubbled through the mixture throughout the course of the reaction. After stirring for another 2 hours at 40°C, the reaction was quenched with methanol (10 mL). The resulting mixture was poured into water and extracted with DCM. The extract was purified by column chromatography (DCM/petroleum ether = 1/3) to afford **1a** (87 mg, 81%) as an orange powder. mp >300°C.

^{1}H NMR (400 MHz, $CDCl_3$) δ ppm 9.31 (s, 4 H), 9.12 (s, 4 H), 9.06 (s, 4 H), 8.84 (d, *J* = 8.4 Hz, 4 H), 7.82 (s, 4 H), 7.69 (d, *J* = 8.0 Hz, 4 H), 7.62 (s, 2 H), 2.81 (s, 12 H), 1.53 (s, 36 H); See Supplementary Fig.10. ^{13}C NMR spectra cannot be obtained due to poor solubility. **HRMS (m/z)**: $[M]^+$ calcd. for $C_{84}H_{74}$, 1082.5785; found, 1082.5783; See Supplementary Fig. 11.

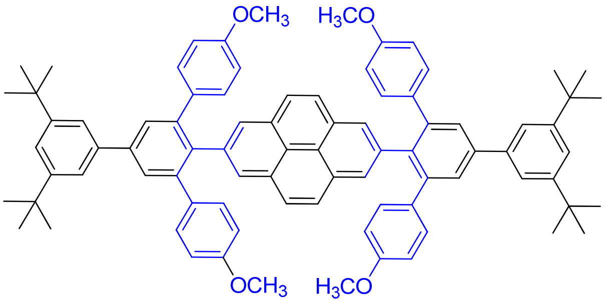

10b

Synthesis of 10b: A mixture of **8** (208 mg, 0.2 mmol), (4-methoxyphenyl)boronic acid **9b** (152 mg, 1 mmol), $Pd(PPh_3)_4$ (23.2 mg, 0.02 mmol) and Na_2CO_3 aqueous solution (2 M, 2 mL) in toluene (30 mL) and EtOH (10 mL) was degassed. The reaction mixture was vigorously stirred under nitrogen at 80°C for 24 h. After cooling to room temperature, water (30 mL) was added. The resultant suspension was extracted with dichloromethane (3×30 mL). The combined organic layers were washed with water and then dried over anhydrous $MgSO_4$. The solvent was removed and the crude product was purified by column chromatography (silica gel, DCM/ petroleum ether = 1/3) to afford compound **10b** (175 mg) as a white powder in 76% yield.

^{1}H NMR (400 MHz, $CDCl_3$) δ ppm 7.68 (s, 4 H), 7.65 (s, 4 H), 7.59 (s, 4 H), 7.53 (d, *J* = 1.6 Hz, 4 H), 7.49 (s, 2 H), 7.04 (d, *J* = 8.7 Hz, 8 H), 6.58 (d, *J* = 8.7 Hz, 8 H), 3.64 (s, 12 H), 1.40 (s, 36 H); See Supplementary Fig. 12. **^{13}C NMR (100 MHz, $CDCl_3$)** δ ppm 158.20, 151.59, 142.73, 142.12, 140.56, 137.98, 137.49, 134.79, 131.45, 130.60, 129.12, 128.81, 127.45, 123.01, 122.19, 122.00, 113.50, 55.33, 35.37, 31.91; See Supplementary Fig. 13. **HRMS (m/z)**: $[M]^+$ calcd. for $C_{84}H_{82}O_4$, 1154.6208; found, 1154.6189; See Supplementary Fig. 14.

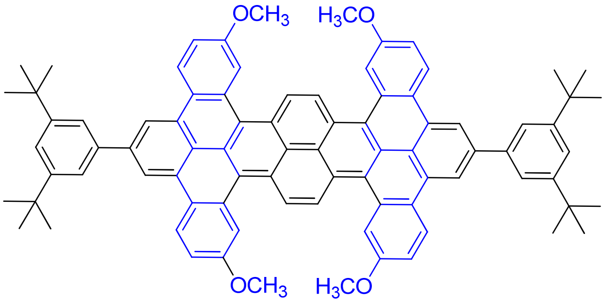

1b

Synthesis of 1b: To a stirred solution of **10b** (115 mg, 0.1 mmol) in dichloromethane (50 mL), a solution of iron(III) chloride (161 mg, 1 mmol) in nitromethane (10 mL) was added dropwise. A nitrogen stream was bubbled through the mixture throughout the course of the reaction. After stirring for another 2 hours at 40°C, the reaction was quenched with methanol (10 mL). The resulting mixture was poured into water and extracted with DCM. The extract was purified by column chromatography (DCM/petroleum ether = 1/3) to afford **1b** (80 mg, 70%) as an orange powder. mp >300°C.

^{1}H NMR (500 MHz, $CDCl_2CDCl_2$, 378K) δ ppm 9.41 – 9.31 (m, 3 H), 9.30 – 9.19 (m, 3 H), 9.09 (s, 1 H), 9.07 – 9.00 (m, 3 H), 8.94 – 8.89 (m, 3 H), 8.88 – 8.84 (m, 2 H), 8.46 – 8.41 (m, 1 H), 7.83 (s, 4 H), 7.63 (s, 2 H), 7.53 – 7.49 (m, 4 H), 4.20 (m, 6 H), 4.17 (m, 6 H), 1.56 (s, 36 H); See Supplementary Fig. 15. **^{13}C NMR (125 MHz, $CDCl_2CDCl_2$, 378 K)** δ ppm 159.52, 158.76, 151.65, 141.77, 141.63, 141.42, 141.33, 132.56, 131.91, 130.48, 126.07, 125.99, 125.67, 122.32, 121.95, 120.71, 120.23, 119.63, 116.44, 115.81, 114.84, 114.20, 107.86, 100.00, 55.87, 55.73, 35.10, 31.72, 30.48; See Supplementary Fig. 16. **HRMS (m/z)**: $[M]^+$ calcd. for $C_{84}H_{82}O_4$, 1146.5582; found, 1146.5580; See Supplementary Fig. 17.

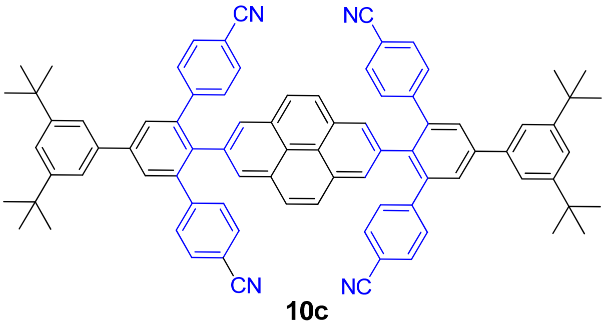

10c

Synthesis of 10c: A mixture of **8** (208 mg, 0.2 mmol), (4-cyanophenyl)boronic acid **9c** (147 mg, 1 mmol), $Pd(PPh_3)_4$ (23.2 mg, 0.02 mmol) and Na_2CO_3 aqueous solution (2 M, 2 mL) in toluene (30 mL) and EtOH (10 mL) was degassed. The reaction mixture was vigorously stirred under nitrogen at 80°C for 24 h. After cooling to room temperature, water (30 mL) was added. The resultant suspension was extracted with dichloromethane (3×30 mL). The combined organic layers were washed with water and then dried over anhydrous $MgSO_4$. The solvent was removed and the crude product was purified by column chromatography (silica gel, DCM/ petroleum ether = 1/3) to afford compound **10c** (174 mg) as a white powder in 75% yield.

^{1}H NMR (400 MHz, $CDCl_3$) δ ppm 7.73 (s, 4 H), 7.61 (s, 4 H), 7.57 (s, 4 H), 7.54 (s, 2 H), 7.50 (s, 4 H), 7.37 (d, J = 8.1 Hz, 8 H), 7.24 (d, J = 8.1 Hz, 8 H), 1.41 (s, 36 H); See Supplementary Fig. 18. **^{13}C NMR (125 MHz, $CDCl_3$)** δ ppm 151.40, 142.80, 142.05, 140.12, 139.87, 137.78, 136.64, 133.21, 131.77, 130.28, 129.69, 128.66, 128.42, 128.37, 127.88, 127.55, 127.07, 126.79, 125.84, 125.66, 122.69, 121.89, 35.07, 31.58; See Supplementary Fig. 19. **HRMS (m/z)**: $[M+Na]^+$ calcd. for $C_{84}H_{70}N_4Na$, 1157.5493; found, 1157.5488; See Supplementary Fig. 20.

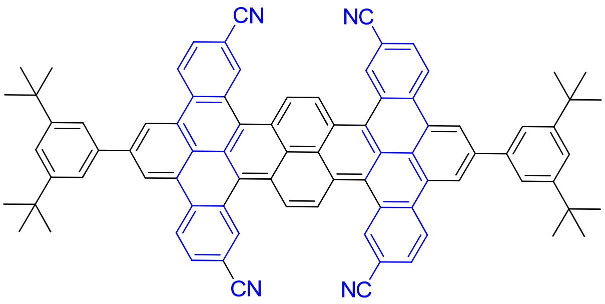

1c

Synthesis of 1c: To a stirred solution of **10c** (116 mg, 0.1 mmol) in dichloromethane (50 mL), a solution of iron(III) chloride (161 mg, 1 mmol) in nitromethane (10 mL) was added dropwise. A nitrogen stream was bubbled through the mixture throughout the course of the reaction. After stirring for another 2 hours at 40°C, the reaction was quenched with methanol (10 mL). The resulting mixture was poured into water and extracted with DCM. The extract was purified by column chromatography (DCM/petroleum ether = 1/3) to afford **1c** (83 mg, 72%) as an red powder. mp >300°C.

^{1}H NMR (400 MHz, CDCl$_3$) δ ppm9.37 (s, 4 H), 9.23 (s, 4 H), 9.04 (s, 4 H), 8.92 (d, J = 8.4 Hz, 4 H), 8.06 (d, J = 8.4 Hz, 4 H), 7.73 (s, 4 H), 7.65 (s, 2 H), 1.52 (s, 36 H); See Supplementary Fig. 21. **^{13}C NMR (100 MHz, CDCl$_3$)** δ ppm 151.97, 142.76, 140.17, 134.94, 134.28, 130.19, 128.62, 127.54, 127.21, 125.81, 125.37, 124.65, 124.29, 123.30, 122.75, 122.67, 122.48, 118.87, 111.04, 35.20, 31.73; See Supplementary Fig. 22. **HRMS (m/z)**: $[M]^+$ calcd. for $C_{84}H_{62}N_4$, 1149.4867; found, 1149.4857; See Supplementary Fig. 23.

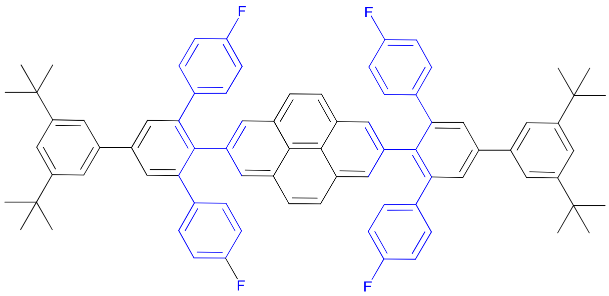

10d

Synthesis of 10d: A mixture of **8** (208 mg, 0.2 mmol), 4-fluorophenylboronic acid **9d** (140 mg, 1 mmol), $Pd(PPh_3)_4$ (23.2 mg, 0.02 mmol) and Na_2CO_3 aqueous solution (2 M, 2 mL) in toluene (30 mL) and EtOH (10 mL) was degassed. The reaction mixture was vigorously stirred under nitrogen at 80°C for 24 h. After cooling to room temperature, water (30 mL) was added. The resultant suspension was extracted with dichloromethane (3×30 mL). The combined organic layers were washed with water and then dried over anhydrous $MgSO_4$. The solvent was removed and the crude product was purified by column chromatography (silica gel, DCM/ petroleum ether = 1/3) to afford compound **10d** (192 mg) as a white powder in 87% yield.

^{1}H NMR (400 MHz, $CDCl_3$) δ ppm 7.69 (s, 4 H), 7.61 (s, 4 H), 7.61 (s, 4 H), 7.53 (d, *J* = 1.7 Hz, 4 H), 7.51 (d, *J* = 1.7 Hz, 2 H), 7.08 (dd, *J* = 8.7, 5.4 Hz, 8 H), 6.75 (t, *J* = 8.7 Hz, 8 H), 1.41 (s, 36 H); See Supplementary Fig. 24. **^{13}C NMR (100 MHz, $CDCl_3$)** δ ppm 160.31, 151.47, 142.13, 142.01, 139.96, 137.89 (d, $^4J_{C\text{-}F}$ = 2.8 Hz), 136.79, 131.64 (d, $^3J_{C\text{-}F}$ = 7.8 Hz), 130.47, 129.11, 128.48, 127.37, 122.40 (d, $^1J_{C\text{-}F}$ = 80.4 Hz), 121.90, 114.82 (d, $^2J_{C\text{-}F}$ = 21.3 Hz), 35.12, 31.79, 31.68; See Supplementary Fig. 25. **^{19}F NMR (471 MHz, $CDCl_3$)** δ ppm -113.66; See Supplementary Fig. 26. **HRMS (m/z)**: $[M]^+$ calcd. for $C_{80}H_{70}F_4$, 1106.5408; found, 1106.5407; See Supplementary Fig. 27.

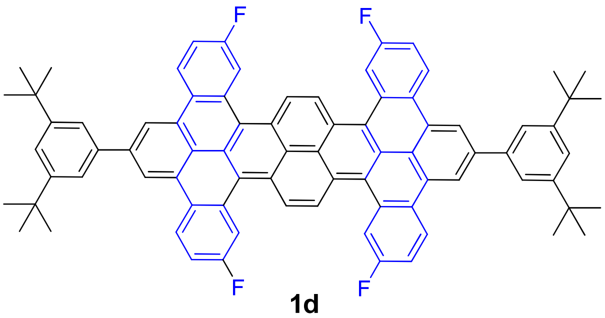

1d

Synthesis of 1d: To a stirred solution of **10d** (110 mg, 0.1 mmol) in dichloromethane (50 mL), a solution of iron(III) chloride (161 mg, 1 mmol) in nitromethane (10 mL) was added dropwise. A nitrogen stream was bubbled through the mixture throughout the course of the reaction. After stirring for another 2 hours at 40°C, the reaction was quenched with methanol (10 mL). The resulting mixture was poured into water and extracted with DCM. The extract was purified by column chromatography (DCM/petroleum ether = 1/3) to afford **1d** (80.2 mg, 73%) as an orange powder.mp >300°C.^{1}H NMR spectra showed low resolution due to aggregation.

^{1}H NMR (500 MHz, $CDCl_2CDCl_2$, 378 K) δ ppm 8.92 (broad), 7.80 (s, 4 H), 7.64 (s, 2 H), 1.55 (s, 36 H) ; See Supplementary Fig. 28. **^{13}C NMR (126 MHz, $CDCl_2CDCl_2$, 378 K)** δ 162.55 (s), 160.60 (s), 151.51 (s), 142.11 (s), 140.63 (s), 131.76 (d, $^{3}J_{C-F}$ = 8.0 Hz), 129.40 (s), 127.93 (s), 126.41 (d, $^{4}J_{C-F}$ = 6.6 Hz), 126.39 (s), 126.25 (d, $^{3}J_{C-F}$ = 8.4 Hz), 125.16 (s), 124.16 (s), 123.37 (s), 122.21 (d, $^{1}J_{C-F}$ = 60 Hz), 122.02 (s), 120.45 (s), 116.07 (d, $^{2}J_{C-F}$ = 23.2 Hz), 115.58 (d, $^{2}J_{C-F}$ = 22.9 Hz), 34.88 (s), 31.44 (d, J = 13.5 Hz); See Supplementary Fig. 29. **^{19}F NMR (471 MHz, $CDCl_2CDCl_2$)** δ -113.48; See Supplementary Fig. 30. **HRMS (m/z)**: $[M]^+$ calcd. for $C_{80}H_{62}F_4$,1098.4782; found, 1098.4781; See Supplementary Fig. 31.

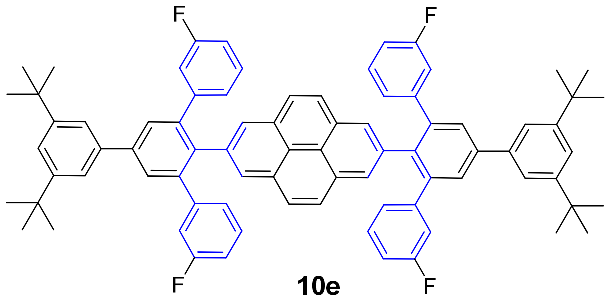

10e

Synthesis of 10e: A mixture of **8** (208 mg, 0.2 mmol), 3-fluorophenylboronic acid **9e** (140 mg, 1 mmol), $Pd(PPh_3)_4$ (23.2 mg, 0.02 mmol) and Na_2CO_3 aqueous solution (2 M, 2 mL) in toluene (30 mL) and EtOH (10 mL) was degassed. The reaction mixture was vigorously stirred under nitrogen at 80°C for 24 h. After cooling to room temperature, water (30 mL) was added. The resultant suspension was extracted with dichloromethane (3×30 mL). The combined organic layers were washed with water and then dried over anhydrous $MgSO_4$. The solvent was removed and the crude product was purified by column chromatography (silica gel, DCM/ petroleum ether = 1/3) to afford compound **10e** (199 mg) as a white powder in 90% yield.

^{1}H NMR (400 MHz, $CDCl_3$) δ ppm 7.70 (s, 4 H), 7.63 (s, 4 H), 7.62 (s, 4 H), 7.52 (s, 4 H), 7.51 (s, 2 H), 6.95 (dd, J = 13.4, 8.1 Hz, 8 H), 6.80 – 6.72 (m, 8 H), 1.41 (s, 36 H); See Supplementary Fig.32. **^{13}C NMR (125 MHz, $CDCl_3$)** δ ppm 163.23, 161.27, 151.47, 144.09(d, $^{3}J_{C\text{-}F}$ = 7.5 Hz), 142.07, 141.79, 139.69, 137.58, 136.20, 130.40, 129.14 (d, $^{3}J_{C\text{-}F}$ = 7.7 Hz),129.13, 128.16, 127.22, 125.97(d, $^{4}J_{C\text{-}F}$ = 2.1 Hz), 122.41(d, $^{1}J_{C\text{-}F}$ = 100.5 Hz), 121.80, 116.87 (d, $^{2}J_{C\text{-}F}$ = 21.6 Hz), 113.33(d, $^{2}J_{C\text{-}F}$ = 20.9 Hz), 35.08, 31.58; See Supplementary Fig. 33. **^{19}F NMR (471 MHz, $CDCl_3$)** δ ppm -113.66; See Supplementary Fig. 34. **HRMS (m/z)**: $[M]^+$ calcd. for $C_{80}H_{70}F_4$, 1106.5408; found, 1106.5397; See Supplementary Fig. 35.

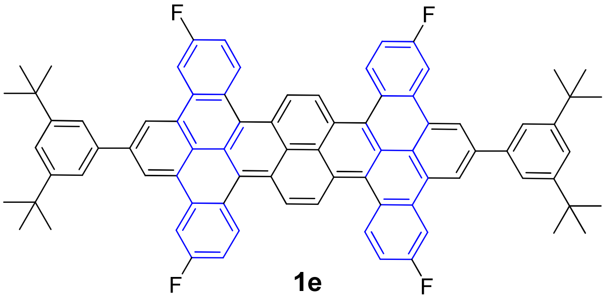

1e

Synthesis of 1e: To a stirred solution of **10e** (110 mg, 0.1 mmol) in dichloromethane (50 mL), a solution of iron(III) chloride (161 mg, 1 mmol) in nitromethane (10 mL) was added dropwise. A nitrogen stream was bubbled through the mixture throughout the course of the reaction. After stirring for another 2 hours at 40°C, the reaction was quenched with methanol (10 mL). The resulting mixture was poured into water and extracted with DCM. The extract was purified by column chromatography (DCM/petroleum ether = 1/3) to afford **1e** (85 mg, 77%) as an orange powder. mp >300°C.

^{1}H NMR and ^{13}C NMR spectra showed low resolution due to aggregation. **^{19}F NMR (471 MHz, $CDCl_2CDCl_2$)** δ -112.62; See Supplementary Fig. 36. **HRMS (m/z)**: $[M]^+$ calcd. for $C_{80}H_{62}F_4$, 1098.4782 ; found, 1098.4774; See Supplementary Fig. 37.

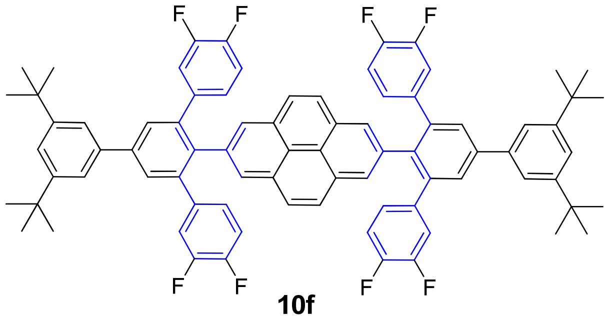

10f

Synthesis of 10f: A mixture of **8** (208 mg, 0.2 mmol), (3,4-difluorophenyl)boronic acid **9f** (158 mg, 1 mmol), $Pd(PPh_3)_4$ (23.2 mg, 0.02 mmol) and Na_2CO_3 aqueous solution (2 M, 2 mL) in toluene (30 mL) and EtOH (10 mL) was degassed. The reaction mixture was vigorously stirred under nitrogen at 80°C for 24 h. After cooling to room temperature, water (30 mL) was added. The resultant suspension was extracted with dichloromethane (3×30 mL). The combined organic layers were washed with water and then dried over anhydrous $MgSO_4$. The solvent was removed and the crude product was purified by column chromatography (silica gel, DCM/ petroleum ether= 1/3) to afford compound **10f** (205 mg) as a white powder in 87% yield.

^{1}H NMR (400 MHz, CDCl$_3$) δ ppm 7.69 (s, 4 H), 7.66 (s, 4 H), 7.64 (s, 4 H), 7.52 (d, J = 1.4 Hz, 2 H), 7.51 (s, 4 H), 7.10 – 7.00 (m, 4 H), 6.83 – 6.69 (m, 8 H), 1.41 (s, 36 H) ; See Supplementary Fig. 38. **^{13}C NMR (125 MHz, CDCl$_3$)** δ ppm 151.70, 150.51 (dd, $^{1,2}J_{C\text{-}F}$ = 70.6, 15.1 Hz), 148.53 (dd, $^{1,2}J_{C\text{-}F}$ = 73.2, 12.7 Hz), 142.46, 141.15, 139.64, 138.76(t, $^3J_{C\text{-}F}$ = 5.1 Hz), 137.70, 136.24, 130.72, 129.39, 128.20, 127.57, 126.35 (dd, $^{3,4}J_{C\text{-}F}$ = 5.5, 3.5 Hz), 122.94, 122.30, 121.90, 118.94 (d, $^2J_{C\text{-}F}$ = 17.4 Hz), 116.78 (d, $^2J_{C\text{-}F}$ = 17.1 Hz), 35.22, 31.71; See Supplementary Fig. 39. **^{19}F NMR (471 MHz, CDCl$_3$)** δ ppm -113.65 (s), -138.12 (d, J = 21.5 Hz), -140.41 (d, J = 21.6 Hz); See Supplementary Fig. 40. **HRMS (m/z)**: $[M]^+$ calcd. for $C_{80}H_{66}F_8$, 1178.5031; found, 1178.5023; See Supplementary Fig. 41.

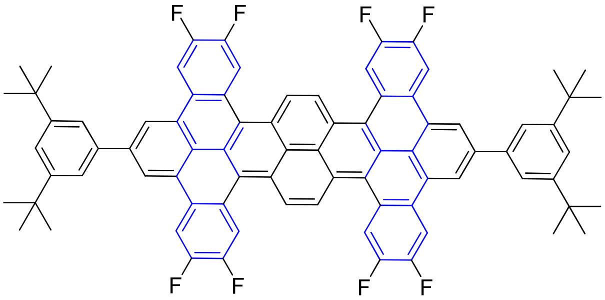

1f

Synthesis of 1f: To a stirred solution of **10f** (118 mg, 0.1 mmol) in dichloromethane (50 mL), a solution of iron(III) chloride (161 mg, 1 mmol) in nitromethane (10 mL) was added dropwise. A nitrogen stream was bubbled through the mixture throughout the course of the reaction. After stirring for another 2 hours at 40°C, the reaction was quenched with methanol (10 mL). The resulting mixture was poured into water and extracted with DCM. The extract was purified by column chromatography (DCM/petroleum ether = 1/3) to afford **1f** (82 mg, 70%) as an orange powder.mp >300°C.

^{1}H NMR and ^{13}C NMR spectra showed low resolution due to aggregation. **^{19}F NMR (471 MHz, $CDCl_3$)** δ -136.73, -137.80; See Supplementary Fig. 42. **HRMS (m/z)**: $[M]^{+}$ calcd. for $C_{80}H_{58}F_8$, 1170.4405; found, 1170.4393; See Supplementary Fig. 43.

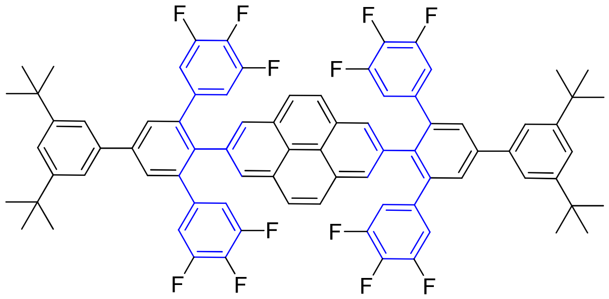

10g

Synthesis of 10g: A mixture of **8** (208 mg, 0.2 mmol), (3,4,5-trifluorophenyl)boronic acid **9g** (176 mg,1 mmol), $Pd(PPh_3)_4$ (23.2 mg, 0.02 mmol) and Na_2CO_3 aqueous solution (2 M, 2 mL) in toluene (30 mL) and EtOH (10 mL) was degassed. The reaction mixture was vigorously stirred under nitrogen at 80°C for 24 h. After cooling to room temperature, water (30 mL) was added. The resultant suspension was extracted with dichloromethane (3×30 mL). The combined organic layers were washed with water and then dried over anhydrous $MgSO_4$. The solvent was removed and the crude product was purified by column chromatography (silica gel, DCM/ petroleum ether= 1/3) to afford compound **10g** (170 mg) as a white powder in 68% yield.

^{1}H NMR (500 MHz, CDCl$_3$) δ 7.79 (s, 4 H), 7.68 (s, 4 H), 7.64 (s, 4 H), 7.54 (t, J = 1.7 Hz, 2 H), 7.49 (d, J = 1.8 Hz, 4 H), 6.74 (dd, J = 8.0, 6.6 Hz, 8 H), 1.42 (s, 36 H) ; See Supplementary Fig. 44. **^{13}C NMR (126 MHz, CDCl$_3$)** δ 151.83, 150.65 (dd, $^{1,2}J_{C\text{-}F}$ = 250.4, 6.1 Hz), 142.73, 140.46, 139.28 (s), 138.81 (dt, $^{1,2}J_{C\text{-}F}$ = 252.0, 15.1 Hz), 137.62 (d, $^2J_{C\text{-}F}$ = 15.0 Hz), 135.62, 130.98, 129.52, 127.83, 127.80, 123.10, 122.52, 121.86, 114.23 (dt, $^{2,3}J_{C\text{-}F}$ = 17.6, 5.0 Hz), 35.24, 31.70; See Supplementary Fig. 45. **^{19}F NMR (471 MHz, CDCl$_3$)** δ ppm -134.59 (d, J = 20.7 Hz), -162.39 (s); See Supplementary Fig. 46. **HRMS (m/z)**: $[M]^+$ calcd. for $C_{80}H_{62}F_{12}$, 1250.4654; found, 1250.4659; See Supplementary Fig. 47.

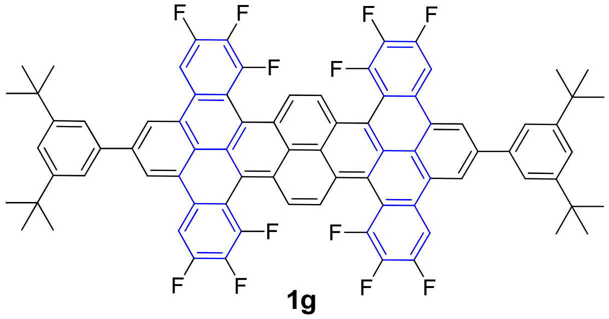

1g

Synthesis of 1g: To a stirred solution of **10g** (125 mg, 0.1 mmol) in dichloromethane (50 mL), a solution of iron(III) chloride (161 mg, 1 mmol) in nitromethane (10 mL) was added dropwise. A nitrogen stream was bubbled through the mixture throughout the course of the reaction. After stirring for another 2 hours at 40°C, the reaction was quenched with methanol (10 mL). The resulting mixture was poured into water and extracted with DCM. The extract was purified by column chromatography (DCM/petroleum ether = 1/3) to afford **1g** (104 mg, 84%) as an orange powder. mp >300°C.

^{1}H NMR (500 MHz, 378K, $CDCl_2CDCl_2$) δ ppm 8.86 (s, 4 H), 8.33 (s, 4 H), 7.97 (s, 4 H), 7.78 (s, 2 H), 6.61 (s, 4 H), 1.67 (d, J = 2.7 Hz, 36 H); See Supplementary Fig. 48. **^{13}C NMR (126 MHz, $CDCl_2CDCl_2$, 378 K)** δ 152.02 (s), 150.58 (dd, $^{1,2}J_{C\text{-}F}$ = 252.0, 9.6 Hz), 148.37 (dd, $^{1,2}J_{C\text{-}F}$ = 258.3, 9.4 Hz), 143.51 (s), 139.30 (dt, $^{1,2}J_{C\text{-}F}$ = 253.2, 16.0 Hz), 139.28 (s), 127.07 (s), 126.58 (s), 124.88 (s), 124.54 (d, $^2J_{C\text{-}F}$ = 11.6 Hz), 124.21 (s), 122.69 (s), 121.82 (s), 121.71 (s), 121.27 (s), 116.84 (s), 115.71 (d, $^2J_{C\text{-}F}$ = 8.1 Hz), 107.00 (d, $^2J_{C\text{-}F}$ = 18.6 Hz), 34.95 (s), 31.42 (s); See Supplementary Fig. 49. **^{19}F NMR (471 MHz, $CDCl_2CDCl_2$)** δ -122.95, -132.16, -158.63; See Supplementary Fig. 50. **HRMS (m/z)**: $[M]^+$ calcd. for $C_{80}H_{54}F_{12}$, 1242.4028; found, 1242.4028; See Supplementary Fig. 51.

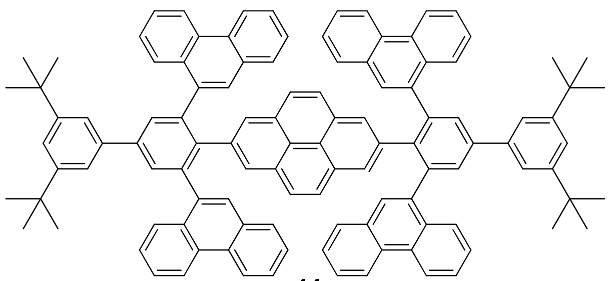

11

Synthesis of 11: A mixture of **8** (208 mg, 0.2 mmol), phenanthren-9-ylboronic acid (222 mg, 1 mmol), $Pd(PPh_3)_4$ (23.2 mg, 0.02 mmol) and Na_2CO_3 aqueous solution (2 M, 2 mL) in toluene (30 mL) and EtOH (10 mL) was degassed. The reaction mixture was vigorously stirred under nitrogen at 80°C for 24 h. After cooling to room temperature, water (30 mL) was added. The resultant suspension was extracted with dichloromethane (3×30 mL). The combined organic layers were washed with water and then dried over anhydrous $MgSO_4$. The solvent was removed and the crude product was purified by column chromatography (silica gel, DCM/ petroleum ether = 1/3) to afford compound **11**(232 mg) as a white powder in 81% yield.

^{1}H NMR (400 MHz, $CDCl_3$) δ 8.37 (t, *J* = 10.0 Hz, 8 H), 7.97 – 7.86 (m, 4 H), 7.80 (t, *J* = 4.9 Hz, 4 H), 7.63 – 7.55 (m, 6 H), 7.52 (dd, *J* = 6.0, 4.3 Hz, 6 H), 7.48 – 7.28 (m, 22 H), 6.79 (d, *J* = 3.5 Hz, 4 H), 1.32 (s, 36 H) ; See Supplementary Fig. 52. **^{13}C NMR (100 MHz, $CDCl_3$)** δ 151.48, 141.29, 140.90, 140.75, 139.70, 138.33, 136.40, 131.94, 131.86, 131.76, 131.67, 131.21, 130.11, 129.73, 129.27, 128.90, 128.46, 127.57, 127.37, 126.55, 126.31, 126.20, 126.11, 122.55, 122.43, 122.12, 121.94, 121.78, 35.15, 31.68; See Supplementary Fig. 53. **HRMS (m/z)**: $[M]^+$ calcd. for $C_{112}H_{90}$, 1434.7037; found, 1434.7031; See Supplementary Fig. 54.

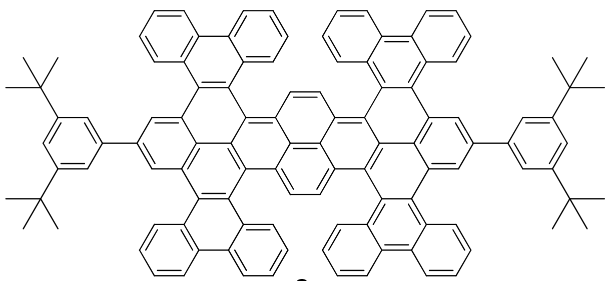

2

Synthesis of 2: To a stirred solution of **11** (142 mg, 0.1 mmol) in dichloromethane (50 mL), a solution of iron(III) chloride (161 mg, 1 mmol) in nitromethane (10 mL) was added dropwise. A nitrogen stream was bubbled through the mixture throughout the course of the reaction. After stirring for another 2 hours at 40°C, the reaction was quenched with methanol (10 mL). The resulting mixture was poured into water and extracted with DCM. The extract was purified by column chromatography (DCM/petroleum ether = 1/3) to afford *s*TT-**2** (28.4 mg, 20%)and another fragment containing other conformers(81 mg, 57%) as an red powder.mp >300°C.

*s*TT-**2**: **^{1}H NMR (500 MHz, 238 K, $CDCl_2CDCl_2$)** δ 9.42 (s, 4 H), 9.12 (s, 4 H), 8.83 (s, 4 H), 8.62 (s, 4 H), 8.38 (s, 4 H), 7.80 (s, 16 H), 7.48 (s, 6 H), 7.06 (s, 4 H), 1.39 (s, 36 H) ; See Supplementary Fig. 55. **^{13}C NMR (125 MHz, $CDCl_2CDCl_2$)** δ 151.69, 132.35, 131.79, 130.16, 129.89, 129.55, 129.19, 128.99, 126.90, 126.78, 126.62, 126.22, 123.77, 123.61, 123.42, 123.33, 123.20, 122.15, 122.05, 121.98, 121.88, 35.04, 31.62; See Supplementary Fig. 56. **HRMS (m/z)**: $[M]^+$ calcd. for $C_{112}H_{82}$, 1426.6411; found, 1426.6402; See Supplementary Fig. 57.

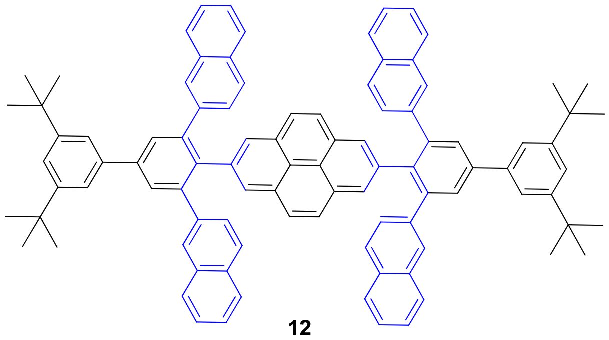

12

Synthesis of 12: A mixture of **8** (208 mg, 0.2 mmol), anthracen-2-ylboronic acid (222 mg, 1 mmol), $Pd(PPh_3)_4$ (23.2 mg, 0.02 mmol) and Na_2CO_3 aqueous solution (2 M, 2 mL) in toluene (30 mL) and EtOH (10 mL) was degassed. The reaction mixture was vigorously stirred under nitrogen at80°C for 24 h. After cooling to room temperature, water (30 mL) was added. The resultant suspension was extracted with dichloromethane (3×30 mL). The combined organic layers were washed with water and then dried over anhydrous $MgSO_4$. The solvent was removed and the crude product was purified by column chromatography (silica gel, DCM/ petroleum ether= 1/3) to afford compound **12**(167mg) as a white powder in 68% yield.

^{1}H NMR (400 MHz, $CDCl_3$) δ 7.90 (s, 4 H), 7.83 (s, 4 H), 7.71 (d, *J* = 7.3 Hz, 4 H), 7.69 (s, 4 H), 7.61 (d, *J* = 7.5 Hz, 4 H), 7.54 (s, 4 H), 7.49 (s, 2 H), 7.43 – 7.33 (m, 12 H), 7.30 (d, *J* = 8.6 Hz, 4 H), 6.96 (d, *J* = 8.7 Hz, 4 H), 1.38 (s, 36 H) ; See Supplementary Fig. 58. **^{13}C NMR (125 MHz, $CDCl_3$)** δ 151.40, 142.80, 142.05, 140.12, 139.87, 137.78, 136.64, 133.21, 131.77, 130.28, 129.69, 128.66, 128.42, 128.37, 127.88, 127.55, 127.07, 126.79, 125.84, 125.66, 122.69, 121.89, 35.07, 31.58; See Supplementary Fig. 59. **HRMS (m/z)**: $[M]^+$ calcd. for $C_{96}H_{82}$, 1234.6411; found, 1234.6405; See Supplementary Fig. 60.

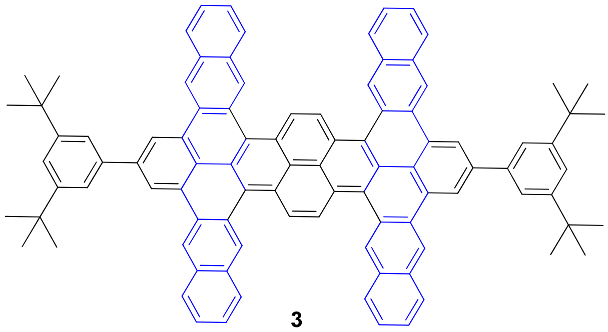

3

Synthesis of 3: To a stirred solution of **12** (123 mg, 0.1 mmol) in dichloromethane (50 mL), a solution of iron(III) chloride (161 mg, 1 mmol) in nitromethane (10 mL) was added dropwise. A nitrogen stream was bubbled through the mixture throughout the course of the reaction. After stirring for another 2 hours at 40°C, the reaction was quenched with methanol (10 mL). The resulting mixture was poured into water and extracted with DCM. The extract was purified by column chromatography (DCM/petroleum ether = 1/3) to afford **3** (96 mg, 78%) as an red powder.mp >300°C. ^{1}H NMR spectra showed low resolution due to aggregation.

^{1}H NMR (500 MHz, $CDCl_2CDCl_2$) δppm 8.72 (s, 1 H), 8.61 (s, 1 H), 8.53-8.33 (m, 4 H), 8.30 (s, 1 H), 8.23-8.12 (m, 2 H), 8.08 (s, 1 H), 7.95 (s, 1 H), 7.93-7.75 (m, 8 H), 7.75-7.52 (m, 8 H), 7.46 (s, 1 H), 7.42-7.30(m, 2 H), 7.09 (s, 1 H), 7.05-6.90 (m, 2 H), 6.77 (s, 1 H), 6.47 (s, 1 H), 6.36 (s, 1 H), 6.23 (s, 1 H), 5.62 (s, 1 H), 1.77 (d, *J* = 15.2 Hz, 36 H) ; See Supplementary Fig. 61. **^{13}C NMR (125 MHz, $CDCl_2CDCl_2$, 378 K)** δ ppm 151.54, 142.21, 141.77, 141.64, 132.76, 132.24, 131.83, 131.67, 129.60, 129.48, 129.36, 128.86, 128.19, 128.05, 127.63, 127.25, 127.15, 126.82, 126.14, 125.93, 125.07, 124.35, 123.18, 122.84, 122.62, 121.77, 121.71, 121.38, 35.27, 31.99; See Supplementary Fig. 62. **HRMS (m/z)**: $[M]^+$ calcd. for $C_{96}H_{74}$, 1226.5785; found, 1226.5777; See Supplementary Fig. 63.

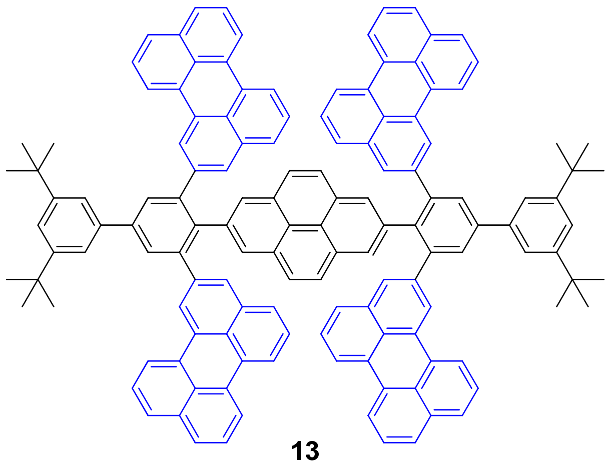

13

Synthesis of 13: A mixture of **8** (208 mg, 0.2 mmol), 4,4,5,5-tetramethyl-2-(perylen-2-yl)-1,3,2-dioxaborolane (378 mg, 1 mmol), $Pd(PPh_3)_4$ (23.2 mg, 0.02 mmol) and Na_2CO_3 aqueous solution (2 M, 2 mL) in toluene (30 mL) and EtOH (10 mL) was degassed. The reaction mixture was vigorously stirred under nitrogen at 80°C for 24 h. After column chromatography (silica gel, DCM/ petroleum ether= 1/1), 221 mg compound **13** was obtained as a yellow powder at 64% yield.

^{1}H NMR (500 MHz, $CDCl_2CDCl_2$, 378 K) δ 8.08 (s, 4 H), 8.05 (d, *J* = 7.5 Hz, 4 H), 7.99 (s, 4 H), 7.96 (s, 4 H), 7.93 (d, *J* = 7.5 Hz, 4 H), 7.66 (d, *J* = 1.5 Hz, 4 H), 7.60 (s, 4 H), 7.56 (d, *J* = 7.6 Hz, 4 H), 7.55 (s, 2 H), 7.52 (d, *J* = 8.1 Hz, 4 H), 7.49 (s, 4 H), 7.41 (t, *J* = 7.8 Hz, 4 H), 7.32 (d, *J* = 8.2 Hz, 4 H), 7.18 (d, *J* = 8.1 Hz, 4 H), 7.05 (t, *J* = 7.8 Hz, 4 H), 7.00 (t, *J* = 7.8 Hz, 4 H), 1.47 (s, 36 H) ; See Supplementary Fig. 64. **^{13}C NMR (125 MHz, $CDCl_2CDCl_2$)** δ 151.48, 142.57, 142.08, 140.07, 139.80, 138.29, 137.09, 134.66, 134.41, 131.22, 131.05, 130.87, 130.61, 130.53, 129.04, 128.86, 128.77, 128.26, 127.71, 127.67, 127.53, 127.26, 127.12, 126.48, 126.28, 125.34, 123.15, 122.96, 121.94, 121.57, 120.30, 120.01, 119.98, 34.98, 31.62; See Supplementary Fig. 65. **HRMS (m/z)**: $[M]^+$ calcd. for $C_{136}H_{98}$, 1730.7663; found, 1730.7663; See Supplementary Fig. 66.

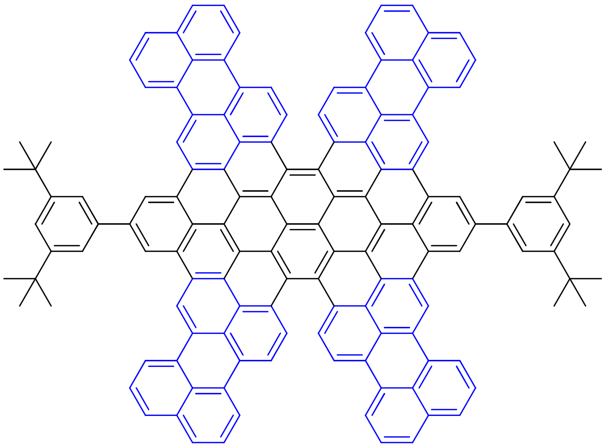

4

Synthesis of 4. To a stirred solution of **13** (173 mg, 0.1 mmol) in dichloromethane (50 mL), a solution of iron(III) chloride (161 mg, 1 mmol) in nitromethane (10 mL) was added drop wise. Nitrogen stream was bubbled through the mixture throughout the course of the reaction. After stirring for another 2 hours at 40°C, the reaction was quenched with methanol (50 mL).Deeply colored solid precipitated from the mixture. After filtration the crude product was washed with a large amount of methanol and hexane, and finally washed with mixture of hexane and acetone (v/v = 10:1). The crude product was further purified by flash column chromatography (deactivated silica gel, CH_2Cl_2/ MeOH = 5/1), the concentrated sample was further washed by Et_2O and CH_2Cl_2 and dried in vacuum at 80°C for 2 days to yield the target product **4** as a black solid in 51% yield (87 mg). No NMR spectrum for aromatic protons can be recorded at room temperature even at low temperature (-100 °C) due to the paramagnetic properties and strong aggregation. The purity was further determined by HPLC analysis with Shim-Pack PREP-SIL column by using different eluents. Under variable conditions, only one elution peak (peak tailing due to the strong absorption on silicon) was observed, indicating high purity of this compound; Supplementary Fig. 67.

HRMS (m/z): $[M]^+$ calcd. for $C_{136}H_{82}$, 1714.6411; found, 1714.6413; See Supplementary Fig. 68.
Elemental Analysis: calcd for $C_{136}H_{82}$: C, 95.18; H, 4.82; found: C, 95.02; H, 4.86%.
